# Benefits and Risks of Native and Exotic Biological Control Agents Used in Latin America and the Caribbean: Performance of 1099 Arthropod Natural Enemies

**DOI:** 10.1007/s13744-026-01412-8

**Published:** 2026-07-28

**Authors:** Joop C. van Lenteren, Vanda H. P. Bueno

**Affiliations:** 1 Lab of Entomology, Dep of Plant Sciences, Wageningen Univ, Wageningen, The Netherlands; 2 Lab of Biological Control, Dep of Entomology, Federal Univ of Lavras, Lavras, MG Brazil

**Keywords:** Predators, Parasitoids, Environmental impact assessment, Non-target effects, Zoophytophagous Miridae, *Harmonia axyridis*

## Abstract

The risks of negative non-target effects caused by the use of biological control agents to control pests have been a hotly debated issue since the 1980s. However, these discussions are often limited to a few specific case studies. Large-scale region-wide evaluations of risks and benefits of releases of natural enemies are rare. We tracked the names of 1099 biocontrol agents used in arthropod biocontrol in Latin America and the Caribbean, and ranked them according to origin (native or exotic), to their role in pest reduction (significant or not) and negative non-target effects. About 40% of the agents used in this region were of exotic origin. Depending on the type of programme—natural, conservation, classical or augmentative biocontrol—the percentages of natural enemies attaining significant pest reduction were 40 up to 100. Hence, many natural enemies provided benefits in the form of healthy, sustainable and environmentally friendly pest management. The documented risk associated with the use of exotic natural enemies appears to be low, although minor or poorly monitored non-target effects may have gone undetected. In order to limit occurrence of negative non-target effects in the future, first an inventory should be made of native natural enemies including an assessment of whether one or more of these species are able to sufficiently reduce pest populations. Only if effective native biocontrol agents are not found, the use of an exotic natural enemy should be considered after performing an environmental impact assessment indicating a low risk for negative side effects.

**Supplementary Information:**

The online version contains supplementary material available at 10.1007/s13744-026-01412-8.

## Introduction

Quantitative data about the number of natural enemies used in biological control (biocontrol) causing negative non-target effects are rare, while the debate about this issue generated many papers (e.g. Heimpel and Cock 2018). Data in the book *Biological Control in Latin America and The Caribbean: Its Rich History and Bright Future* (van Lenteren et al. 2020) provide information about hundreds of natural enemies used in this region. We used these data to try to answer the question how many native and exotic biocontrol agents provided benefits in the form of pest reduction or had a negative effect on non-target organisms.

It is only until very recently that data became available about the extent of use of all types of biocontrol in Latin America, although it was generally known among scientists and biocontrol practitioners in this region that many native and exotic natural enemies have been and are applied on a large scale. In this paper, we will distinguish four types of biocontrol. In *natural biological control* (NBC), pest organisms are reduced by naturally occurring beneficial organisms without human assistance. In *conservation biological control* (ConsBC), one aims to protect and stimulate the performance of naturally occurring natural enemies. *Classical biological control* (CBC) is the introduction of exotic beneficial organisms, usually from a pest’s area of origin, to permanently reduce or eliminate the impact of an exotic organism in a new area where it invaded. Finally, *augmentative biological control* (ABC) is the production and release of large numbers of native or exotic biocontrol agents to obtain direct control of the pest without aiming at a long-lasting effect.

Data about the number of exotic species imported in Latin America and used in CBC were available through the BIOCAT database maintained by CABI (Greathead and Greathead 1992; Cock et al. 2016), but this database does not provide information about the areas under CBC. Areas under various types of biocontrol and numbers of species used were not reliably estimated until 2020 (van Lenteren and Bueno 2003; van Lenteren et al. 2020). A reason for this lack of knowledge was that a lot of information about biocontrol was only available in reports and papers in Spanish or Portuguese, which were often difficult to obtain. Therefore, we embarked earlier on a project with the aim to collect, translate and summarise this information with the help of biocontrol researchers in Latin America, and this resulted in an English and Spanish version of the above-mentioned book (van Lenteren et al. 2020, 2021a). The information provided in this book is used in this paper to compile information about benefits and risks of use of native and exotic biocontrol agents. Although the book lists hundreds of biocontrol projects, we know that the data are not complete, because not all Latin American countries provided information about all cases of biocontrol. Still, when considering these limitations, a representative picture can be constructed about the role of native and exotic biocontrol agents in this region.

Biocontrol of arthropod pests with arthropod natural enemies has been practiced in Latin America since the end of the nineteenth century. The first, short overview of biocontrol in this region was published in 1973 (Hagen and Franz 1973), followed by a more extensive one in 1999 (Altieri and Nichols 1999). Both overviews mainly presented data about CBC. In 2003, van Lenteren and Bueno provided an updated review and included information about ABC, as well as data about areas treated with arthropod biocontrol agents. The latter review, when compared with data in Hagen and Franz (1973), showed that the number of countries using CBC was the same, but that many more countries were using ABC in 2000. For the most recent overview of the history, current situation and key projects of biocontrol in Latin America, books and review papers on biocontrol and national and international organisations working on biocontrol in this region we refer to van Lenteren et al. (2020, 2021a). Information in this book showed that biocontrol was used on a much larger scale and in more countries than earlier known and also demonstrated the large areas under different types of biocontrol. Currently, use of biocontrol is growing quickly and now this region has the largest area under biocontrol worldwide (see e.g. Mason 2021; van Lenteren et al. 2025). van Lenteren and Cock (2020) mentioned that in 2019, 31 million hectares were under CBC in 29 countries, 31.5 million hectares under ABC in 27 countries, 0.5 million hectares under ConsBC in 13 countries and 2 million hectares under NBC in 19 countries in Latin America. The data for the agricultural and forest areas under CBC and ABC are reasonably reliable, but the areas under ConsBC and NBC are presumably seriously underestimated as many countries did not report about these types of biocontrol. During the past decade, particularly the area under ABC has increased fast and our most recent estimate indicates that it was applied on 62 million ha in 2024 (van Lenteren et al. 2025).

For reviews of historical developments in arthropod biocontrol in this region, we refer to the country-specific chapters in van Lenteren et al. (2020, 2021a). Testing of native natural enemies started as early as 1884 in Venezuela, where a hymenopteran parasitoid was evaluated for control of migratory locusts (Vásquez et al. 2020). During the first 100 years, the use of biocontrol was considered very safe (Hajek et al. 2016). Although extensive environmental risk analyses were not demanded before import and release of exotic natural enemies during this period, the majority of biocontrol researchers had ecological and taxonomical knowledge, were aware of such risks and took these into account when evaluating exotic natural enemy species before introduction and release. The situation concerning risk assessment changed in the 1980 s as a result of publications by Howarth (1983, 1991) who illustrated non-target effects caused by the release of exotic natural enemies for use in CBC programmes in Hawaii. A characteristic of successful CBC is that the exotic natural enemy establishes in the new environment and reduces the pest below the economic damage level. However, establishment might be problematic if the natural enemy shows negative side effects because it will be difficult or impossible to eradicate the natural enemy (Simberloff and Stiling 1996). Although Howarth’s interpretation of data were seriously criticised (Follett and Duan 2000), the issue of environmental impact assessment of natural enemies before their release in a new environment gained increasing attention (Bigler et al. 2006); Paula et al. 2021; Peterson 2025). As a result of this awareness, environmental impact assessments have been developed and are now often applied worldwide before import and release of an exotic natural enemy.

Initially, FAO (1996) together with the International Organisation for Biological Control (IOBC) ratified an international Code of Conduct for the Import and Release of Biological Control Agents, which was expanded during the following decades to a set of guidelines and standards for use of exotic biocontrol agents, IPSM3 (FAO 2005). In the same period, many countries drafted national legislation and regulation procedures in collaboration with universities and international bodies such as the European (EPPO) and North American (NAPPO) Plant Protection Organization. Examples of such procedures are the following EPPO guidelines: (1) first import of exotic biological control agents for research under contained conditions (EPPO 2023), (2) import and release of non-indigenous biological control agents (EPPO 2024) and (3) decision-support scheme for import and release of biological control agents of plant pests (EPPO 2018). The situation concerning regulation of import and release of exotic organisms in several Latin America countries is mentioned in Colmenarez et al. (2023), and the authors plea for more consistent and similar guidelines in this region. Sadly, guidelines, legislation and registration procedures widely vary among countries and world regions and urgently need to be harmonised (Peterson 2025).

In this paper, quantitative information will be provided about native and exotic natural enemies tested and used for biocontrol in Latin America, as well as their benefits in contributing to pest management and the negative side effects they may have caused.

## Material and Methods

### Material

We used two sources to find out which natural enemies have been and are currently used in biocontrol of invertebrate pests. The first source is the book on biocontrol in Latin America and the Caribbean (van Lenteren et al. 2020), which contains information about native and exotic natural enemies used in all types of biocontrol in this region. The second source concerns the BIOCAT database (Cock et al. 2016; Cock 2019), which is restricted to exotic natural enemies used in CBC.

### Methods

First, we collected all names of species (animal pests, diseases, weeds, crops and natural enemies) mentioned in van Lenteren et al. (2020). Next, we made a separate list of all biocontrol agents (both macro- and microbial agents). Subsequently, we selected the parasitoids and predators tested and used for biocontrol of invertebrates. Then, we classified the parasitoids and predators according to origin, i.e. exotic or native, playing a role in pest reduction or not in different types of biocontrol (CBC, ABC, ConsBC and NBC), and according to whether negative non-target effects had been reported. As for the ranking of negative side effects, it should be realised that up to about 50 years ago the results of releases pertaining causing negative effects of biocontrol agents were usually not monitored. We classified a species as “playing a role in pest reduction” when authors mentioned that a natural enemy significantly reduced the pest population, even if complete control was not obtained. The classifications of playing a role in pest control and of causing negative side effects are based on secondary evidence. In most cases we did not have the original experimental data available to check whether the classifications were correct, which introduces a source of uncertainty.

Although we have checked the tables for species synonyms and also tried to find out if the species listed either as sp. or as spp. might be the same, we cannot exclude that there still is some overlap in species names and that, thus, the total number of species is somewhat lower than mentioned in the tables below.

When analysing the BIOCAT (Cock et al. 2016; Cock 2019) database, we applied the same classification procedure.

## Results and Discussion

### Natural Enemies Listed in the Book “Biological Control in Latin America and The Caribbean: Its Rich History and Bright Future” (van Lenteren et al. 2020)

**Fig. 1:**
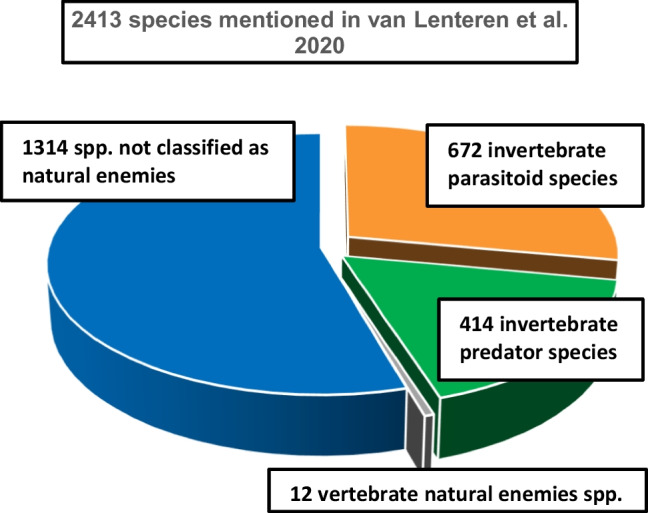
Number of natural enemy species and species not classified as natural enemies mentioned in van Lenteren et al. (2020)

The total number of species mentioned in van Lenteren et al. (2020) amounts to 2413, among which we found 1099 natural enemies (Fig. 1; details in supplementary material, Tables SI1–SI4). Of these natural enemies, 12 species were vertebrates (fish, amphibians, reptiles, birds and mammals) and 1087 species were invertebrates, which could be subdivided into 673 species of parasitoids and 414 species of predators.

**Fig. 2:**
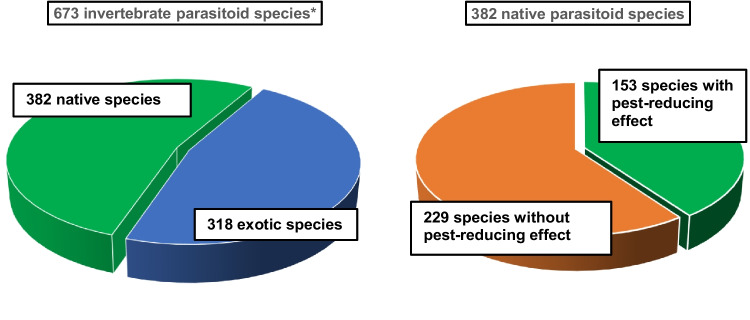
Number of native and exotic parasitoid species tested in biological control projects in Latin America and the Caribbean, and number of native parasitoid species with and without a pest-reducing effect. *The total number of native and exotic species of parasitoids is lower than the sum of the subcategories, because some species are exotic in one country and native in another

The ***parasitoid*** category comprised 382 ***native species*** (Fig. 2; details in supplementary material, Tables SI2 and SI4). Many of these species were found during natural enemy exploration projects. Forty percent of these species did have a pest-reducing effect in NBC, ConsBC or ABC, while 60% was classified as having no pest-reducing effect or as effect unknown because no results regarding their use were mentioned. Four of the native parasitoid species were classified as potentially causing a negative effect: they are hyperparasitoids killing primary parasitoids and, thus, may limit the beneficial role of the primary parasitoids by reducing their population size. Based on knowledge about multitrophic systems in natural and agricultural ecosystems, we expect there will be many more native hyperparasitoids in Latin America, though they were not listed in the book. As these hyperparasitoids have not been targeted for use in biocontrol projects, we have not listed them as species causing negative effects.

**Fig. 3:**
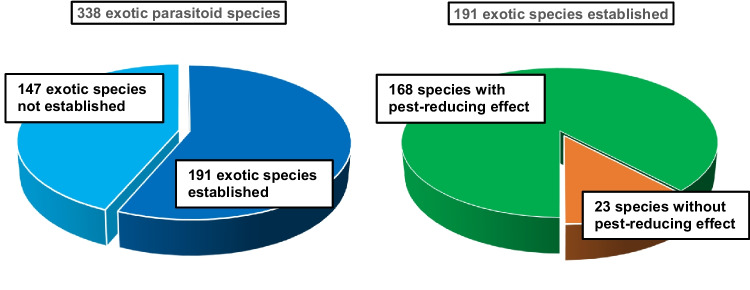
Number of exotic parasitoid species tested in biological control projects in Latin America and the Caribbean, number of exotic species that did establish or not, and number of established exotic parasitoid species with and without a pest-reducing effect

Of the 338 ***exotic parasitoid*** species, 43% of the species did establish, whereas 57% did not establish or establishment is unknown (Fig. 3; details in supplementary material, Tables SI2 and SI4). The percentage of established exotic parasitoids having a pest-reducing effect was 88. Most of these species were introduced in CBC programmes, but quite a large number of species are also used in ABC. The percentage of exotic natural enemies that established but did not have a pest-reducing effect was 12 and is, thus, low compared to the percentage of native parasitoids without pest-reducing effect. The explanation for the large difference in the percentage of parasitoids with pest-reducing effect between exotic (88%) and native species (40%) might be that exotic parasitoids were screened carefully before being introduced to a new area and it was often known that these parasitoids were effective in other regions at previous introductions, whereas native parasitoids found in nature or in a crop were likely quickly tested on a pest or in a crop without much basic research. Exotic parasitoids causing negative non-target effects were not mentioned in the book.

The ***total number of predator species*** found in van Lenteren et al. (2020) is 426 (details in supplementary material, Tables SI3 and SI4). Among the ***native predators***, ten species of ***vertebrate predators*** were listed, such as birds, reptiles and predatory fish, which all had a pest-reducing effect, while negative non-target effects were not mentioned for any of the vertebrate predators (Table SI4).

**Fig. 4:**
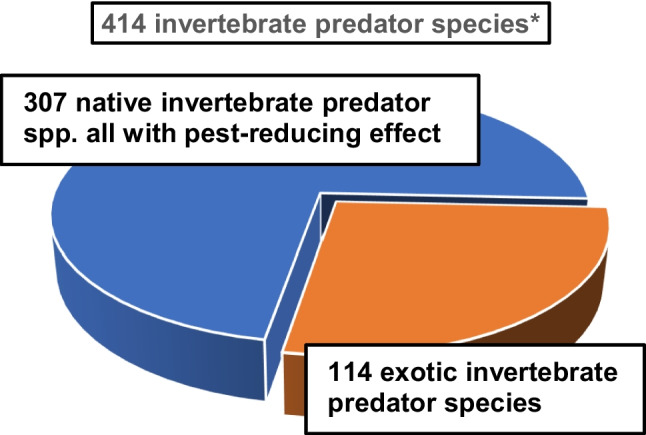
Number of native and exotic predator species tested in biological control projects in Latin America and the Caribbean. *The total number of native and exotic species of predators is lower than the sum of the subcategories, because some species are exotic in one country and native in another

Three hundred and seven species of ***native invertebrate predators*** were listed, which all had a pest-reducing effect (Fig. 4). Documented negative non-target effects were not found for any of the species in van Lenteren et al. (2020), but potential non-target effects were mentioned for zoophytophagous mirid predators after plant and fruit damage caused by the zoophytophagous mirid species *Nesidiocoris tenuis* Reuter (Hemiptera: Miridae) was found in Europe (Urbaneja et al. 2009). However, damage as a result of plant feeding by Miridae has not yet been recorded to occur in Latin America, and, interestingly, tests with three Neotropical mirid species (*Campyloneuropsis infumatus* (Carvalho), *Engytatus varians* (Distant), and *Macrolophus basicornis* (Stal) (Hemiptera: Miridae)) showed that plant and fruit injury did occur but at a much lower level than was observed for the European mirid *N. tenuis* (Silva et al. 2017). Moreover, the low incidence of plant and fruit injury caused by these Neotropical species did not result in economic loss (van Lenteren et al. 2018). As yet, we have no explanation for the large difference in lack of pest-reducing effect found for parasitoids and predators, although predation is a phenomenon observed easier than parasitism.

The ***number of exotic predators*** introduced in Latin America amounts to 116 (Table SI4). Two species of ***vertebrate predators*** were introduced into this region before biocontrol scientists started their work. These early introductions of exotic species for pest control illustrate the problems such releases may cause if a serious environmental impact analysis is not carried out before import and release. The first case was the import of the cane toad, *Rhinella marina* (L.) (Anura: Bufonidae) around 1830 on several Caribbean islands to control insect pests in sugar cane. The second case concerns the import of the Indian mongoose, *Herpestes auropunctiatus* (Hodgson) (Carnivora: Herpestidae) in 1872 on Jamaica and later on other Caribbean islands for control of *Rattus rattus* (L.) (Rodentia: Muridae) in sugar cane. The mongoose not only reduced rat populations, but also damaged crops, consumed amphibians, reptiles, ground-nesting birds and served as a rabies reservoir. In both cases, these vertebrate predators did contribute to pest control but also caused negative effects (Lever 2001).

**Fig. 5:**
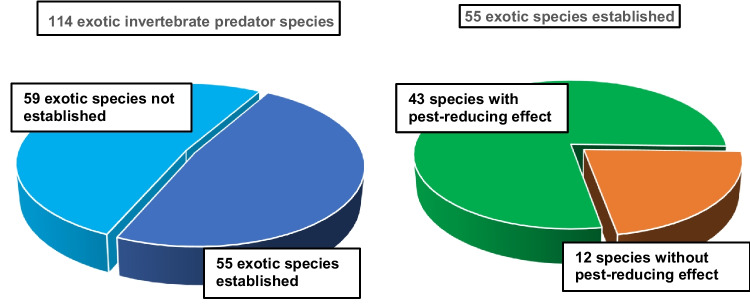
Number of exotic invertebrate predator species tested in biological control projects in Latin America and the Caribbean, number of exotic species that did establish or not, and number of established exotic invertebrate species with and without a pest-reducing effect

Of the 114 species of ***exotic invertebrate predators*** introduced into this region, 59 species did not establish, or establishment is not documented (Fig. 5; details in supplementary material, Tables SI3 and SI4). Fifty-five species established and of these, 78% have a pest-reducing effect, while 22% did not contribute to significant pest reduction (Fig. 5; details in supplementary material, Tables SI3 and SI4).

The percentage of exotic predators with a pest-reducing effect is slightly lower than that of exotic parasitoids. The total number of exotic predators introduced in Latin America is much lower than the number of introduced parasitoids. This difference is not specific for the Latin American region, as it is also documented for other world regions (Mason 2021). A likely explanation for this difference is that predators are in general more polyphagous than parasitoids, and may, thus, cause negative non-target effects more often than parasitoids, resulting in the decision not to release exotic predators. The issue of using generalist versus specialist natural enemies is a much discussed issue in the biocontrol and ecological literature (Taylor and Snyder 2021). Some biocontrol practitioners prefer generalist natural enemies, because they may attack different pest species in the same crop and can more easily survive on alternative prey when the pest species is scarce (DeClercq 2002). Other practitioners favour the use of specialist natural enemies as they may be better evolved to find and reduce the pest species to lower population levels, and, together with ecologists, they suppose specialists bear lower risks for direct and indirect negative effects (Nedvěd 2026).

One arthropod predator was mentioned to contribute to pest control but also to cause non-target effects in several countries in this region: the ladybird species *Harmonia axyridis* (Pallas) (Coleoptera, Coccinellidae). This species is an effective natural enemy of pest aphids but has a very wide prey range and consumes Hemiptera, Psyllidae, Coccoidea, Chrysomelidae, Curculionidae, Coccinellidae, Lepidoptera and Tetranychidae, feeds on fruit and is a nuisance for humans by hibernating in large numbers within houses (Koch et al. 2006). The species has established in Argentina, Brazil, Chile, Colombia, Ecuador, Mexico, Paraguay, Peru, Uruguay and Venezuela. Argentina, Brazil, Chile and Colombia reported a reduction in population size of native coccinellids after introduction of *H. axyridis* (for details, see van Lenteren et al. 2021b).

### Natural Enemy Introductions into Latin America Mentioned in the BIOCAT Database

The second source we used to trace information about negative non-target effects concerns the BIOCAT database (Cock et al. 2016; Cock 2019), which is restricted to cases of CBC.

The Centre for Agriculture and Biosciences International (CABI) maintains the database BIOCAT documenting the deliberate introductions of natural enemies for biocontrol since the 1890 s (Cock et al. 2016). We used version 2010.3 (Cock 2019) to compile lists of classical biocontrol introductions for Latin America which are published in van Lenteren and Cock (2020). Although this version is not up to date and cannot be relied upon for the last decade or two, it contains the majority of classical biocontrol introductions, the target pests, the number of establishments of the natural enemy species and the successes.

**Fig. 6:**
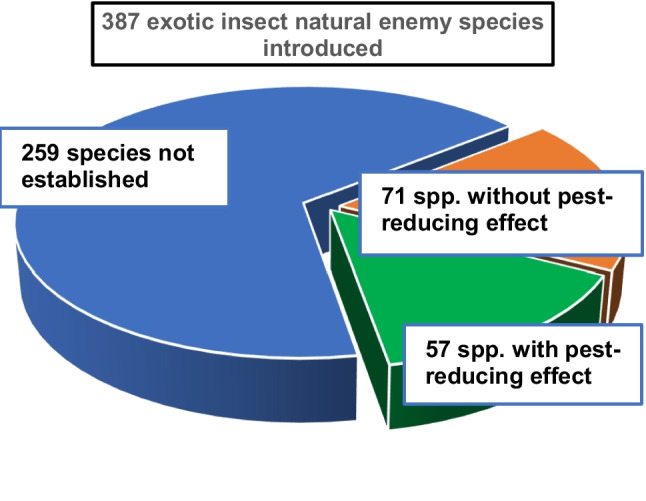
BIOCAT2010.3 data on classical biological control of insect pests using exotic insect biological control agents in Latin America and the Caribbean (Cock 2019)

A total of 387 species of ***exotic natural enemies*** were introduced in one or more Latin American countries (BIOCAT2010.3) of which 128 (33%) species established. Fifty-seven (45%) of the established species caused a significant pest-reduction effect (Fig. 6, details in supplementary material, Table SI5). Names of all species can be found in Table 32.5 of van Lenteren and Cock (2020). None of the established species was said to have caused negative non-target effects. The percentage of releases of species with a pest-reducing effect is higher for the data collected in van Lenteren et al. (2020) than for the BIOCAT data. This might be explained as follows: (1) the BIOCAT classification of success is based on quantitative data, whereas the classification in most of the book chapters is qualitative and quantitative data are not presented, (2) the book chapters also provide information about releases made during the past 20–25 years, whereas data about recent releases are not yet included in BIOCAT, (3) the book chapters list all invertebrate natural enemies (including predatory mites) while BIOCAT only includes insect natural enemies, and, finally (4) the book chapters register natural enemies used in all types of biocontrol, while BIOCAT is restricted to CBC.

Almost all natural enemy species mentioned in BIOCAT2010.3 were also mentioned in the country-specific chapters in van Lenteren et al. (2020). Three species mentioned in the BIOCAT database which were introduced in Chile more than 50 years ago for control of pests in forests, were not mentioned in van Lenteren et al. (2020). None of the species listed in BIOCAT caused negative non-target effects, whereas one species (*H. axyridis*) was mentioned to cause negative effects in van Lenteren et al. (2020). This is not unexpected, because *H. axyridis* was introduced for ABC, not for CBC and is, thus, not cited in the BIOCAT database.

With the BIOCAT data, we are able to find out what the effects are of increasing regulatory oversight, including requiring environmental impact assessments. First of all, it has led to a reduction in the number of introductions of exotic natural enemies since the 1980 s (Fig. 32.1 in van Lenteren and Cock 2020). Secondly, it resulted in increased activities in making inventories of native natural enemies as biocontrol agents and increased research in the role of ConsBC. Also, the impact assessment studies seem to have a positive effect on the percentage of exotic natural enemies establishing after introduction: before 1980 37.7% of natural enemy introductions (*n* = 595) resulted in establishment, whereas after 1980 51.2% of 164 introductions established (Cock et al. 2016).

### How to Explain That Many of the Exotic Species Failed to Establish?

Above, we mentioned several times that a substantial percentage of the exotic natural enemies did not establish after release. There are many reasons to explain failures to establish. In the early period of biocontrol up until the 1980 s, often the “hit and miss” (DeBach 1964) or “shotgun” approach was followed with the idea that release of every biocontrol agent that you can get hold of and is not expected to cause negative side effects should be released with the expectation that something might work. In Latin America and the Caribbean this was often the approach in the early days of the CABI station on Trinidad (Cock 1985). As a result of this approach, failures to establish may have been the result of too low numbers, lack of synchronisation between the moment of release and absence of the suitable stages of the pest species, lack of sufficient knowledge of the agroecosystem including climate conditions where the natural enemies are released, and insufficient information about already present natural enemies preventing establishment. Also, poor and long transport conditions such as by ship, as well as bureaucratic and time-consuming import procedures at customs could have caused high mortality resulting in release of few individuals and poor quality of the imported natural enemies. It is, therefore, rewarding to observe that since the 1980 s, when more basic studies on exotic species targeted for release were demanded than in the pre-1980 period, the percentage of species established has increased (Cock et al. 2016). Examples of pre-release evaluation of natural enemies resulting in the selection of only one or a few, usually the most host- or prey-specific natural enemies can be found in Mason (2021). Kairo et al. (2000) provide an important case study about selection of natural enemies for control of the hibiscus mealybug, *Maconellicoccus hirsutus* Green (Hemiptera: Pseudococcidae) in the Caribbean.

### Negative Non-target Effects Caused by Exotic Natural Enemies and the Need for Environmental Risk Assessment

None of the native natural enemies was found to create negative non-target effects, while one exotic predator introduced for ABC did cause negative effects in at least four countries in this region (see above for details). The fact that we found only one natural enemy introduction resulting in serious negative side effects seems in line with findings of other authors mentioning few or no negative side effects after release of exotic biocontrol agents (Hajek et al. 2016 and references therein). Nevertheless, we expect that minor effects such as small reductions in population densities of native natural enemies or non-target host/prey species often may have gone unnoticed (Lynch et al. 2001). Also, we did not have original data about biocontrol experiments and had to rely on secondary sources, which created a significant source of uncertainty.

In the early days of modern biocontrol, follow-up studies after releasing a natural enemy often only considered its pest control effect without paying much attention to side effects. Currently, potential negative side effects are evaluated before release, and it is more common to execute post-release evaluation of possible side effects. That said, most biocontrol scientists have always been well aware of potential problems natural enemies might create, preferred to use more selective parasitoids over polyphagous predators and did not advise release of biocontrol agents that were known to have caused problems in other areas. In addition, if serious negative effects had resulted after the release of certain biocontrol agents in Latin America or the Caribbean, we expect these would have been observed and published, like in the case of the two imported vertebrate predators that created problems in this region.

In order to reduce occurrence of negative non-target effects in the future, we propose to first make an inventory of native natural enemies of a pest organism and assess if one or more of these are able to sufficiently reduce pest populations. Only if native natural enemies are not found, reduce a pest insufficiently or appear to be extremely difficult and costly to mass produce, the use of an exotic natural enemy should be considered after an environmental impact assessment has been performed, indicating a low risk for negative side effects. Unfortunately, many countries still demand rather different methods for assessing risks (Peterson 2025). Some level of harmonization has been obtained in Europe, initially through the activities of a working group of IOBC, and later as a result of work by an EPPO-IOBC working group (Bale 2011). Environmental risk assessments used in Latin America and the Caribbean are very different (van Lenteren et al 2020). In addition to harmonization, Heimpel and Wright (2026) stress the need of “ Incorporating ecological realism into biological control risk assessment”, to enhance decision making.

A serious problem is that both issues—environmental risk assessment and post-release studies—demand skilled researchers, clear frameworks, sufficient funding and competent authorities able to review the dossiers, which are often lacking in many Latin American and Caribbean countries.

## Conclusions

The Latin American and Caribbean biocontrol literature provides many examples of the use of native and exotic natural enemies, lists introduced exotic species that were not found in post-release studies and, thus, apparently did not establish, and refers to the many successes. Hardly any information about observed negative non-target effects was mentioned, though this might partly be explained by the lack of post-release studies in many biocontrol projects and the difficulty to observe relatively small, indirect non-target effects. This stresses the need of post-release studies in future biocontrol projects in these countries, which often have rich and complex biodiverse ecosystems.

Since the start of modern biocontrol approximately 150 years ago in Latin America at least 1099 species of arthropod natural enemies have been evaluated for their role in pest management. Many species contributed to significant reductions in pest populations and, thus, provided an important benefit in the form of sustainable pest management. This approach offers an environmentally sound alternative to conventional synthetic chemical pest control, minimizing pollution and reducing risks to field workers, consumers, and non-target organisms.

## Supplementary Information

Below is the link to the electronic supplementary material.

ESM 1 (PDF 1.11 MB)

ESM 2 (PDF 568 KB)

ESM 3 (PDF 469 KB)

ESM 4 (PDF 154 KB)

## Data Availability

All data used for the analyses and the results presented in this paper are provided in the tables in theSupplementary Information.
