# Supplementary material for: Benefits and Risks of Native and Exotic Biological Control Agents Used in Latin America and the Caribbean: Performance of 1099 Arthropod Natural Enemies
Source: Neotrop Entomol. 2026 Jul 28;55(1):69. doi: 10.1007/s13744-026-01412-8 (PMC13415494; doi:10.1007/s13744-026-01412-8)
Supplement: Supplementary file 1 — (PDF 1.11 MB) [file 13744_2026_1412_MOESM1_ESM.pdf]

**Table SI1. Benefits and risks of endemic and exotic biological control agents used in Latin America and the Caribbean: performance of 1099 arthropod natural enemies. Joop C. van Lenteren, Vanda H. P. Bueno, Wageningen University, Laboratory of Entomology, Department of Plant Sciences, 6700 AA, Wageningen, The Netherlands, joop.vanlenteren@wur.nl**<br><br>**Organisms mentioned in van Lenteren et al. 2020: "Biological Control In Latin America and The Caribbean: Its Rich History And Bright Future"**	
**Scientific name of organism, author (order, family)**	**Type of organism**
A	
Acacia sp. (Fabales: Fabaceae)	Plant
Acanthocereus pentagonus (L.) Hummelinck (Caryophyllales: Cactaceae)	Plant
Acanthops falcata Stål (Mantodea:Acanthopidae)	Predatory insect
Acanthoscelides obtectus (Say) (Coleoptera: Chrysomelidae)	Herbivorous insect
Acaulona erythropyga Sabrosky (Diptera: Tachinidae)	Parasitoid insect
Acaulona peruviana Tns (Diptera: Tachinidae)	Parasitoid insect
Aceratoneuromyia (=*Synthomosphyrum indicum*) *indica* (Silv.) (Hymenoptera: Eulophidae)	Parasitoid insect
Aceria chondrillae Canestrini (Canestrini) (Acari: Eriophyidae)	Herbivorous mite
Aceria guerreronis Keifer (Acari: Eriophyidae)	Herbivorous mite
Aceria sheldoni Ewing (Acari: Eriophyidae)	Herbivorous mite
Acerophagous papayae Noyes & Schauff (Hymenoptera: Encyrtidae)	Parasitoid insect
Acerophagus coccois Smith (Hymenoptera: Encyrtidae)	Parasitoid insect
Acerophagus spp. (Hymenoptera: Encyrtidae)	Parasitoid insects
Achatina fulica (Férrusac) (Gastropoda: Achatinidae)	Herbivorous snail
Achrysochari sp. (Hymenoptera: Eulophidae)	Parasitoid insect
Acmella oppositifolia (Lam.) R.K. Jansen (Asterales:Asteraceae)	Plant
Acontiothespis brevipennis (Saussure) (Mantodea: Acontistidae)	Predatory insect
Acontiothespis concinna (Perty) (Mantodea: Acontistidae)	Predatory insect
Acontiothespis multicolor (Saussure) (Mantodea: Acontistidae)	Predatory insect
Acremonium persicinum (Nicot) W. Gams (Hypocreales: Hypocreaceae)	Antagonistic fungus
Acremonium zonatum (Sawada) W. Gams (Hypocreales: Hypocreaceae)	Antagonistic fungus
Aculops lycopersici Massee (Acarina: Eriophyidae)	Herbivorous mite
Acyrthosiphon kondoi Shinji (Hemiptera: Aphididae)	Herbivorous insect
Acyrthosiphon pisum Harris (Hemiptera: Aphididae)	Herbivorous insect
Adalia bipunctata L. (Coleoptera: Coccinellidae)	Predatory insect
Adelencyrtus moderatus (How.) (Hymenoptera: Encyrtidae)	Parasitoid insect
Adelencyrtus odonaspidis Fullaway (Hymenoptera: Encyrtidae)	Parasitoid insect
Aedes aegypti (L.) (Diptera: Culicidae)	Harmful insect
Aegorhinus nodipennis (Hope) (Coleoptera: Curculionidae)	Herbivorous insect
Aegorhinus superciliosus (Guérin) (Coleoptera: Curculionidae)	Herbivorous insect
Aenasius vexans (Kerrich) (Hymenoptera: Encyrtidae)	Parasitoid insect
Aeneolamia albofasciata (Lailemand) (Homoptera: Cercopidae)	Herbivorous insect
Aeneolamia flavilatera (Urich) (Homoptera: Cercopidae)	Herbivorous insect
Aeneolamia postica (Walker) (Hemiptera: Cercopidae)	Herbivorous insect
Aeneolamia sp. (Homoptera: Cercopidae)	Herbivorous insect
Aeneolamia spp. (Homoptera: Cercopidae)	Herbivorous insects
Aeneolamia varia (F.) (Homoptera:Cercopidae)	Herbivorous insect

Aeneolamia varia saccharina (Dist.) (Homoptera:Cercopidae)	Herbivorous insect
Aganaspis daci (Weld) (Hymenoptera: Eulophidae)	Parasitoid insect
Aganaspis pelleranoi (Brethes) (Hymenoptera: Eulophidae)	Parasitoid insect
Agasicles hygrophila Selman & Vogt (Coleoptera: Chrysomelidae)	Herbivorous insect
Agathis diversa (Mues.) (Hymenoptera: Braconidae)	Parasitoid insect
Agathis sp. (Hymenoptera: Braconidae)	Parasitoid insect
Agathis stigmatera (Cresson) (Hymenoptera: Braconidae)	Parasitoid insect
Ageniaspis citricola (Logvinovskaya) (Hymenoptera: Encyrtidae)	Parasitoid insect
Ageratina riparia (Regel) K. & R. (= *Eupatorium riparium* Regel) (Asterales: Asteraceae)	Plant
Ageratum conyzoides L. (Asterales: Asteraceae)	Plant
AgMNPV virus (*A. gemmatalis* nucleopolyhedrosis)	Entomopathogenic virus
AgMNPV virus	Entomopathogenic virus
Agonopterix ulicetella (Stainton) (Lepidoptera: Oecophoridae)	Herbivorous insect
Agraulis sp. (Lepidoptera: Nymphalidae)	Herbivorous insect
Agraulis vanillae (L.) (Lepidoptera: Nymphalidae)	Herbivorous insect
Agrobacterium radiobacter (Beijerinck and van Delden) (Rhizobiales: Rhizobiaceae)	Phytopathogenic bacterium
Agrobacterium tumefaciens Smith & Townsend (Rhizobiales: Rhizobiaceae)	Phytopathogenic bacterium
Agrothereuthes diatraeae Myers (Hymenoptera: Ichneumonidae)	Parasitoid insect
Agrotis deprivata Walker (Lepidoptera: Noctuidae)	Herbivorous insect
Agrotis ipsilon (Hufnagel) (Lepidoptera: Noctuidae)	Herbivorous insect
Agrotis sp. (Lepidoptera: Noctuidae)	Herbivorous insect
Agrotis spp. (Lepidoptera: Noctuidae)	Herbivorous insects
Akanthomyces sp. (Hypocreales:Cordycipitaceae)	Entomopathogenic fungus
Alabagrus stigma Brullé (Hymenoptera: Braconidae)	Parasitoid insect
Alabama argillacea (Hueb.) (Lepidoptera: Noctuidae)	Herbivorous insect
Alabama sp. (Lepidoptera: Noctuidae)	Herbivorous insect
Alcaeorrhynchus grandis (Dallas) (Hemiptera: Pentatomidae)	Predatory insect
Alcidion cereicola Fisher (Coleoptera: Cerambycidae)	Herbivorous insect
Aleiodes sp. (Hymenoptera: Braconidae)	Parasitoid insect
Aleurocanthus woglumi (Ashby) (Hemiptera: Aleyrodidae)	Herbivorous insect
Aleuroctonus vittatus (Dozier) (Hymenoptera: Eulophidae)	Parasitoid insect
Aleurocybotus occiduus Russell (Hemiptera: Aleyrodidae)	Herbivorous insect
Aleurodicus cocois (Curtis) (Hemiptera: Aleyrodidade)	Herbivorous insect
Aleurodicus dispersus Russell (Hemiptera: Aleyrodidae)	Herbivorous insect
Aleurodicus juleikae Bondar (Hemiptera: Aleyrodidae)	Herbivorous insect
Aleurodicus pulvinatus Maskell (Hemiptera: Aleyrodidae)	Herbivorous insect
Aleurodicus spp. (Hemiptera: Aleyrodidade)	Herbivorous insects
Aleurothrixus floccossus (Maskell) (Hemiptera: Aleyrodidae)	Herbivorous insect
Aleurotrachelus trachoides Back (Hemiptera: Aleyrodidae)	Herbivorous insect
Aleyrodes proletella (L.) (Hemiptera: Aleyrodidae)	Herbivorous insect
Allium spp. (Asparagales: Amaryllidaceae)	Plants
Allograpta (Diptera: Syrphidae)	Predatory insects
Allograpta (*Fazia*) CR-2 aff. *hians* (Enderlein) (Diptera: Syrphidae)	Predatory insect
Allograpta citri Muesebeck (Diptera: Syrphidae)	Predatory insect
Allograpta exotica (Wiedemann) (Diptera: Syrphidae)	Predatory insect
Allorhogas pyralophagus (Marsh) (Hymenoptera: Braconidae)	Parasitoid insect
Allorhogas sp. (Hymenoptera: Braconidae)	Parasitoid insect

Allotropa citri (Muesebeck) (Hymenoptera: Platigasteridae)	Parasitoid insect
Allotropa sp. (Hymenoptera: Platygasteridae)	Parasitoid insect
Alternanthera philoxeroides Griseb (Caryophyllales: Amaranthaceae)	Plant
Alternaria (Pleosporales: Pleosporaceae)	Phytopathogenic fungi
Alternaria alternata (Keissl) (Pleosporales: Pleosporaceae)	Phytopathogenic fungus
Alternaria dauci (J.G. Kühn) J.W. Groves & Skolko (Pleosporales: Pleosporaceae)	Phytopathogenic fungus
Alternaria eichhorniae Nag Raj & Ponnappa (Pleosporales: Pleosporaceae)	Phytopathogenic fungus
Alternaria longipes (Ellis & Everh.) E.W. Mason (Pleosporales: Pleosporaceae)	Phytopathogenic fungus
Alternaria porri (Ellis) Cif. (Pleosporales: Pleosporaceae)	Phytopathogenic fungus
Alternaria solani Sorauer (Pleosporales: Pleosporaceae)	Phytopathogenic fungus
Alurnus humeralis Rosenberg (Coleoptera: Chrysomelidae)	Herbivorous insect
Amaranthus sp. (Caryophyllales: Amaranthaceae)	Plant
Amaranthus spinosus L. (Caryophyllales: Amaranthaceae)	Plant
Amauromyza maculosa (Malloch) (Diptera: Agromyzidae)	Herbivorous insect
Amazona vittata Boddaert (Psittaciformes: Psittacidae)	Bird
*Amblydromalus limonicus (*Garman & McGregor) (Acari: Phytoseiidae)	Predatory mite
Amblyseiopsis musae Garman (Acari: Phytoseiidae)	Predatory mite
Amblyseius (Acari: Phytoseiidae)	Predatory mites
Amblyseius chungas Denmark & Muma (Acari: Phytoseiidae)	Predatory mite
Amblyseius cucumeris (Oudemans) (Acari: Phytoseiidae)	Predatory mite
Amblyseius herbicolus (Chant) (Acari: Phytoseiidae)	Predatory mite
Amblyseius largoensis (Muma) (Acari: Phytoseiidae)	Predatory mite
Amblyseius obtusus (Koch) (Acari: Phytoseiidae)	Predatory mite
Amblyseius perlongisetus Berlese (Acari: Phytoseiidae)	Predatory mite
Amblyseius sp. (Acari: Phytoseiidae)	Predatory mite
Amblyseius swirskii Athias-Henriot (Acari: Phytoseiidae)	Predatory mite
Amblyseius tamatavensis Blommers (Acari: Phytoseiidae)	Predatory mite
Ameiva atrigularis (L.) (Squamata: Teiidae)	Predatory lizard
Ameiva spp. (Squamata: Teiidae)	Predatory lizards
Amitus bennetti Viggiani & Evans (Hymenoptera: Platygasteridae)	Parasitoid insect
Amitus fuscipennis (MacGown & Nebeker) (Hymenoptera: Platygastridae)	Parasitoid insect
Amitus hesperidum Silvestre (Hymenoptera: Platygastridae)	Parasitoid insect
Amitus sp. (Hymenoptera: Platigastridae)	Parasitoid insect
Amitus spiniferus (Brethes) (Hymenoptera: Plastigastridae)	Parasitoid insect
Amynothrips andersoni O'Neill (Thysanoptera: Phlaeothripidae)	Herbivorous insect
Anacardium (Sapindales: Anacardiaceae)	Plants
Anagrus flaveolus Waterhouse (Hymenoptera: Mymaridae)	Parasitoid insect
Anagrus gonzalezae Triapitsyn (Hymenoptera: Mymaridae)	Parasitoid insect
Anagrus optabilis (Perkins) (Hymenoptera: Mymaridae)	Parasitoid insect
Anagrus urichi Pickles (Hymenoptera: Mymaridae)	Parasitoid insect
Anagyrus (=Apoanagyrus) californicus (Compere) (Hymenoptera: Encyrtidae)	Parasitoid insect
*Anagyrus diversicornis (*Howard) (Hymenoptera: Encyrtidae)	Parasitoid insect
Anagyrus kamali Moursi (Hymenoptera: Encyrtidae)	Parasitoid insect
Anagyrus loecki Noyes and Menezes (Hymenoptera: Encyrtidae)	Parasitoid insect
Anagyrus mangicola Noyes (Hymenoptera: Encyrtidae)	Parasitoid insect
Anagyrus pseudococci (Girault) (Hymenoptera: Encyrtidae)	Parasitoid insect
Anagyrus saccharicola Timb. (Hymenoptera: Encyrtidae)	Parasitoid insect

Anagyrus sp. (Hymenoptera: Encyrtidae)	Parasitoid insect
Anagyrus spp. (Hymenoptera: Encyrtidae)	Parasitoid insects
Ananas comosus (L.) Merr. (Poales: Bromeliaceae)	Plant
Anaphes iole Girault (Hymenoptera: Mymaridae)	Parasitoid insect
Anaphes nitens Girault (Hymenoptera: Mymaridae)	Parasitoid insect
Anastatus sp. (Hymenoptera: Eupelmidae)	Parasitoid insect
Anastatus spp. (Hymenoptera: Eupelmidae)	Parasitoid insects
Anastrepha (Diptera: Tephritidae)	Herbivorous insect
Anastrepha fraterculus (Wiedemann) (Diptera: Tephritidae)	Herbivorous insect
Anastrepha ludens (Loew) (Diptera: Tephritidae)	Herbivorous insect
Anastrepha mombinpraeoptans Sein (Diptera: Tephritidae)	Herbivorous insect
Anastrepha obliqua Macquart (Diptera: Tephritidae)	Herbivorous insect
Anastrepha serpentina (Wiedemann) (Diptera:Tephritidae)	Herbivorous insect
Anastrepha sp. (Diptera: Tephritidae)	Herbivorous insect
Anastrepha spp. (Diptera: Tephritidae)	Herbivorous insects
Anastrepha striata (Schiner) (Diptera: Tephritidae)	Herbivorous insect
Anastrepha suspensa (Loew) (Diptera: Tephritidae)	Herbivorous insect
Anatrachyntis (= Pyroderces) rileyi (Wals.)(Lepidoptera: Cosmopterygidae)	Herbivorous insect
*Ancylostomia stercorea (*Zelller) (Lepidoptera: Crambidae)	Herbivorous insect
Anicetus sp. (Hymenoptera: Encyrtidae)	Parasitoid insect
Anisopteromalus calandrae (Howard) (Hymenoptera: Pteromalidae)	Parasitoid insect
Annona cherimola Mill (Magnoliales: Annonaceae)	Plant
Annona muricata L. (Magnoliales: Annonaceae)	Plant
Anoda cristata L. (Malvales: Malvaceae)	Plant
Anolis (Squamata: Dactyloidea)	Predatory lizards
Anomala spp. (Coleoptera: Scarabaeidae)	Herbivorous insects
Anomis sp. (Lepidoptera: Noctuidae)	Herbivorous insect
Anopheles albimanus C.R.G. Wiedemann (Diptera: Culicidae)	Harmful insect
Anopheles punctipennis (Say) (Diptera: Culicidae)	Harmful insect
Anopheles pseudopunctipennis Theobald (Diptera: Culicidae)	Harmful insect
Anopheles spp. (Diptera: Culicidae)	Harmful insects
Anovia circumclusa (Gorham) (Coleoptera: Coccinellidae)	Predatory insect
Anovia punica Gordon (Coleoptera: Coccinellidae)	Predatory insect
Anthonomus eugenii Cano (Coleoptera: Curculionidae)	Herbivorous insect
Anthonomus grandis (Boheman) (Coleoptera: Curculionidae)	Herbivorous insect
Anticarsia gemmatalis Hübner (Lepidotpera: Noctuidae)	Herbivorous insect
Anticarsia sp. (Lepidotpera: Noctuidae)	Herbivorous insect
Antichloris (= *Ceramidia*) *viridis* (Druce) (Lepidoptera: Arctiidae)	Herbivorous insect
Antonina graminis (Maskell) (Hemiptera: Pseudococcidae)	Herbivorous insect
Antrocephalus renalis Wtstn (Hymenoptera: Chalcididae)	Parasitoid insect
Aonidiella aurantii (Maskell) (Hemiptera: Diaspididae)	Herbivorous insect
Aonidiella aurantii Maskell (Hemiptera: Diaspididae)	Herbivorous insect
Apanteles (*Cotesia*) *plutellae* Kurd (Hymenoptera: Braconidae)	Parasitoid insect
Apanteles (*Cotesia*) sp. poss. *marginiventris* (Cress.), (Hymenoptera: Braconidae)	Parasitoid insect
Apanteles (*Glyptapanteles*) sp. (Hymenoptera: Braconidae)	Parasitoid insect
Apanteles (Rhygoplitis) aciculatus (Ashm) (Hymenoptera: Braconidae)	Parasitoid insect
Apanteles angeleti Mues. (Hymenoptera: Braconidae)	Parasitoid insect

Apanteles etiellae Viereck (Hymenoptera: Braconidae)	Parasitoid insect
Apanteles gelechiidivoris Marsh (Hymenoptera: Braconidae)	Parasitoid insect
Apanteles glomeratus (L.) (Hymenoptera: Braconidae)	Parasitoid insect
Apanteles marginiventris (Cress.) (Hymenoptera:Braconidae)	Parasitoid insect
Apanteles sesamiae Cam. (Hymenoptera: Braconidae)	Parasitoid insect
Apanteles sp. (Hymenoptera: Braconidae)	Parasitoid insect
Apanteles spp. (Hymenoptera:Braconidae)	Parasitoid insects
Apanteles talidicica Wlkn (Hymenoptera: Braconidae)	Parasitoid insect
Apanteles thurberiae Muesebeck (Hymenoptera: Braconidae)	Parasitoid insect
Apate monachus Fabricius (Coleoptera: Bostrichidae)	Herbivorus insect
Aphanogmus sp. (Hymenoptera: Ceraphronidae)	Parasitoid insect
Aphelinus abdominalis Dalman (Hymenoptera: Aphelinidae)	Parasitoid insect
Aphelinus albipodus (Hayat and Fatima) (Hymenoptera: Aphelinidae)	Parasitoid insect
Aphelinus asychis (Walker) (Hymenoptera: Aphelinidae)	Parasitoid insect
Aphelinus flavipes Forster (Hymenoptera: Aphelinidae)	Parasitoid insect
Aphelinus gossypii (Timberlake) (Hymenoptera: Aphelinidae)	Parasitoid insect
Aphelinus gossypii Timberlake (Hymenoptera: Aphelinidae)	Parasitoid insect
Aphelinus mali Haldeman (Hymenoptera: Aphelinidae)	Parasitoid insect
Aphelinus varipes Forster (Hymenoptera: Aphelinidae)	Parasitoid insect
Aphidius colemani Viereck (Hymenoptera: Braconidae)	Parasitoid insect
Aphidius ervi Haliday (Hymenoptera: Braconidae)	Parasitoid insect
Aphidius matricariae Haliday (Hymenoptera: Braconidae)	Parasitoid insect
Aphidius pascuorum Marshall (Hymenoptera: Braconidae)	Parasitoid insect
Aphidius picipes (Ness) (Hymenoptera: Braconidae)	Parasitoid insect
Aphidius rhopalosiphi De Stefani (Hymenoptera: Braconidae)	Parasitoid insect
Aphidius smithi Sharma & Subba Rao (Hymenoptera: Braconidae)	Parasitoid insect
Aphidius sp. (Hymenoptera: Braconidae)	Parasitoid insect
Aphidius spp. (Hymenoptera: Braconidae)	Parasitoid insects
Aphidius uzbekistanicus Luzhetzki (Hymenoptera: Braconidae)	Parasitoid insect
Aphidius (Hymenoptera: Braconidae)	Parasitoid insects
Aphidoletes aphidimyza (Rondani) (Diptera: Cecidomyiidae)	Predatory insect
Aphis (Hemiptera: Aphididae)	Herbivorous insects
Aphis craccivora Koch (Hemiptera: Aphididae)	Herbivorous insect
Aphis gossypii Glover (Hemiptera: Aphididae)	Herbivorous insect
Aphis nerii Boyer de Fonscolombe (Hemiptera: Aphididae)	Herbivorous insect
Aphis sorghi Theobald (Hemiptera: Aphididae)	Herbivorous insect
Aphis spiraecola Patch. (Hemiptera: Aphididae)	Herbivorous insect
Aphis sp. (Hemiptera: Aphididae)	Herbivorous insect
Aphytis chrysomphali Mercet (Hymenoptera: Aphelinidae)	Parasitoid insect
Aphytis diaspidis (How) (Hymenoptera: Aphelinidae)	Parasitoid insect
Aphytis diaspidis (How.) (Hymenoptera: Aphelinidae)	Parasitoid insect
Aphytis fuscipennis (How) (Hymenoptera: Aphelinidae)	Parasitoid insect
Aphytis holoxanthus DeBach (Hymenoptera: Aphelinidae)	Parasitoid insect
Aphytis lepidosaphes Compere (Hymenoptera: Aphelinidae)	Parasitoid insect
Aphytis lingnanensis Compere (Hymenoptera: Aphelinidae)	Parasitoid insect
Aphytis maculicornis (Masi) (Hymenoptera: Aphelinidae)	Parasitoid insect
Aphytis melinus DeBach (Hymenoptera: Aphelinidae)	Parasitoid insect

Aphytis mytilaspidis (Le Baron) (Hymenoptera: Aphelinidae)	Parasitoid insect
Aphytis roseni DeBach (Hymenoptera: Aphelinidae)	Parasitoid insect
Aphytis sp. (Hymenoptera: Aphelinidae)	Parasitoid insect
Aphytis yanonensis DeBach & Rosen (Hymenoptera: Aphelinidae)	Parasitoid insect
Apiomerus apicalis Burmeister (Hemiptera: Reduviidae)	Predatory insect
Apiomerus lanipes (F.) (Hemiptera: Reduviidae)	Predatory insect
Apiomerus sp. (Hemiptera: Reduviidae)	Predatory insect
Apion ulicis (Foster) (Coleoptera: Brentidae)	Herbivorous insect
Apoanagyrus diversicornis (Howard) (Hymenoptera: Encyrtidae)	Parasitoid insect
Apoanagyrus sp. (Hymenoptera: Encyrtidae)	Parasitoid insect
Apoanagyrus spp. (Hymenoptera: Encyrtidae)	Parasitoid insects
Apodemus sylvaticus (L.) (Rodentia: Muridae)	Plant damaging rodent
Aprostocetus (Hymenoptera: Eulophidae)	Parasitoid insects
Aprostocetus gala (Walker) (Hymnoptera: Eulophidae)	Parasitoid insect
Aprostocetus haitiensis (Gahan) (Hymnoptera: Eulophidae)	Parasitoid insect
Aprostocetus sp. (Hymenoptera: Eulophidae)	Parasitoid insect
Aprostocetus Westwood (Hymenoptera: Eulophidae)	Parasitoid insects
Arachis (Fabales: Fabaceae)	Plants
Araujia spp. (Gentianales: Apocynaceae)	Plant
Archytas divisa (Walk.) Diptera: Tachinidae)	Parasitoid insect
Archytas marmoratus (Tns.) (Diptera: Tachinidae)	Parasitoid insect
Archytas sp. (Diptera: Tachinidae)	Parasitoid insect
Archytas spp. (Diptera: Tachinidae)	Parasitoid insects
Archytas vernalis Curran (Diptera: Tachinidae)	Parasitoid insect
Arcola malloi Pastrana (Lepidoptera: Pyralidae)	Herbivorous insect
Ardalus scutellatus (How.) (Hymenoptera: Eulophidae)	Parasitoid insect
Argiope trifasciata (Forskal) (Araneae: Araneidae)	Predatory spider
Argyrotaenia franciscana Walsingham (Lepidoptera: Tortricidae)	Herbivorous insect
Argyrotaenia sphaleropa (Meyrick) (Lepidoptera: Tortricidae)	Herbivorous insect
Arilus carinatus (Forster *)* (Hemiptera: Reduviidae)	Predatory insect
Arilus sp. *aff. cristatus* (L.) (Hemiptera: Reduviidae)	Predatory insect
Armillaria mellea (Vahl.) P. Kumn (Agaricales: Physalacriaceae)	Phytopathogenic fungus
Arrhenophagus chionaspidis Auriv. (Hymenoptera: Encyrtidae)	Parasitoid insect
Arthrobotrys (Helotiales: Orbiliaceae)	Arthropod pathogenic fungi
Arthrobotrys irregularis (Matr.) Mekht (Helotiales: Orbiliaceae)	Arthropod pathogenic fungus
Arthrobotrys oligospora Fresen (Helotiales: Orbiliaceae)	Arthropod pathogenic fungus
Arthrobotrys sp. (Helotiales: Orbiliaceae)	Arthropod pathogenic fungus
Arundo donax L. (Poales: Poaceae)	Plant
Asarkina ericetorum (Fabricius) (Diptera: Syrphidae)	Predatory insect
Aschersonia aleyrodis Webber (Sordariomycetes: Clavicipitaceae)	Entomopathogenic fungus
Ascia monuste (L.) (Lepidoptera: Pieridae)	Herbivorous insect
Ascia monuste eubotea (Godart) (Lepidoptera: Pieridae)	Herbivorous insect
Ascia monuste orseis Latrielle (Lepidoptera: Pieridae)	Herbivorous insect
Ascogaster (Hymenoptera: Braconidae)	Parasitoid insects
Ascogaster quadridentata Wesmael (Hymenoptera: Braconidae)	Parasitoid insect
Aspergillus flavus Link (Eurotiales: Trichocomaceae)	Entomopathogenic + antagonistic fungus

Asperillium sp.	Antagonistc fungus
Aspidiella hartii (Ckll.) (Hemiptera: Diaspididae)	Herbivorous insect
Aspidiotiphagus citrinus (Crwf) (Hymenoptera: Aphelinidae)	Parasitoid insect
Aspidiotiphagus sp. (Hymenoptera: Aphelinidae)	Parasitoid insect
Aspidiotus destructor Signoret (Hemiptera: Diaspididae)	Herbivorous insect
Aspidiotus hederae (Vallot) (Hemiptera: Diaspididae)	Herbivorous insect
Aspidiotus perniciosus (Comstock) (Hemiptera: Diaspididae)	Herbivorous insect
Aspidiotus spp. (Hemiptera: Diaspididae)	Herbivorous insects
Aspisoma sp. (Coleoptera: Lampyridae)	Predatory insect
Asterodiaspis quercicola (Bouche) (Hemiptera: Asterolecaniidae)	Herbivorous insect
Asynonychus (= Naupactus) cervinus (Boh.) (Coleoptera: Curculionidae)	Herbivorous insect
Ataenius spp. (Coleoptera: Scarabaeidae)	Herbivorous insects
Athelia rolfsii (Curzi) C.C. Tu & Kimbr. *(=Sclerotium rolfsii)* (Atheliales: Atheliaceae*)*	Phytopathogenic fungus
Athesapeuta cyperi Mshl (Coleoptera: Curculionidae)	Herbivorouss insect
Atheta coriaria Kraatz (Coleoptera: Staphylinidae)	Predatory insect
Atopozelus opsimus Elkins (Hemiptera: Reduviidae)	Predatory insect
Atrachelus cinereus crassicornis (Burmeister) (Hemiptera: Reduviidae)	Predatory insect
Atta insularis (Guérrin-Méneville) (Hymenoptera: Formicidae)	Herbivorous insect
Aulacaspis tubercularis Newstead (Hemiptera: Diaspididae)	Herbivorous insect
Aulacaspis yasumatsui Takagi (Hemiptera: Diaspididae)	Herbivorous insect
Aulacorthum solani (Kaltenbach) (Hemiptera: Aphididae)	Herbivorous insect
Autographa californica multiple nucleopolyhedrovirus (AcMNPV) (Baculoviridae)	Entomopathogenic virus
Avena sativa L. (Poales: Poaceae)	Plant
Averrhoa carambola L. (Oxalidales: Oxalidaceae)	Plant
Avetianella longoi Siscaro (Hymenoptera: Encyrtidae)	Parasitoid insect
Aximopsis sp. (Hymenoptera: Eurytomidae)	Parasitoid insect
Azeta melanea Stoll (Lepidoptera: Noctuidae)	Herbivorous insect
Azolla filiculoides Lamarck (Salviniales: Salviniaceae)	Plant
Azolla sp. (Salviniales: Salviniaceae)	Plant
Azotobacter sp. (Pseudomonadales: Pseudomonadaceae/Azotobacteriaceae)	Entomopathogenic bacterium
Azya luteipes Mulsant (Coleoptera: Coccinellidae)	Predatory insect
Azya orbigera Mulsant (Coleoptera: Coccinellidae)	Predatory insect
Azya trinitatis Marshall (Coleoptera: Coccinellidae)	Predatory insect
Azya sp. (Coleoptera: Coccinellidae)	Predatory insect
B	
Baccha sp. (Diptera: Syrphidae)	Predatory insect
Bacillus (Bacillales: Paenibacillaceae)	Entomopathogenic bacteria
Bacillus (= Paenibacillus) popilliae Dutky (Bacillales: Paenibacillaceae)	Entomopathogenic bacterium
Bacillus amyloliquefaciens Priest et al. (Bacillales: Bacillaceae)	Entomopathogenic bacterium
Bacillus firmus Bredemann & Werner (Bacillales: Bacillaceaea)	Entomopathogenic bacterium
Bacillus lentimorbus Dutky (Bacillales: Bacillaceae)	Entomopathogenic bacterium
Bacillus licheniformis (Weigmann) Chester (Bacillales: Bacillaceae)	Entomopathogenic bacterium
Bacillus megaterium de Bary (Bacillales: Bacillaceae)	Entomopathogenic bacterium
Bacillus methylotrophicus Madhaiyan et al (Bacillales: Bacillaceae)	Entomopathogenic bacterium
Bacillus pumilus Meyer and Gottheil (Bacillales: Bacillaceae)	Entomopathogenic bacterium
Bacillus sp. (Bacillales: Bacillaceae)	Entomopathogenic bacterium

Bacillus sphaericus Meyer and Neide (Bacillales: Bacillaceae)	Entomopathogenic bacterium
Bacillus spp. (Bacillales: Bacillaceae)	Entomopathogenic bacteria
Bacillus subtilis Cohn (Bacillales: Bacillaceae)	Entomopathogenic bacterium
Bacillus thruringiensis var. *aizawai* (Bacillales: Bacillaceae)	Entomopathogenic bacterium
Bacillus thuringiensis (Berliner) (Bacillales: Bacillaceae)	Entomopathogenic bacterium
Bacillus thuringiensis var *kurstaki* (Bacillales: Bacillaceae)	Entomopathogenic bacterium
Bacillus thuringiensis var. *israelensis* (Bacillales: Bacillaceae)	Entomopathogenic bacterium
Bacillus thuringiensis var. *thuringiensis* (Bacillales: Bacillaceae)	Entomopathogenic bacterium
Bactericera cockerelli (Sulc) (Hemiptera: Triozidae)	Herbivorous insect
Bactra (Lepidoptera: Tortricidae)	Herbivorous insects
Bactra spp. (Lepidoptera: Tortricidae)	Herbivorous insects
Bactris gasipaes (Kunth.) (Arecales: Arecaceae)	Plant
Bactrocera carambolae Drew and Hancock (Diptera: Tephritidae)	Herbivorous insect
Bactrocera dorsalis (Hendel) (Diptera: Tephritidae)	Herbivorous insect
Baculovirus (Baculoviridae)	Entomopathogenic viruses
Baculovirus anticarsia (Baculoviridae)	Entomopathogenic virus
Baculovirus Anticarsia gemmatalis (Baculoviridae)	Entomopahtogenic virus
Baculovirus diatraea (Baculoviridae)	Entomopathogenic virus
Baculovirus Helicoverpa armigera (Baculoviridae)	Entomopathogenic virus
Baculovirus phthorimaea (Baculoviridae)	Enthomopathogenic virus
Baculovirus VPN 80 & 82	Entomopathogenic virus
Baltimora recta L. (Asterales: Asteraceae)	Plant
Bambusaspis bambusae (Boisduval) (Hemiptera: Asterolecaniidae)	Herbivorous insect
Banana bunchy top virus (Nanoviridae)	Plant pathogenic virus
Baryscapus (Hymenoptera: Eulophidae)	Parasitoid insects
Baryscapus fennahi (Schauff) (Hymenoptera: Eulophidae)	Parasitoid insect
Bassus sp. (Hymenoptera: Braconidae)	Parasitoid insect
Bassus stigmaterus Holloway (Hymenoptera: Braconidae)	Parasitoid insect
Batkoa (Entomophthorales : Entomophthoraceae)	Entomopathogenic fungi
Beauveria (Hypocreales: Cordycipitaceae)	Entomopathogenic fungi
Beauveria bassiana (Bals.-Criv) Vuill (Hypocreales: Cordycipitaceae)	Entomophatogenic fungus
Beauveria brongniartii (Saccardo) Petch (Hypocreales: Cordycipitaceae)	Entomopathogenic fungus
Beauveria globulifer (Speg.) Pic. (Hypocreales: Cordycipitaceae)	Entomopathogenic fungus
Beauveria sp. (Hypocreales: Cordycipitaceae)	Entomopathogenic fungus
Beauveria spp. (Hypocreales: Cordycipitaceae)	Entomopathogenic fungi
Begomovirus (Geminiviridae)	Phytopathogenic viruses
Belvosia nigrifrons Aldrich (Diptera: Tachinidae)	Parasitoid insect
Bemisia tabaci (Gennadius) (Hemiptera: Aleyrodidae)	Herbivorous insect
Beta vulgaris L. (Caryophyllales: Amaranthaceae)	Plant
Bidens pilosa L. (Asterales:Asteraceae)	Plant
Billaea claripalpis Wulp (Diptera: Tachinidae)	Parasitoid insect
Biomphalaria glabrata (Say) (Planobidae)	Harmful snail
Biosteres compensans (Silvestri) (= Opius compensans Silvestri) (Hymenoptera: Braconidae)	Parasitoid insect
Biosteres formasanus (Fullaway) (= *Opius formosanus* Fullaway) (Hymenoptera: Braconidae)	Parasitoid insect
Biosteres longicaudatus (Ashmead) (= *Opius longicaudatus*) (Hymenoptera: Braconidae)	Parasitoid insect
Biosteres novocaledonicus Fullaway (Hymenoptera: Bracondiae)	Parasitoid insect

Biosteres taiensis Fullaway (Hymenoptera:Bracondiae)	Parasitoid insect
Biosteres tryoni (Cameron) (= *Opius tryoni* Cameron) (Hymenoptera: Braconidae)	Parasitoid insect
Biosteres vandenboschi (Fullaway) (= *Opius vandenboschi* Fullaway) (Hymenoptera: Braconidae)	Parasitoid insect
Bipolaris euphorbiae (Han) Muc sfordhovej & Carvalho (Pleosporales: Pelosporaceae)	Phytopathogenic fungus
Bipolaris oryzae (Breda de Haan) Shoemaker (Pleosporales: Pleosporaceae)	Phytoathogenic fungus
Blaesoxipha aculeata Aldrich (Diptera: Sarcophagidae)	Predatory insect
Blaesoxipha atlanis Aldrich (Diptera: Sarcophagidae)	Predatory insect
Blaesoxipha australis Blanchard (Diptera: Sarcophagidae)	Predatory insect
Blaesoxipha caridei (Brétes) (Diptera: Sarcophagidae)	Predatory insect
Blaesoxipha filipjevi Rhod (Diptera: Sarcophagidae)	Predatory insect
Blaesoxipha hunteri (Hough) (Diptera: Sarcophagidae)	Predatory insect
Blaesoxipha neuquenensis Blanchard (Diptera: Sarcophagidae)	Predatory insect
Blaesoxipha opifera Coquillett (Diptera: Sarcophagidae)	Predatory insect
Blaesoxipha reversa Aldrich (Diptera: Sarcophagidae)	Predatory insect
Bonagota cranaodes (Meyrick) (Lepidoptera: Tortricidae)	Herbivorous insect
Boophilus microplus Lahille (Acarina: Ixodidae)	Harmful tick
Botrytis (Helotiales: Sclerotiniaceae)	Phytopathogenic fungi
Botrytis cinerea Pers. (Helotiales: Sclerotiniaceae)	Phytopathogenic fungus
Botrytis sp. (Helotiales: Sclerotiniaceae)	Phytopathogenic fungus
Brachiaria decumbens Stapt (Poales: Poaceae)	Plant
Brachicantha sp. (Coleoptera; Coccinellidae)	Predatory insect
Brachycoryphus nursei (Cam.) (Hymenoptera: Ichneumonidae)	Parasitoid insect
Brachygastra lecheguana (Latreille) (Hymenoptera: Vespidae)	Predatory insect
Brachymeria (Hymenoptera: Chalcididae)	Parasitoid insects
Brachymeria aff. *compsilurae* (Crawford) (Hymenoptera: Chalcididae)	Parasitoid insect
Brachymeria annulata (Fabricius) (Hymenoptera: Chalcididae)	Parasitoid insect
Brachymeria conica (Ashmead) (Hymenoptera:Chalcididae)	Parasitoid insect
Brachymeria incerta (Cress) (Hymenoptera: Chalcididae)	Parasitoid insect
Brachymeria ovata (Say) (Hymenoptera: Chalcididae)	Parasitoid insect
Brachymeria sp. (Hymenoptera: Chalcididae)	Parasitoid insect
Brachymeria subconica Bouček (Hymenoptera: Chalcididae)	Parasitoid insect
Brachystola magna Girard (Orthoptera: Romaleidae)	Herbivorous insect
Brachystola mexicana Bruner (Orthoptera: Romaleidae)	Herbivorous insect
Brachytarsus sp. (Coleoptera: Anthribidae)	Fungus eating insect
Brachyufens osborni (Dozier) (Hymenoptera: Trichogrammatidae)	Parasitoid insect
Brachyufens sp. (Hymenoptera:Trichogrammatidae)	Parasitoid insect
Bracon cajani Muesebeck (Hymenoptera: Braconidae)	Parasitoid insect
Bracon chinensis (Szepl.) (Hymenoptera: Braconidae)	Parasitoid insect
Bracon chontalensis (Cameron) (Hymenoptera:Braconidae)	Parasitoid insect
Bracon gelechiae Ashmead (Hymenoptera: Braconidae)	Parasitoid insect
Bracon greeni Ashmead (Hymenoptera: Braconidae)	Parasitoid insect
Bracon hebetor Say (Hymenoptera: Braconidae)	Parasitoid insect
Bracon kirkpatricki (Wilkinson) (Hymenoptera: Braconidae)	Parasitoid insect
Bracon mellitor Say (Hymenoptera: Braconidae)	Parasitoid insect
Bracon sp. (Hymenoptera: Braconidae)	Parasitoid insect
Bracon thurberiphagae (Mues.) (Hymenoptera: Braconidae)	Parasitoid insect

Bradyrrhoa gilveolella (Treitschke) (Lepidoptera: Pyralidae)	Herbivorous insect
Bradysia matogrossensis (Lane) (Diptera: Sciaridae)	Herbivorous insect
Bradysia sp. (Diptera: Sciaridae)	Herbivorous insect
Bradysia spp. (Diptera: Sciaridae)	Herbivorous insects
Brassica oleracea L. (Brassicales: Brassicaceae)	Plant
Brassica oleracea L. var capitata (Brassicales: Brassicaceae)	Plant
Brassica oleracea L. var. Botrytis (Brassicales: Brassicaceae)	Plant
Brassica oleracea L. var. Italica (Brassicales: Brassicaceae)	Plant
Brassica spp. (Brassicales: Brassicaceae)	Plants
Brassolis astyra astyra (Godart) (Lepidoptera: Nymphalidae)	Herbivorous insect
Brassolis sophorae (L.) (Lepidoptera: Nymphalidae)	Herbivorous insect
Brassolis sophorae sophorae (L.) (Lepidoptera: Nymphalidae)	Herbivorous insect
Brethesiella abnormicornis (Gir.) (Hymenoptera: Encyrtidae)	Parasitoid insect
Brethesiella cf. abnormicornis (Girault) (Hymenoptera: Encyrtidae)	Parasitoid insect
Brevibacterium (Actiomycetales: Brevibacteriaceae)	Nematopathogenic bacteria
Brevicoryne brassicae (L.) (Hemiptera: Aphididae)	Herbivorous insect
Brevipalpus spp. (Acari: Tenuipalpidae)	Herviborous mites
Brevundimonas vesicularis (Busing) Segers (Caulobacteriales: Caulobacteriaceae)	Entomopathogenic bacterium
Bt talcum	Entomopathogenic bacterium
Bucrates capitatus (De Geer) (Orthoptera: Tettigoniidae)	Herbivorous insect
Buenoa scimitra Bare (Hemiptera: Notonectidae)	Predatory insect
Bufo marinus L. (Anura: Bufonidae)	Predatory amphibian
Bufo sp. (Anura: Bufonidae)	Predatory amphibian
Bufo spp. (Anura: Bufonidae)	Predatory amphibians
*Bulimulus sepulcrali*s (Poey) (Mollusca: Gastropoda: Bulimulidae)	Phytophagous snail
Burkholderia glumae (Kurita and Tabei) Urakami et al. (Burkholdeiales: Burkoholderiaceae)	Phytopathogenic bacterium
Byttneria aculeata Jacquin (Malvales: Malvaceae)	Plant
C	
Cactoblastis cactorum (Berg) (Lepidoptera: Pyralidae)	Herbivorous insect
*Caenorhabditis (*Rhabditidae)	Saprohytic nematodes
Caesalpinia spinosa (Feuillée ex Molina) Kuntze (Fabales: Fabaceae)	Plant
Cajanus cajan (L.) Millspaugh (Fabales: Fabaceae)	Plant
Calendula officinalis L. (Asterales: Asteraceae)	Plant
Cales noacki Howard (Hymenoptera: Aphelinidae)	Parasitoid insect
Caligo memnon C. & R. Felder (Lepidoptera: Nymphalidae)	Herbivorous insect
Calliandra surinamensis Benth (Fabales: Fabaceae)	Plant
Callosobruchus chinensis (L.) (Coleoptera: Bruchidae)	Herbivorous insect
Callosobruchus maculatus (F.) (Coleoptera: Bruchidae)	Herbivorous insect
Calosoma alternans Fabricius (Coleoptera: Carabidae)	Predatory insect
Calosoma granulatum Perty (Coleoptera: Carabidae)	Predatory insect
Calosoma sp. (Coleoptera: Carabidae)	Predatory insect
Calpodes ethlius (Stoll) (Lepidoptera: Hesperiidae)	Herbivorous insect
Camnula pellucida Scudder (Orthoptera: Acrididae)	Herbivorous insect
Campletis chlorideae Uchida (Hymenoptera: Ichneumonidae)	Parasitoid insect
Campoletis spp. (Hymenoptera: Ichneumonidae).	Parasitoid insects

Camponotus senex (Fr. Smith) (Hymenoptera: Formicidae)	Predatory insect
Camponotus sexguttatus (Fabricius) (Hymenoptera: Formicidae)	Predatory insect
Campsomeris servillei (Guérin-Menéville) (Hymenoptera: Scoliidae)	Parasitoid insect
Campsomeris tricincta F. (Hymenoptera: Scoliidae)	Parasitoid insect
Campsomeris trifasciata (F.) (Hymenoptera: Scoliidae)	Parasitoid insect
*Campyloneuropsis infumatus (*Carvalho) (Hemiptera: Miridae)	Predadory insect
Candidatus Liberibacter (Rhizobiales: Rhizobiaceae)	Phytopathogenic bacterium
Candidatus Liberibacter *americanus* (Rhizobiales:Rhizobiaceae)	Phytopathogenic bacterium
Candidatus Liberibacter *asiaticus* (Rhizobiales: Rhizobiaceae)	Phytopathogenic bacterium
Candidatus Liberibacter *solanacearum* (Rhizobiales: Rhizobiaceae)	Phytopathogenic bacterium
Candidatus Liberibacter spp. (Rhizobiales: Rhizobiaceae)	Phytopathogenic bacterium
Capsicum annuum L. (Solanales: Solanaceae)	Plant
Capsicum sp. (Solanales: Solanaceae)	Plant
Carabunia waterstoni Subba Rao (Hymenoptera: Encyrtidae)	Parasitoid insect
Carapa guianensis Aubl.) (Sapindales:Meliaceae)	Plant
Cardiochiles diaphaniae Marsh (Hymenoptera: Braconidae)	Parasitoid insect
Cardiochiles nigriceps Viereck (Hymenoptera: Braconidae)	Parasitoid insect
Carduus spp. (Asterales: Asteraceae)	Plants
Carduus acanthoides L. (Asterales: Asteraceae)	Plant
Carduus thoermeri L. (Asterales: Asteraceae)	Plant
Carica papaya L. (Brassicales: Caricaceae)	Plant
Carinodes sp. (Hymenoptera: Ichneumonidae)	Parasitoid insect
Cassia reticulata Willd. (Fabaceae: Leguminosaea)	Plant
Cassia tora L. (Leguminosaea)	Plant
Castnia daedalus Auctt (Lepidoptera: Castniidae)	Herbivorous insect
Castnia licoides (Boisduval) (Lepidoptera:Castniidae)	Herbivorous insect
Castolus plagiaticollis Stål.(Hemiptera:Reduviidae)	Predatory insect
Castrida alternans granulatum (Perty) (Coleoptera: Carabidae)	Predatory insect
Catana clauseni Chapin (Coleoptera: Coccinellidae)	Predatory insect
Catolaccus grandis (Burks) (Hymenoptera: Pteromalidae)	Parasitoid insect
Cedrela odorata L. (= *Cedrela mexicana* Roem.) (Sapindales:Meliaceae)	Plant
Cedrela spp. (Sapindales:Meliaceae)	Plants
Cellulomonas flavigena (Actinomycetales: Cellulomonadaceae)	Pathogenic bacteria
Cephalonomia stephanoderis Betrem (Hymenoptera: Eulophidae)	Parasitoid insect
Cephalosporium (= Lecanicillium) lecanii Zimm. (Hypocreales: Cordycipitaceae)	Entomopathogenic fungus
Ceraeochrysa cf. *claveri* (Navás) (Neuroptera: Chrysopidae)	Predatory insect
Ceraeochrysa cincta (Schneider) (Neuroptera: Chrysopidae)	Predatory insect
Ceraeochrysa claveri (Navás) (Neuroptera: Chrysopidae)	Predatory insect
Ceraeochrysa cubana (Hagen) (Neuroptera: Chrysopidae)	Predatory insect
Ceraeochrysa sp. (Neuroptera: Chrysopidae)	Predatory insect
Ceraeochrysa valida (Banks) (Neuroptera: Chrysopidae)	Predatory insect
Ceraphron Jurine (Hymenoptera: Ceraphronidae)	Parasitoid insect
Ceratitis capitata (Wied.) (Diptera: Tephritidae)	Herbivorous insect
Ceratocapsus dispersus (Carvallo & Fontes) (Hemiptera: Miridae)	Predatory insect
Ceratocapsus paraguayensis (Carvallo & Fontes) (Hemiptera: Miridae)	Predatory insect
Ceratogramma etiennei Delvare (Hymenoptera: Trichogrammatidae)	Parasitoid insect
Ceratophyllum demersum L. (Ceratophyllales: Ceratophyllaceae)	Plant

*Cerconota anonella (*Sepp.) (Lepidoptera: Oecophoridae)	Herbivorous insect
Cercospora (Capnodiales: Mycosphaerellaceae)	Plantpathogenic fungi
Cercospora piaropi Tharp. (Capnodiales: Mycosphaerellaceae)	Plantpathogenic fungus
Cercosporella ageratinae (nomen nudum) (Capnodiales:Hyphomycetes)	Plantpathogenic fungus
Cereochrysa cincta (Schneider) (Neuroptera: Chrysopidae)	Predatory insect
Cereus jamacaru DC. (Caryophyllales:Cactaceae)	Plant
Ceroplastes cirripediformis Comstock (Hemiptera: Coccidae)	Herbivorous insect
Ceroplastes dugesii Lichtenstein (Hemiptera: Coccidae)	Herbivorous insect
Ceroplastes sinensis (Del Guercio) (Hemiptera: Coccidae)	Herbivorous insect
Chaetanaphothrips brevicaulis Hood (Thysanoptera: Thripidae)	Herbivorous insect
Chaetanaphothrips orchidii (Moulton) (Thysanoptera: Thripidae)	Herbivorous insect
Chaetanaphothrips signipennis (Bagnall) (Thysanoptera: Thripidae)	Herbivorous insect
Cheilomenes sexmaculata (Fabricius) (Coleoptera: Coccinellidae)	Predatory insect
Cheiloneurus sp. (Hymenoptera: Encyrtidae)	Hyperparasitoid insect
Chelonus sp. (Hymenoptera: Braconidae)	Parasitoid insect
Chelonus insularis Cresson (Hymenoptera: Braconidae)	Parasitoid insect
Chenopodium quinoa Willd (Caryophyllales: Amaranthaceae)	Plant
Chilocorus bipustulatus L. (Coleoptera: Coccinellidae)	Predatory insect
Chilocorus bivulnerus (Mulsant) (Coleoptera: Coccinellidae)	Predatory insect
Chilocorus cacti L. (Coleoptera: Coccinellidae)	Predatory insect
Chilocorus cf. *cacti* (L.) (Coleoptera: Coccinellidae)	Predatory insect
Chilocorus nigritus (F.) (Coleoptera: Coccinellidae)	Predatory insect
Chilocorus stigma (Say) (Coleoptera: Coccinellidae)	Predatory insect
Chironomus plumosus L. (Diptera: Chironomidae)	Harmful insect
Chistolia sp. (Hymenoptera: Ichneumonidae)	Parasitoid insect
Chnoodes sp. (Coleoptera: Coccinellidae)	Predatory insect
Chnoodes spp. (Coleoptera: Coccinellidae)	Predatory insects
Chondrilla juncea L (Asteraceae: Cynareae)	Plant
Chromaphis juglandicola (Kaltenbach) (Hemiptera: Aphididae)	Herbivorous insect
Chromolaena odorata (L.) R.M. King & H. Rob. (Asterales: Asteraceae)	Plant
Chrysocerca (Neuroptera: Chrysopidae)	Predatory insects
Chrysocharis caribea Boucek (Hymenoptera: Eulophidae)	Parasitoid insect
Chrysocharis sp. (Hymenoptera: Eulophidae)	Parasitoid insect
Chrysocharis vovones (Walker) (Hymenoptera: Eulophidae)	Parasitoid insect
Chrysodeixis includens (Walker) (Lepidoptera: Noctuidae)	Herbivorous insect
Chrysolina hyperici (Foster) (Coleoptera: Chrysomelidae)	Herbivorous insect
Chrysolina quadrigemina (Suffrian) (Coleoptera: Chrysomelidae)	Herbivorous insect
Chrysomphalus aonidum L. (Hemiptera: Diaspididae)	Herbivorous insect
Chrysomphalus ficus (Ashmead) (Hemiptera: Diaspididae)	Herbivorous insect
Chrysonotomyia diastatae (Howard) (Hymenoptera: Eulophidae)	Parasitoid insect
Chrysopa (Neuroptera: Chrysopidae)	Predatory insects
Chrysopa sp. (Neuroptera: Chrysopidae)	Predatory insect
Chrysopa sp. near Silvana Navas (Neuroptera: Chrysopidae)	Predatory insect
Chrysopa spp. (Neuroptera: Chrysopidae)	Predatory insects
Chrysoperla (Neuroptera: Chrysopdiae)	Predatory insects
Chrysoperla asoralis (Banks) (Neuroptera: Chrysopidae)	Predatory insect
Chrysoperla carnea (Stephens) (Neuroptera: Chrysopidae)	Predatory insect

Chrysoperla comanche (Banks) (Neuroptera: Chrysopidae)	Predatory insect
Chrysoperla externa (Hagen) (Neuroptera: Chrysopidae)	Predatory insect
Chrysoperla rufilabris (Burmeister) (Neuroptera: Chrysopidae)	Predatory insect
Chrysoperla sp. (Neuroptera: Chrysopidae)	Predatory insect
Chrysoperla spp. (Neuroptera: Chrysopidae)	Predatory insects
Chrysopodes (Neuroptera: Chrysopidae)	Predatory insects
Chrysomphalus dictyospermi (Morgan) (Hemiptera: Diaspididae)	Herbivorous insect
Cicindela sp. (Coleoptera: Carabidae)	Predatory insect
Cinara (Hemiptera: Aphididae)	Herbivorous insect
Cinara atlantica (Wilson) (Hemiptera: Aphididae)	Herbivorous insect
Cinara pinivora (Wilson) (Hemiptera: Aphididae)	Herbivorous insect
Cinchona pubescens Vahl (Gentinales: Rubiaceae)	Plant
Cirrospilus sp. (Hymenoptera: Eulophidae)	Parasitoid insect
Cirrospilus Westwood (Hymenoptera: Eulophidae)	Parasitoid insects
Cirrospilus quadristriatus (Subba Rao and Ramamani) (Hymenoptera: Eulophidae)	Parasitoid insect
Cirsium spp. (Asterales: Asteraceae)	Plants
Citrostichus phyllocnistoides (Narayanan) (Hymenoptera: Eulophidae)	Parasitoid insect
Citrus leprosis virus C (Rhabdovirus)	Phytopathogenic virus
Citrus sinensis (L.) Osbeck) (Sapindales: Rutaceae)	Plant
Citrus spp. (Rutaceae)	Plants
Cladis nitidula F. (Coleoptera: Coccinellidae)	Predatory insect
Cladobotryum amazonense Bastos, Evans & Samson (Hypocreales: Hypocreaceae)	Antagonistic fungus
Cladosporium fulvum (Pers.) Link. (Capnodiales: Mycosphaerellaceae)	Phytopathogenic fungus
Cladosporium (Capnodiales: Cladosporiaceae)	Phytopathogenic fungi
Cladosporium echinulatum (Berkeley) G.A. de Vries (Capnodiales: Cladosporiaceae)	Phytopathogenic fungus
Cladosporium sp. (Capnodiales: Mycosphaerellaceae)	Phytopathogenic fungus
Clastoptera sp. (Hemiptera: Cercopidae)	Herbivorous insect
Clemora smithi Arrow (Coleoptera: Melolonthidae)	Herbivorous insect
Cleruchoides noackae Lin & Huber (Hymenoptera: Mymaridae)	Parasitoid insect
Clidemia hirta (L.) D. Don (Myrtales:Melastomataceae)	Plant
Clonostachys (Hypocreales: Bionectriaceae)	Fungal parasitic fungi
Clonostachys rosea (Link) Schroers (Hypocreales: Bionectriaceae)	Fungal parasitic fungus
Closterocerus purpureus (Howard) (Hymenoptera: Eulophidae)	Parasitoid insect
Coccidophilus cariba Gordon (Coleoptera: Coccinellidae)	Predatory insect
Coccidophilus citricola Brethes (Coleoptera: Coccinellidae)	Predatory insect
Coccidophilus sp. (Coleoptera: Coccinellidae)	Predatory insect
Coccidoxenoides peregrinus (Timberlake) (Hymenoptera: Encyrtidae)	Parasitoid insect
Coccidoxenoides perminutus Girault (Hymenoptera:Encyrtidae)	Parasitoid insect
Coccinella septenpunctata (L.) (Coleoptera: Coccinellidae)	Predatory insect
Coccinellina sp. (Coleoptera: Coccinellidae)	Predatory insect
Coccipolipus epilachnae Smiley (Acarina: Podapolipidae)	Parasitic mite
Coccobacillus acridiorum D'Herelle	Entomopathogenic bacterium
Coccobius fulvus (Compere and Annecke) (Hymenoptera: Aphelinidae)	Parasitoid insect
Coccophagoides utilis Doutt (Hymenoptera: Aphelinidae)	Parasitoid insect
Coccophagus aleurodici Gir. (Hymenoptera: Aphelinidae)	Parasitoid insect
Coccophagus basalis Compere (Hymenoptera: Aphelinidae)	Parasitoid insect
Coccophagus caridei (Bréthes) (Hymenoptera: Aphelinidae)	Parasitoid insect

Coccophagus gurneyi (Compere) (Hymenoptera: Aphelinidae)	Parasitoid insect
Coccophagus lycimnia (Walker) (Hymenoptera: Aphelinidae)	Parasitoid insect
Coccophagus pulvinariae Compere (Hymenoptera: Aphelinidae)	Parasitoid insect
Coccophagus rusti Compere (Hymenoptera: Aphelinidae)	Parasitoid insect
Coccophagus sp. (Hymenoptera: Aphelinidae)	Parasitoid insect
Coccus hesperidum (Linnaeus) (Hemiptera: Coccidae)	Herbivorous insect
Coccus mangiferae (Green) (Hemiptera: Coccidae)	Herbivorous insect
Coccus spp. (Hemiptera: Coccidae)	Herbivorous insects
Coccus viridis (Green) (Hemiptera: Coccidae)	Herbivorous insect
*Cocos nucifera (*L.) (Arecales: Arecaceae)	Plant
CodVPN *Copitarsia decolora*	Entomopathogenic virus
Coelophora inaequalis F. (Coleoptera: Coccinellidae)	Predatory insect
Coenosia attenuata Stein (Diptera: Muscidae)	Predatory insect
Coleomegilla cubensis Casey (Coleoptera: Coccinellidae)	Predatory insect
Coleomegilla maculata De Geer (Coleoptera: Coccinellidae)	Predatory insect
Coleomegilla quadrifasciata (Schoenherr) (Coleoptera: Coccinellidae)	Predatory insect
Colias lesbia (F.) (Lepidoptera: Pieridae)	Herbivorous insect
Colias lesbia pyrrhothea (Hubn.) (Lepidoptera: Pieridae)	Herbivorous insect
Colletotrichum (Glomerellales: Glomerellaceae)	Phytopathogenic fungi
Colletotrichum acutatum J.H. Simmonds (Glomerellales: Glomerellaceae)	Phytopathogenic fungus
Colletotrichum gloeosporioides (Penz.) Penz. & Sacc (Phyllachorales: Glomerellaceae)	Phytopathogenic fungus
Collops femoratus Schaeffer (Coleoptera: Melyridae)	Predatory insect
Colocasia esculenta (L.) Schott (Alismatalis: Araceae)	Plant
Comperiella bifasciata Howard (Hymenoptera: Encyrtidae)	Parasitoid insect
Compsilura concinnata (Meigen) (Coleoptera: Coccinellidae)	Parasitoid insect
Compsus viridivittatus (Guérin-Méneville) (Coleoptera: Curculionidae)	Herbivorous insect
Comstockaspis perniciosa Comstock (Hemiptera: Diaspididae)	Herbivorous insect
Condylorrhiza vestigialis (Guenée) (Lepidoptera: Crambidae)	Herbivorous insect
Condylostylus graenicheri (Van Duzee) (Diptera: Dolichopodidae)	Predatory insect
Condylostylus similis (Aldrich) (Diptera: Dolichopodidae)	Predatory insect
Condylostylus sp. (Diptera: Dolichopodidae)	Predatory insect
Coniothyrium minitans Campb. (Pleosporales: Leptosphaeriaceae)	Fungal parasitic fungus
Conomyrma sp. (Hymenoptera: Formicidae)	Predatory insect
Conotrachelus albocinereus Fiedler (Coleoptera: Curculionidae)	Herbivorous insect
Conotrachelus psidii Marshall (Coleoptera: Curculionidae)	Herbivorous insect
Contarinia lycopersici Felt (Diptera: Cecidomyiidae)	Herbivorous insect
Conura (Hymenoptera: Chalcididae)	Parasitoid insects
Conura anullifera (Walker) (Hymenoptera: Chalcididae)	Parasitoid insect
Conura destinata (Walker) (Hymenoptera: Chalcididae)	Parasitoid insect
*Conura fulvovariegata (*Cameron) (Hymenoptera: Chalcididae)	Parasitoid insect
Conura hirtifemora Ashmead (Hymenoptera: Chalcididae)	Parasitoid insect
Conura immaculata (Cresson) (Hymenoptera: Chalcididade)	Parasitoid insect
Conura petioliventris Cameron (Hymenoptera: Chalcididae)	Parasitoid insect
Conura pseudofulvovariegata (Becker) (Hymenoptera: Chalcididae)	Parasitoid insect
Conura pulchripes (Cameron) (Hymenoptera: Chalcididade)	Parasitoid insect
Conura sp. (Hymenoptera: Chalcididae)	Parasitoid insect
Convolvulus arvensis L. (Polemoniales: Convolvulaceae)	Plant

Copestylum isabellina (Williston) (Diptera: Syrphidae)	Predatory insect
Copestylum musicanum (Curran) (Diptera: Syrphidae)	Predatory insect
Copestylum punctiferum (Bigot) (Diptera: Syrphidae)	Predatory insect
Copestylum rurale Curran (Diptera: Syrphidae)	Predatory insect
Copestylum sica (Curran) (Diptera: Syrphidae)	Predatory insect
Copidosoma desantisi Annecke & Mynhardt (Hymenoptera: Encyrtidae)	Parasitoid insect
Copidosoma floridanum (Ashmead) (Hymenoptera: Encyrtidae)	Parasitoid insect
Copidosoma gelechiae Howard (Hymenoptera: Encyrtidae)	Parasitoid insect
Copidosoma koehleri Blanchard (Hymenoptera: Encyrtidae)	Parasitoid insect
Copidosoma sp. (Hymenoptera: Encyrtidae)	Parasitoid insect
Copidosoma truncatellum (Dalman) (Hymenoptera: Encyrtidae)	Parasitoid insect
Copitarsia decolora (Guenée) (Lepidoptera: Noctuidae)	Herbivorous insect
Copitarsia incommode (Walker) (Lepidoptera: Noctuidae)	Herbivorous insect
Copitarsia sp. (Lepidoptera: Noctuidae)	Herbivorous insect
Coptera haywardi Loia. (Hymenoptera: Diapriidae)	Parasitoid insect
Coranus spiniscutus Reuter (Hemiptera: Reduviidae)	Predatory insect
Cordia curassavica (Jacq.) Roens. & Schult (Boraginales: Boraginaceae)	Plant
Cordyceps barberi Giard (Hypocreales: Cordycipitaceae)	Entomopathognic fungus
Cordyceps sobolifera (Hill) (Hypocreales: Cordycipitaceae)	Entomopathogenic fungus
Corynebacterium ammoniagenes (Cooke and Keith) (Actinomycetales: Corynevacteriaceae)	Nematode killing bacterium
Corythucha gossypii (Fabricius) (Hemiptera: Tingidae)	Herbivorous insect
Cosmoclopius sp. (Hemiptera: Reduviidae)	Predatory insect
Cosmoclopius sp. aff. *annulosus* Stål (Hemiptera: Reduviidae)	Predatory insect
Cosmopolites sordidus (Germar) (Coleoptera: Curculionidae)	Herbivorous insect
Cotesia (*Apanteles*) *plutellae* *(*Kurdjumov) (Hymenoptera: Braconidae)	Parasitoid insect
Cotesia (Hymenoptera: Braconidae)	Parasitoid insects
Cotesia flavipes Cameron (Hymenoptera: Braconidae)	Parasitoid insect
Cotesia glomerata L. (Hymenoptera: Braconidae)	Parasitoid insect
Cotesia marginiventris (Cresson) (Hymenoptera: Braconidae)	Parasitoid insect
Cotesia plutellae (Kurdjumov) (Hymenoptera: Braconidae)	Parasitoid insect
Cotesia sesamiae (Cameron) (Hymenoptera: Braconidae)	Parasitoid insect
Cotesia sp. (Hymenoptera: Braconidae)	Parasitoid insect
Cotesia vestalis (*plutellae*) (Haliday) (Hymenoptera: Braconidae)	Parasitoid insect
Crematogaster brevispinosa Mayr (Hymenoptera: Formicidae)	Predatory insect
Crematogaster spp. (Hymenoptera: Formicidae)	Predatory insects
Crematogaster torosa (Mayr) (Hymenoptera: Formicidae)	Predatory insect
Crinipellis perniciosa (Stahel) Aime & Phillips-Mora (Agaricales: Marasmiaceae)	Phytopathogenic fungus
Crocidosema (=Epinotia) aporema (Walsingham) (Lepidoptera: Tortricidae)	Herbivorous insect
Croton trinitatis (Millsp.) (Euphorbiales: Euphorbiaceae)	Plant
Crotophaga ani L. (Cuculiformes: Cuculidae)	Predatory bird
Crotophaga major Gmelin (Cuculiformes: Cuculidae)	Predatory bird
Crypticerya genistae (Hampel) ((Hemiptera: Monophlebidae)	Herbivorous insect
Crypticerya genistae Hempel (Hemiptera: Monophlebidae)	Herbivorous insect
Crypticerya multicicatrices Kondo & Unruh (Hemiptera: Monophlebidae)	Herbivorous insect
Cryptoblades gnidiella (Milliére) (Lepidoptera: Pyralidae)	Herbivorous insect
Cryptochaetum iceryae (Willston) (Diptera: Cryptochaetidae)	Parasitoid insect
Cryptognatha affinis (Coleoptera: Coccinellidae)	Predatory insect

Cryptognatha auriculata Muls. (Coleoptera: Coccinellidae)	Predatory insect
Cryptognatha flaviceps (Crotch) (Coleoptera: Coccinellidae)	Predatory insect
Cryptognatha nodiceps Marshall (Coleoptera: Coccinellidae)	Predatory insect
Cryptognatha simillima Sic. (Coleoptera: Coccinellidae)	Predatory insect
Cryptolaemus affinis Crotch (Coleoptera: Coccinellidae)	Predatory insect
Cryptolaemus montrouzieri Mulsant (Coleoptera: Coccinellidae)	Predatory insect
Cryptosporiopsis perennans (Zeller & Childs) Wollenw (Helotiales: Dermateaceae)	Phytopathogenic fungus
Crysso sp. (Araneae: Araneidae)	Predatory spider
Ctenarytaina eucalypti (Maskell) (Hemiptera: Psyllidae)	Herbivorous insect
Ctenarytaina spatulata Taylor (Hemiptera: Psyllidae)	Herbivorous insect
Ctenopharyngodon idella (Valenciennes) (Cypriniformes: Cyprinidae)	Herbivorous fish
Cucurbita moschata Duch. ex Poir. (Cucurbitales: Cucurbitaceae)	Plant
Cucurbita spp. (Cucurbitales: Cucurbitaceae)	Plants
Culex quinquefasciatus Say (Diptera: Culicidae)	Harmful insect
Culex spp. (Diptera: Culicidae)	Harmful insect
Cupressus lusitanica Mill. (Pinales: Cupressaceae)	Plant
Curinus colombianus Chapin (Coleoptera: Coccinellidae)	Predatory insect
Cuscuta americana L. (Solanales: Convolvulaceae)	Plant
Cuscuta indecora Choisy (Solanales: Convolvulaceae)	Plant
Cuscuta spp. (Solanales: Convolvulaceae)	Plants
Cybocephalus nipponicus Endrody-Younga (Coleoptera: Cybocephalidae)	Predatory insect
Cybocephalus sp. (Coleoptera: Cybocephalidae)	Predatory insect
Cycas revoluta Thun. (Cycadales: Cycadaceae)	Plant
Cyclocephala spp. (Coleoptera: Scarabaeidae)	Herbivorous insects
Cycloneda conjugata (Mulsant) (Coleoptera: Coccinellidae)	Predatory insect
Cycloneda oculata (Thumberg) (Coleoptera: Coccinellidae)	Predatory insect
Cycloneda sanguinea (L.) (Coleoptera: Coccinellidae)	Predatory insect
Cycloneda sanguinea limbifer (Casey) (Coleoptera: Coccinellidae)	Predatory insect
Cydia molesta (Busck) (Lepidoptera: Torticidae)	Herbivorous insect
Cydia pomonela granulovirus	Entomopathogenic virus
Cydia pomonela L. (Lepidoptera: Tortricidae)	Herbivorous insect
Cydia sp. (Lepidoptera: Tortricidae)	Herbivorous insect
Cylas formicarius elegantulus (Summers) (Coleoptera: Brentidae)	Herbivorous insect
Cylas formicarius Fabricius (Coleoptera: Brentidae)	Herbivorous insect
Cynara spp. (Asterales: Asteraceae)	Plants
Cynodon dactilon (L.) Pers. (Poales: Poaceae)	Plant
Cyparissius daedalus Cramer (Lepidoptera: Castniidae)	Herbivorous insect
Cyperus rotundus L. (Poales:Cyperaceae)	Plant
Cyrtobagous salviniae Calder and Sands (Coleoptera: Curculionidae)	Herbivorous insect
Cyrtomenus bergi Froeschner (Hemiptera: Cydnidae)	Herbivorous insect
Cyrtopeltis sp. (Hemiptera: Miridae)	Predatory insect
Cyrtorhinus fulvus Knight (Hemiptera: Miridae)	Predatory insect
Cystiphora schmidti (Rübsaamen) (Rubs.) (Diptera: Cecidomyiidae)	Herbivorous insect
D	
Dacnusa sibirica Telenga (Hymenoptera: Braconidae)	Parasitoid insect
Dacnusa sp. (Hymenoptera: Braconidae)	Parasitoid insect

Dactilopius opuntiae (Cocherell) (Hemiptera: Dactylopiidae)	Herbivorous insect
Dactylella sp. (Heloliales: Orbiliaceae)	Nematophagous fungus
Dactylopius austrinus De Lotto (Hemiptera: Dactylopiidae)	Herbivorous insect
Dactylopius sp. nr. *confusus* (Ckll.) (Hemiptera: Dactylopiidae)	Herbivorous insect
Dactylopius opuntiae (Ckll.) (Hemiptera: Dactylopiidae)	Herbivorous insect
Dactylopius spp. (Hemiptera: Dactylopiidae)	Herbivorous insects
Dactylosternum abdominale (Fabricius) (Coleoptera: Hydrophilidae)	Herbivorous insect
Dactylosternum hydrophiloides (MacLeay) (Coleoptera: Hydrophilidae)	Herbivorous insect
Dactylosternum sp. (Coleoptera: Hydrophylidae)	Herbivorous insect
Dactylosternum subdepressum Lap. (Coleoptera: Hydophilidae)	Herbivorous insect
Dalaca noctuides Pfitzner (Lepidoptera: Hepialidae)	Herbivorous insect
Dalaca pallens (Blanchard) (Lepidoptera: Hepialidae)	Herbivorous insect
Dalbulus maidis (De Long & Wolcott) (Hemiptera: Cicadellidae)	Herbivorous insect
Dasiops inedulis Steyskal (Diptera: Lonchaeidae)	Herbivorous insect
Dasiops spp. (Diptera: Lonchaeidae)	Herbivorous insects
Davara caricae Dyar (Lepidoptera: Pyralidae)	Herbivorous insect
Deladenus (= *Beddingia*) *siricidicola* Bedding (Tylenchida: Neotylenchidae)	Entomopatogenic nematode
Deleboea sp. (Hymenoptera: Ichneumonidae)	Parasitoid insect
Delphastus (Coleoptera: Coccinellidae)	Predatory insect
Delphastus argentinicus Nunenmacher (Coleoptera: Coccinellidae)	Predatory insect
Delphastus catalinae Horn (Coleoptera: Coccinellidae)	Predatory insect
Delphastus pallidus Le Conte (Coleoptera: Coccinellidae)	Predatory insect
Delphastus pusillus (LeConte) (Coleoptera: Coccinellidae)	Predatory insect
Delphastus quinculus Gordon (Coleoptera: Coccinellidae)	Predatory insect
Delphastus sp. (Coleoptera: Coccinellidae)	Predatory insect
Delphax maidis Ashmead (Hemiptera: Delphacidae)	Herbivorous insect
Demostipa sp. (Coleoptera: Chrysomelidae)	Herbivorous insect
Deois flavopicta (Stal) (Hemiptera: Cercopidae)	Herbivorous insect
Deroceras reticulatum (Müller) (Pulmonata: Stylommatophora)	Phytophagous slug
Derostenus sp. (Hymenoptera: Eulophidae)	Parasitoid insect
Descampsina sesamiae Mesnil (Diptera: Tachinidae)	Parasitoid insect
Diabrotica balteata LeConte (Coleoptera: Chrysomelidae)	Herbivorous insect
Diabrotica speciosa Germar (Coleoptera: Chrysomelidae)	Herbivorous insect
Diachasmimorpha longicaudata (Ashmead) (Hymenoptera: Braconidae)	Parasitoid insect
Diachasmimorpha tryoni (Cameron) (Hymenoptera: Braconidae)	Parasitoid insect
Diadegma eucerophaga Horstmann (Hymenoptera: Ichneumonidae)	Parasitoid insect
Diadegma insulare (Cresson) (Hymenoptera: Ichneumonidae)	Parasitoid insect
Diadegma molesta Tschek (Hymenoptera: Ichneumonidae)	Parasitoid insect
Diadegma pierisae (Rao) (Hymenoptera: Ichneumonidae)	Parasitoid insect
Diadegma semiclausum Hellen (Hymenoptera: Ichneumonidae)	Parasitoid insect
Diadegma sp. (Hymenoptera: Ichneumonidae)	Parasitoid insect
Diadegma varuna Gupta (Hymenoptera: Ichneumonidae)	Parasitoid insect
Diadromus collaris (Grav.) (Hymenoptera:Ichneumonidae)	Parasitoid insect
Diaeretiella rapae McIntosh (Hymenoptera: Braconidae)	Parasitoid insect
Dialeurodes citrifolii (Morgan) (Hemiptera: Aleyrodidae)	Herbivorous insect
Diaphania hyalinata L. (Lepidoptera: Crambidae)	Herbivorous insect
Diaphania nitidalis (Stoll in Cramer & Stoll) (Lepidoptera: Crambidae)	Herbivorous insect

Diaphania spp. (Lepidoptera: Crambidae)	Herbivorous insects
Diaphorina citri Kuwayama (Hemiptera: Psyllidae)	Herbivorous insect
*Diaprepes (*Coleoptera: Curculionidae)	Herbivorous insects
Diaprepes abbreviatus Linnaeus (Coleoptera: Curculionidae)	Herbivorous insect
Diaprepes spp. (Coleoptera: Curculionidae)	Herbivorous insects
Diachasmimorpha crawfordi (Viereck) (Hymenoptera: Braconidae)	Parasitoid insect
Diaspidiotus perniciosus (Comstock) (Hemiptera: Diaspididae)	Herbivorous insect
Diaspis boisduvalii Signoret (Hemiptera: Diaspididae)	Herbivorous insect
Diatraea andina Box (Lepidoptera: Crambidae)	Herbivorous insect
Diatraea busckella Dyar & Heinrich (Lepidoptera: Crambidae)	Herbivorous insect
Diatraea centrella (Möschl.) (Lepidoptera: Crambidae)	Herbivorous insect
Diatraea guatemalella Schaus (Lepidoptera: Crambidae)	Herbivorous insect
Diatraea impersonatella (Walker) (Lepidoptera: Crambidae)	Herbivorous insect
Diatraea indigenella Dyar & Heinrich (Lepidoptera: Crambidae)	Herbivorous insect
Diatraea lineolata Walker (Lepidoptera: Crambidae)	Herbivorous insect
Diatraea rosa Heinrich (Lepidoptera: Crambidae)	Herbivorous insect
Diatraea rufescens Box (Lepidoptera: Crambidae)	Herbivorous insect
Diatraea saccharalis (F.) (Lepidotpera: Crambidae)	Herbivorous insect
Diatraea sp. (Lepidoptera: Crambidae)	Herbivorous insect
Diatraea spp. (Lepidoptera: Crambidae)	Herbivorous insects
Diatraea tabernella Dyar (Lepidoptera: Crambidae)	Herbivorous insect
Diaulinopsis callichroma Crawford (Hymenoptera: Eulophidae)	Parasitoid insect
Dichelops sp. (Hemiptera: Pentatomidae)	Herbivorous insect
Dichroplus elongatus Giclio-Tos (Orthoptera: Acrididae)	Herbivorous insect
Dichroplus maculipennis (Blanchard) (Orthoptera: Acrididae)	Herbivorous insect
Dichroplus pratensis Bruner (Orhtoptera: Acrididae)	Herbivorous insect
Dichroplus vittatus Bruner (Orthoptera: Acrididae)	Herbivorous insect
Dicyma pulvinata (Berk & M.A. Curtis) Arx (Xylariales: Xylariaceae)	Antagonistic fungus
Digitaria riantha Steud. (= *D. decumbens*) (Poales: Poaceae)	Plant
Diglyphus begini (Ashmead) (Hymenoptera: Eulophidae)	Parasitoid insect
Diglyphus isaea (Walker) (Hymenoptera: Eulophidae)	Parasitoid insect
Diglyphus minoeus (Wlk.) (Hymenoptera: Eulophidae)	Parasitoid insect
Diglyphus sp. (Hymenoptera: Eulophidae)	Parasitoid insect
Diglyphus spp. (Hymenoptera: Eulophidae)	Parasitoid insects
Diglyphus websteri Crawford (Hymenoptera: Eulophidae)	Parasitoid insect
DijnVPN *Dione juno*	Entomopatogenic virus
Dinarmus basalis (Rondani) (Hymenoptera: Pteromalidae)	Parasitoid insect
*Dinarmus vagabundus (*Timb.) (Hymenoptera: Pteromalidae)	Parasitoid insect
Dinoderus minutus(F.) (Coleoptera: Bostrichidae)	Herbivorous insect
Diomus seminulus (Mulsant) (Coleoptera: Coccinellidae)	Predatory insect
Diomus sp. (Coleoptera: Coccinellidae)	Predatory insect
Diomus sp. aff. *Tantillus* (Coleoptera: Coccinellidae)	Predatory insect
Dione juno (Cramer) (Lepidoptera: Nymphalidae)	Herbivorous insect
Dione juno juno (Cramer) (Lepidoptera: Nymphalidae)	Herbivorous insect
Diplazon laetatorius (Fabricius) (Hymenoptera: Ichneumonidae)	Parasitoid insect
Dirhinus giffardii (Silvestri) (Hymenoptera: Chalcididae)	Parasitoid insect
Dissomphalus spp. (Hymenoptera: Bethylidae)	Parasitoid insects

Disorygma pacifica (Yoshimoto) (Hymenoptera: Eucoliidae)	Parasitoid insect
Diuraphis noxia (Mordvilko) (Hemiptera: Aphididae)	Herbivorous insect
Doassansia eichhorniae Ciferri (Doassansiales: Doassansiaceae)	Phytopathogenic fungus
Dolichogenidea sp. (Hymenoptera: Braconidae)	Parasitoid insect
Dolichostoma sp. (Diptera: Tachinidae)	Parasitoid insect
Doru lineare (Eschsholtz) (Dermaptera: Forficulidae)	Predatory insect
Doru luteipes (Scudder) (Dermaptera: Forficulidae)	Predatory insect
Doru sp. (Dermaptera: Forficulidae)	Predatory insect
Doryctes parvus Mues. (Hymenoptera:Braconidae)	Parasitoid insect
Doryctobracon areolatus Szepligeti (Hymenoptera: Braconidae)	Parasitoid insect
Doryctobracon cereus (Gahan) (Hymenoptera: Braconidae)	Parasitoid insect
Doryctobracon crawfordi (Vier.) (Hymenoptera: Braconidae)	Parasitoid insect
Doryctobracon sp. (Hymenoptera: Braconidae)	Parasitoid insect
Doryctobracon trinidadensis (Gah.)(Hymenoptera: Braconidae)	Parasitoid insect
Drino inhertis (Wied.) (Diptera: Tachinidae)	Parasitoid insect
Drino sp. (Diptera: Tachinidae)	Parasitoid insect
Drosophila suzukii (Matsumura) (Diptera: Drosophilidae)	Herbivorous insect
Dusona sp. (Hymenoptera:Ichneumonidae)	Parasitoid insect
Dysdercus andreae (L.) (Hemiptera: Pyrrhocoridae)	Herbivorous insect
Dysdercus discolor Walker (Hemiptera: Pyrrhocoridae)	Herbivorous insect
Dysdercus fulvoniger Deg. subsp. *discolor* Wlk. (Hemiptera: Pyrrhocoridae)	Herbivorous insect
Dysdercus spp. (Hemiptera: Pyrrhocoridae)	Herbivorous insects
Dysmicoccus boninsis (Kuwana) (Hemiptera: Pseudococcidae)	Herbivorous insect
Dysmicoccus brevipes Cockerell (Hemiptera: Pseudococcidae)	Herbivorous insect
Dysmicoccus cryptus Williams = *bispinosus* Beardsley (Hemiptera: Psuedococcidae)	Herbivorous insect
E	
Eacles imperialis magnifica (Walker) (Lepidoptera: Saturniidae)	Herbivorous insect
Ecdytolopha (= *Gymnandrosoma*) *torticornis* (Lepidoptera: Tortricidae)	Herbivorous insect
Ecphoropsis perdistinctus Viereck (Hymenoptera: Ichneumonidae)	Parasitoid insect
Edessa sp. (Hemiptera: Pentatomidae)	Herbivorous insect
Eichhornia crassipes (Mart.) Solms-Laubac (Commelinales: Pontederiaceae)	Plant
Eiphosoma dentator (Fabricius) (Hymenoptera: Ichneumonidae)	Parasitoid insect
Eiphosoma sp. (Hymenoptera: Ichneumonidae)	Parasitoid insect
Eiphosoma spp. (Hymenoptera: Ichneumonidae)	Parasitoid insects
Elachertus sp. (Hymenoptera: Eulophidae)	Parasitoid insect
Elaeidobius kamerunicus Faust (Coleoptera: Curculionidae)	Herbivorous insect
Elaeidobius plagiatus (Fabricius) (Coleoptera: Curculionidae)	Herbivorous insect
Elaeidobius singularis (Faust) (Coleoptera: Curculionidae)	Herbivorous insect
Elasmopalpus lignosellus Zeller (Lepidoptera: Pyralidae)	Herbivorous insect
Elasmopalpus sp. (Lepidoptera: Pyralidae)	Herbivorous insect
Elasmus sp. (Hymenoptera: Eulophidae)	Parasitoid insect
Elasmus Westwood (Hymenoptera: Eulophidae)	Parasitoid insect
Empoasca kraemeri Ross and Moore (Hemiptera: Cicadellidae)	Herbivorous insect
Encarsia (Hymenoptera: Aphelinidae)	Parasitoid insect
Encarsia (= Prospaltella) divergens Silvestri (Hymenoptera: Aphelinidae)	Parasitoid insect
Encarsia basicincta (Gahan) (Hymenoptera: Aphelinidae)	Parasitoid insect

Encarsia berlesei Howard (Hymenoptera Aphelinidae)	Parasitoid insect
Encarsia bimaculata (Heraty and Polaszek) (Hymenoptera: Aphelinidae)	Parasitoid insect
Encarsia citrella (Howard) (Hymenoptera: Aphelinidae)	Parasitoid insect
Encarsia cubensis Gahan (Hymenoptera: Aphelinidae)	Parasitoid insect
Encarsia clypealis (Silvestri) (Hymenoptera: Aphelinidae)	Parasitoid insect
Encarsia dispersa Polaszek (Hymenoptera: Aphelinidae)	Parasitoid insect
Encarsia formosa Gahan (Hymenoptera: Aphelinidae)	Parasitoid insect
Encarsia guadeloupae Viggiani (Hymenoptera: Aphelinidae)	Parasitoid insect
Encarsia hispida De Santis (Hymenoptera: Aphelinidae)	Parasitoid insect
Encarsia longitarsis Myartseva (Hymenoptera: Aphelinidae)	Parasitoid insect
Encarsia lounsburyi (Berlèse & Paoli) (Hymenoptera: Aphelinidae)	Parasitoid insect
Encarsia luteola (Howard) (Hymenoptera: Aphelinidae)	Parasitoid insect
Encarsia meritoria Gahan (Hymenoptera: Aphelinidae)	Parasitoid insect
Encarsia nigricephala Dozier (Hymenoptera: Aphelinidae)	Parasitoid insect
Encarsia noyesi (Hayat) (Hymenoptera: Aphelinidae)	Parasitoid insect
Encarsia opulenta Silvestri (Hymenoptera: Aphelinidae)	Parasitoid insect
Encarsia pergandiella Howard (Hymenoptera: Aphelinidae)	Parasitoid insect
Encarsia perniciosi Tower (Hymenoptera: Aphelinidae)	Parasitoid insect
Encarsia perplexa Huang & Polaszek (Hymenoptera: Aphelinidae)	Parasitoid insect
Encarsia porteri (Mercet (Hymenoptera: Aphelinidae)	Parasitoid insect
Encarsia protransvena Viggiani (Hymenoptera: Aphelinidae)	Parasitoid insect
Encarsia quaintancei Howard (Hymenoptera: Aphelinidae)	Parasitoid insect
Encarsia smithi (Silvestri) (Hymenoptera: Aphelinidae)	Parasitoid insect
Encarsia sophia (Girault & Dodd) (=*E. transvena*) (Hymenoptera: Aphelinidae)	Parasitoid insect
Encarsia sp. (Hymenoptera: Aphelinidae)	Parasitoid insect
Encarsia sp. *(parvella* group) (Hymenoptera: Aphelinidae)	Parasitoid insect
Encarsia sp. nr. *pergandiella* Howard (Hymenoptera: Aphelinidae)	Parasitoid insect
Encarsia sp. nr. *variegata* How (Hymenoptera: Aphelinidae)	Parasitoid insect
Encarsia spp. (Hymenoptera: Aphelinidae)	Parasitoid insects
Encarsia tabacivora (= pergandiella) Viggiani (Hymenoptera: Aphelinidae)	Parasitoid insect
Encarsia telemachusi Evans (Hymenoptera: Aphelinidae)	Parasitoid insect
Encarsiella (Hymenoptera: Aphelinidae)	Parasitoid insects
Encarsiella aleurodici (Girault) (Hymenoptera: Aphelinidae)	Parasitoid insect
Encarsiella new sp. (Hymenoptera: Aphelinidae)	Parasitoid insect
Encarsiella noyesi (Hayat) (Hymenoptera: Aphelinidae)	Parasitoid insect
Encarsiella sp. D (Hymenoptera: Aphelinidae)	Parasitoid insect
Encyrtus infelix (Embleton) (Hymenoptera: Encyrtidae)	Parasitoid insect
Encyrtus lecaniorum (Mayr) (Hymenoptera: Encyrtidae)	Parasitoid insect
Engytatus modestus (Distant) (Hemiptera: Miridae)	Predatory insect
Engytatus varians (Distant) (Hemiptera: Miridae)	Predadory insect
Enicospilus americanus (Christ) (Hymenoptera: Ichneumonidae)	Parasitoid insect
Entomophthora (Entomophthorales: Entomophthoracea)	Entomopathogenic fungi
Entomophthora parvispora MacLeod & Carl (Entomophthorales: Entomophthoraceae)	Entomopathogenic fungus
Entomophthora spp. (Entomophthorales: Entomophthoraceae)	Entomopathogenic fungi
Entomophthora virulenta I.M. Hall & P.H. Dunn (Entomophthorales: Entomophthoraceae)	Entomopathogenic fungus
Entomophthorales sp.	Entomopathogenic fungus
Eodiatraea centrella Mosch (Lepidoptera: Crambidae)	Herbivorous insect

Eodiatraea sp. (Lepidoptera: Crambidae)	Herbivorous insect
Eoreuma morbidella (Dyar) (Lepidoptera: Crambidae)	Herbivorous insect
Ephedrus plagiator (Nees) (Hymenoptera: Braconidae)	Parasitoid insect
Ephestia elutella (Hübner) (Lepidoptera: Pyralidae)	Herbivorous insect
Ephestia kuehniella Zeller (Lepidoptera: Pyralidae)	Herbivorous insect
Epidinocarsis lopezi (De Santis) (Hymenoptera: Encyrtidae)	Parasitoid insect
Epilachna varivestis Mulsant (Coleoptera: Coccinellidae)	Herbivorous insect
Epiphyas postvittana Walker (Lepidoptera: Tortricidae)	Herbivorous insect
Epiplagiops littoralis Blanchard (Diptera: Tachinidae)	Parasitoid insect
Eretmocerus califonicus Howard (Hymenoptera: Aphelinidae)	Parasitoid insect
Eretmocerus eremicus Rose & Zolnerowich (Hymenoptera: Aphelinidae)	Parasitoid insect
Eretmocerus mundus (Mercet) (Hymenoptera: Aphelinidae)	Parasitoid insect
Eretmocerus portoricensis (Dozier) (Hymenoptera:Aphelinidae)	Parasitoid insect
Eretmocerus serius (Silvestri) (Hymenoptera: Aphelinidae)	Parasitoid insect
Eretmocerus sp. (Hymenoptera: Aphelinidae)	Parasitoid insect
Eretmocerus spp. (Hymenoptera: Aphelinidae)	Parasitoid insects
Eretmocerus tejanus Rose & Zolnerow (Hymenoptera: Aphelinidae)	Parasitoid insect
EreVG *Erinnys ello*	Entomopathogenic virus
Eriborus sp. (Hymenoptera: Ichneumonidae)	Parasitoid insect
Erinnyis alope (Drury) (Lepidoptera: Sphingidae)	Herbivorous insect
Erinnyis ello (L.) (Lepidoptera: Sphingidae)	Herbivorous insect
Erinnyis sp. (Lepidoptera: Sphingidae)	Herbivorous insect
Eriophyes sheldoni Ewing (Prostigmata: Eryophidae)	Herbivorous mite
Eriopis chilensis (Hofmann) (Coleoptera: Coccinellidae)	Predatory insect
Eriopis connexa (Germar) (Coleoptera: Coccinellidae)	Predatory insect
Eriopis sp. (Coleoptera: Coccinellidae)	Predatory insect
Eriossoma lanigerum Hausmann (Hemiptera: Aphididae)	Herbivorous insect
Erwinia (Enterobacteriales: Enterobacteriaceae)	Pytopathogenic bacteria
*Erwinia carotovora (*Smith) (Enterobacteriales: Enterobacteriaceae)	Phytopathogenic bacterium
Esclerotinia sp. (Helotiales: Sclerotiniaceae)	Phytopathogenic fungus
Essigella californica (Essig) (Hemiptera: Aphididae)	Herbivorous insect
Etiella zinckenella Treitschke (Lepidoptera: Pyralidae)	Herbivorous insect
Eucalyptus (Myrtales: Myrtaceae)	Plant
Eucalyptus globulus Labill. (Myrtales: Myrtaceae)	Plant
Eucalyptus sp. (Myrtales: Myrtaceae)	Plant
Eucalyptus spp. (Myrtales: Myrtaceae)	Plants
Eucarcelia illota (Curran) (Diptera: Tachinidae)	Parasitoid insect
Eucelatoria armigera (Coquillet) (Diptera: Tachinidae)	Parasitoid insect
Eucelatoria bryani Sabrosky (Diptera: Tachinidae)	Parasitoid insect
Eucelatoria sp. (Diptera: Tachinidae)	Parasitoid insect
Euderus sp. (Hymenoptera: Eulophidae)	Parasitoid insect
Eueupithecia cisplatensis Prout (Lepidoptera: Geometridae)	Herbivorous insect
Euglandina rosea (Ferussac) (Mollusca: Gastropoda: Spiraxidae)	Snail predatory snail
Eulachnus rileyi (Williams) (Hemiptera: Aphididae)	Herbivorous insect
Eunitelus fulvus (Coleoptera: Scarabaeidae)	Herbivorous insect
Eupelmela sp. (Hymenoptera: Eupelmidae)	Parasitoid insect
Eupelmus cushmani (Crawford) (Hymenoptera: Eupelmidae)	Parasitoid insect

EupeVPN *Euprosterna eleasa*	Entomopathogenic virus
Euphorbia heterophylla L. (Malpighiales: Euphorbiaceae)	Plant
Euphorbia pulcherrima Willd (Malpighiales: Euphorbiaceae)	Plant
Euphorocera floridensis Townsend (Diptera: Tachinidae)	Parasitoid insect
Euplectrus comstockii Howard (Hymenoptera: Eulophidae)	Parasitoid insect
Euplectrus platyhypenae Howard (Hymenoptera: Eulophidae)	Parasitoid insect
Euplectrus sp. (Hymenoptera: Eulophidae)	Parasitoid insect
Eupteromalis sp. (Hymenoptera:Pteromalidae)	Parasitoid insect
Eurysacca media Povolný (Lepidoptera: Gelechiidae)	Herbivorous insect
Eurysacca melanocampta Meyrick (Lepidoptera: Gelechiidae)	Hervivorous insect
Eurysacca quinoa Povolny (Lepidoptera: Gelechiidae)	Herbivorous insect
Eurytoma attiva Burks (Hymenoptera: Eurytomidae)	Parasitoid insect
Eurytoma sivinskii Gates & Grissell (Hymenoptera: Eurytomidae)	Parasitoid insect
Euscepes postfasciatus (Fairmaire) (Coleoptera: Curculionidae)	Herbivorous insect
Euschistus heros (Fabricius) (Hemiptera: Pentatomidae)	Herbivorous insect
Euschistus sp. (Hemiptera:Pentatomidae)	Herbivorous insect
Euseius emanus (El-Banhawy) (Acari: Phytoseiidae)	Predatory mite
Euseius (=*Amblyseius*) *victoriensis* Womersly (Acari: Phytoseiidae)	Predatory mite
Euseius concordis (Chant) (Acari: Phytoseiidae)	Predatory mite
Euseius sp. (Acari: Phytoseiidae)	Predatory mite
Euseius stipulatus (Athias-Henriot) (Acari: Phytoseiidae)	Predatory mite
Eutetranychus banksi (McGreg.) (Acari: Tetranychidae)	Herbivorous mite
Eutinobothrus brasiliensis (Hambleton) (Coleoptera: Curculionidae)	Herbivorous insect
Euttetrastichus fennahi (= *Tetrastichus fennahi*) Schauff) (Hymenoptera: Eulophidae)	Parasitoid insect
Euwallacea nr. *fonicatus* Eichhoff (Coleoptera: Curculionidae)	Herbivorous insect
Evania laevigata Olivier (= *appendigaster* E. Guerin-Ménéville) (Hymenoptera: Evaniidae)	Parasitoid insect
Evippe sp. (Lepidoptera: Gelechiidae)	Herbivorous insect
Exasticolus fuscicornis (Cameron) (Hymenoptera: Braconidae)	Parasitoid insect
Exochonus bisbinotatus Gorham (Coloeoptera: Coccinellidae)	Predatory insect
Exochomus marginipennis (LeConte) (Coleoptera: Coccinellidae)	Predatory insect
Exochomus sp. (Coleoptera: Coccinellidae)	Predatory insect
Exophthalmus similis (Drury) (Coleoptera: Curculionidae)	Herbivorous insect
Exophthalmus spp. (Coleoptera: Curculionidae)	Herbivorous insects
Exophthalmus vittatus L. (Coleoptera: Curculionidae)	Herbivorous insect
Exoplectra dubia Crotch (Coleoptera: Coccinellidade)	Predatory insect
F	
Faronta albilinea Hbn. (Lepidoptera: Noctuidae)	Herbivorous insect
Feltiella acarisuga (Vallot) (Diptera: Cecidomyiidae)	Predatory insect
Fidobia citri (Nixon) (Hymenoptera:Platygastridae)	Parasitoid insect
Fidobia sp. (Hymenoptera: Platygastridae)	Parasitoid insect
Flaveria bidentis (L.) Kuntze (Asterales: Asteraceae)	Plant
Foeniculum vulgare Mill. (Apiales: Apiaceae)	Plant
Fopius arisanus (Sonan) (Hymenoptera: Braconidae)	Parasitoid insect
Fopius ceratitivorus Wharton (Hymenoptera: Braconidae)	Parasitoid insect
Fopius vandenboschi (Fullaway) (Hymenoptera: Braconidae)	Parasitoid insect
Forficula euricularia L. (Dermaptera: Forficulidae)	Predatory insect

Fornicia sp. (Hymenoptera: Braconidae)	Parasitoid insect
Frankliniella occidentalis (Pergande) (Thysanoptera: Thripidae)	Herbivorous isnect
Frankliniella parvula Hood (Thysanoptera: Thripidae)	Herbivorous insect
Franklinothrips vespiformis (Crawford) (Thysanoptera: Aeolothripidae)	Predatory insect
Fundella pellucens Zeller (Lepidoptera: Pyralidae)	Herbivorous insect
Fundiseius cesi (Muma) (Acari: Phytoseiidae)	Predatory mite
Fusarium (Hypocreales: Nectriaceae)	Phytopathogenic fungi
Fusarium circinatum (Nirenberg & O'Donnell) (Hypocreales: Nectriaceae)	Phytopathogenic fungus
Fusarium coccophilum (Desm.) Wollenw. & Reinking (Hypocreales: Nectriaceae)	Phytopathogenic fungus
Fusarium episphaerea f. sp. *coccophila* Tul. (Hypocreales: Nectriaceae)	Phytopathogenic fungus
Fusarium euwallaceae S. Freeman, Z. Mendel, T. Aoki & O'Donnell (Hypocreales: Nectriaceae)	Phytopathogenic fungus
Fusarium graminearum Schwabe (Hypocreales: Nectriaceae)	Phytopathogenic fungus
Fusarium guttiforme Nirenberg & O Donnell (Hypocreales: Nectriaceae)	Phytopathogenic fungus
Fusarium oxysporum f. sp. *cubense* (E.F. Sm.) (Hypocreales: Nectriaceae)	Phytopathogenic fungus
Fusarium oxysporum f. sp. *dianthi* W.C. Snyder & H.N. Hansen (Hypocreales:Nectriaceae)	Phytopathogenic fungus
Fusarium oxysporum f. sp. *lycopersici* (Saccardo) Snyder & Hansen (Hypocreales: Nectriaceae)	Phytopathogenic fungus
Fusarium oxysporum Schlecht emend. Snyder & Hansen (Hypocreales:Nectriaceae)	Phytopathogenic and antagonistic fungus
Fusarium solani (Mart.) Sacc. (Hypocreales: Nectriaceae)	Phypopathogenic fungus
Fusarium solani f. sp. *glycines* Roy (Hypocreales: Nectriaceae)	Phytopathogenic fungus
Fusarium solani f. sp. *phaseoli* Kendricht & Snyder (Hypocreales: Nectriaceae)	Phytopathogenic fungus
Fusarium sp. (Hypocreales: Nectriaceae)	Phytopathogenic fungus
Fusarium spp. (Hypocreales:Nectriaceae)	Phytopathogenic fungi
G	
Gaeumannomyces graminis (Sacc.) Arx & Oliv. (Magnaporthales: Magnaporthaceae)	Phytopathogenic fungus
Gaeumannomyces sp. (Magnaporthales: Magnaporthaceae)	Phytopathogenic fungus
Gahaniella saissetia Timberlake (Hymenoptera: Encyrtidae)	Parasitoid insect
Galega officinalis L. (Fabales: Fabaceae)	Plant
Galendromus (=Typhlodromus) occidentalis (Nesbitt) (Acari: Phytoseiidae)	Predatory mite
Galendromus helveolus (Chant) (Acari: Phytoseiidae)	Predatory mite
Galeopsomyia fausta La Salle & Peña (Hymenoptera: Eulophidae)	Parasitoid insect
Galleria mellonella L. (Lepidoptera: Pyralidae)	Herbivorous insect
Gambusia affinis Baird & Girard (Cyprinodontiformes: Poeciliidae)	Predatory fish
Ganaspidium utilis Beardsley (Hymenoptera:Figitidae)	Parasitoid insect
Ganaspis sp. (Hymenoptera: Figitidae)	Parasitoid insect
Gasteracanthe cancriformis L. (Araneae: Araneidae)	Predatory spider
Genea (*=Jaynesleskia) jaynesi* (Aldrich) (Diptera: Tachinidae)	Parasitoid insect
Geococcus coffeae Green (Hemiptera: Pseudococcidae)	Herbivorous insect
Geocoris callosullus Berg (Hemiptera: Geocoridae)	Predatory insect
Geocoris punctipes (Say) (Hemiptera: Geocoridae)	Predatory insect
Geocoris sp. (Hemiptera: Geocoridae)	Predatory insect
Geocoris ventralis (Fieber) (Hemiptera: Geocoridae)	Predatory insect
Geoffroea decorticans (Gillies ex Hook. & Arn.)Burkart (Fabales: Fabaceae)	Plant
Glena bisulca Rindge (Lepidoptera: Geometridae)	Herbivorous insect
Gliocadium sp. (Hypocreales: Hypocreaceae)	Antagonistic fungus

Gliocladium (Hypocreales: Hypocreaceae)	Antagonistic fungus
Globodera rostochiensis (Wollenweber) (Nematoda: Heteroderidae)	Herbivorous nematode
Glycaspis brimblecombei Moore (Hemiptera: Psyllidae)	Herbivorous insect
Glycine max L. Merr. (Fabales: Fabaceae)	Plant
Glypta rufiscutellaris Cresson (Hymenoptera: Ichneumonidae)	Parasitoid insect
Glyptapanteles militaris (Walsh) (Hymenoptera: Braconidae)	Parasitoid insect
Glyptapanteles muesebecki (Blanchard) (Hymenoptera: Braconidae)	Parasitoid insect
Glyptapanteles sp. (Hymenoptera: Braconidae)	Parasitoid insect
Glyptapanteles spp. (Hymenoptera: Braconidae)	Parasitoid insects
Goetheana parvipennis (Gahan) (Hymenoptera: Eulophidae)	Parasitoid insect
Gonatocerus sp. (Hymenoptera: Mymaridae)	Parasitoid insect
Gonaxis quadrilateralis Preston (Stylommatophora: Streptaxidae)	Predatory snail
Gonia peruviana Townsend (Diptera:Tachinidae)	Parasitoid insect
Goniophthalmus halli Mesnil (Diptera: Tachinidae)	Parasitoid insect
Goniozius sp. *punctulaticeps* group (Hymenoptera: Bethylidae)	Parasitoid insect
Goniozus legneri (Gordh) (Hymenoptera: Bethylidae)	Parasitoid insect
Goniozus natalensis Gordh (Hymenoptera: Bethylidae)	Parasitoid insect
Gonipterus gibberus Boisduval (Coleoptera: Curculionidae)	Herbivorous insect
Gonipterus Schoenherr (Coleoptera: Curculionidae)	Herbivorous insect
Gonipterus scutellatus Gyllenhal (Coleoptera: Curculionidae)	Herbivorous insect
Gonipterus platensis Mareli (Coleoptera: Curculionidae)	Herbivorous insect
Gonodonta spp. (Lepidoptera: Erebidae)	Herbivorous insects
Gossypium barbadense L. (Malvales: Malvaceae)	Plant
Granulocyte virus	Virus
Graphium sp. (Microascales: Microascaceae)	Phytopathogenic fungus
Grapholita molesta (Brusck) (Lepidoptera: Tortricidae)	Herbivorous insect
Graptocleptes bicolor (Burmeister) (Hemiptera: Reduviidae)	Predatory insect
Gratiana boliviana Spaeth (Coleoptera: Chrysomelidae)	Herbivorous insect
Gratiana spadicea (Klug) (Coleoptera: Chrysomelidae)	Herbivorous insect
Guignardia citricarpa Kiely (Botryosphaeriales:Botryosphaeriaceae)	Phytopathogenic fungus
Gutierrezia spp. (Asterales:Asteraceae)	Plants
Gymnandrosoma aurantianum Todd M. Gilligan and Marc E. Epstein (Lepidoptera: Tortricidae)	Herbivorous insect
Gymnopolybia sp. (Hymenoptera: Vespidae)	Predatory insect
Gynaikothrips ficorum (Marshal) (Thysanoptera: Phlaeothripidae)	Herbivorous insect
Gynaikothrips sp. (Thysanoptera: Phlaeothripidae)	Herbivorous insect
Gynaikothrips uzeli Zimmermann (Thysanoptera: Phlaeothripidae)	Herbivorous insect
Gypsophila (Caryophyllales: Caryophyllaceae)	Plant
Gypsophila paniculata L. (Caryophyllales: Caryophyllaceae)	Plant
Gyranusoidea indica Shafee, Alam & Argarwal (Hymenoptera: Encyrtidae)	Parasitoid insect
Gyranusoidea sp. (Hymenoptera: Encyrtidae)	Parasitoid insect
Gyranusoidea tebygi Noyes (Hymenoptera: Encyrtidae)	Parasitoid insect
Gyron triatomae Msn (Hymenoptera: Scelionidae)	Parasitoid insect
H	
Habrobracon (Hymenoptera: Braconidae)	Parasitoid insect
Habrobracon hebetor Say (Hymenoptera: Braconidae)	Parasitoid insect

Habrobracon spp. (Hymenoptera: Braconidae)	Parasitoid insects
Habrolepis dalmanni (Westwood) (Hymenoptera: Encyrtidae)	Parasitoid insect
Habronathus sp. (Arachnida: Araneae)	Predatory spider
Haematobia irritans (L.) (Diptera: Muscidae)	Harmful insect
Halticoptera circulus (Walker) (Hymenoptera: Peromalidae)	Parasitoid insect
Halticoptera sp. (Hymenoptera: Pteromalidae)	Parasitoid insect
Hambletonia pseudococcinna Comp. (Hymenoptera: Eulophidae)	Parasitoid insect
Haplothrips gowdeyi (Franklin) (Thysanoptera: Phlaeothripidae)	Herbivorous insect
Harmonia (= *Leis*) sp. (Coleoptera: Coccinellidae)	Predatory insect
Harmonia axyridis (Pallas) (Coleoptera: Coccinellidae)	Predatory insect
Harrisia (Caryophyllales: Cactaceae)	Plants
Harrisia bonplandii (Par.) (Caryophyllales: Cactaceae)	Plant
Harrisia martinii (Labour.) Britton (Caryophyllales: Cactaceae)	Plant
Harrisia tortuosus (J. Forbes ex Otto & A. Dietr.)Britton & Rose (Caryophyllales: Cactaceae)	Plant
Hedylepta (= *Omiodes*) *indicata* (Fabricius) (Lepidoptera: Crambidae)	Herbivorous insect
Hedypathes betulinus (Klug) (Coleoptera: Cerambycidae)	Herbivorous insect
Heilipodus ventralis Hustache (Coleoptera: Curculionidae)	Herbivorous insect
Helianthus annus L. (Asterales: Asteraceae)	Plant
Heliconia bihai (L.) L. (Zingiberales: Heliconiaceae)	Plant
Helicosporina veronae Rambeli (Ascomycota)	Nematophagous fungus
Helicoverpa (Lepidotpera: Noctuidae)	Herbivorous insects
Helicoverpa armigera (Hübner) (Lepidoptera: Noctuidae)	Herbivorous insect
Helicoverpa quinoae Pogue and Harp (Lepidoptera: Noctuidae)	Herbivorous insect
Helicoverpa sp. (Lepidoptera: Noctuidae)	Herbivorous insect
Helicoverpa spp. (Lepidoptera: Noctuidae)	Herbivorous insects
Helicoverpa titicacae Hardwick (Lepidoptera: Noctuidae)	Herbivorous insect
Helicoverpa zea (Boddie) (Lepidoptera: Noctuidae)	Herbivorous insect
Heliothis (Lepidoptera: Noctuidae)	Herbivorous insects
Heliothis sp. (Lepidoptera: Noctuidae)	Herbivorous insect
Heliothis spp. (Lepidoptera: Noctuidae)	Herbivorous insects
Heliothis virescens (F.) (Lepidoptera: Noctuidae)	Herbivorous insect
Hellula phidilealis (Walker) (Lepidoptera: Crambidae)	Herbivorous insect
Helminthosporium oryzae Breda de Haan (Pleosporales: Pleosporaceaea)	Phytopathogenic fungus
Helminthosporium sp. (Pleosporaceaea)	Phytopathogenic fungus
Hemerobius tolimensis Banks (Neuroptera: Hemerobiidae)	Predatory insect
Hemileia vastatrix Berk. & Broome (Pucciniales: Pucciniaceae)	Phytopathogenic fungus
Herpestes auropunctatus Hodgson (Carnivora: Herpestidae)	Predatory mammal
Herpetogramma bipunctalis (Fabricius) (Lepidoptera: Crambidae)	Herbivorous insect
Herse cingulata (= *Agrius*) Fabricius (Lepidoptera: Sphingidae)	Herbivorous insect
He*terodera glycines* Ichinohe (Tylenchida: Heteroderidae)	Phytopathogenic nematode
Heteropsylla cubana Crawford (Hemiptera: Psyllidae)	Herbivorous insect
Heterorhabditis (Rhabditida: Heterorhabditidae)	Entomopathogenic nematodes
Heterorhabditis amazonensis Andaló et al. (Rhabditida: Heterorhabditidae)	Entomopathogenic nematode
Heterorhabditis bacteriophora Poinar (Rhabditidae: Heterorhabditidae)	Entomopathogenic nematode
Heterorhabditis bacteriophora Poinar (strain HC1) (Rhabditidae: Heterorhabditidae)	Entomopathogenic nematode
Heterorhabditis baujardi LPP7 (Rhabditida: Heterorhabditidae).	Entomopathogenic nematode

Heterorhabditis indica Poinar, Karunakar & David (Rhabditida: Heterorhabditidae)	Entomopathogenic nematode
Heterorhabditis marelata Liu & Berry (Rhabditida: Heterorhabditidae)	Entomopathogenic nematode
Heterorhabditis sp. (Nematoda: Heterorhabditidae)	Entomopathogenic nematode
Heterorhabditis spp. (Nematoda: Heterorhabditidae)	Entomopathogenic nematodes
Heterospilus annulicornis Muesebeck (Hymenoptera: Braconidae)	Parasitoid insect
Heterospilus coffeicola Schmiedeknecht (Hymenoptera: Braconidae)	Parasitoid insect
Heterospilus gossypii Muesebeck (Hymenoptera: Braconidae)	Parasitoid insect
Heterospilus hambletoni Muesebeck (Hymenoptera: Braconidae)	Parasitoid insect
Heterospilus sp. (Hymenoptera: Braconidae)	Parasitoid insect
Hevea (Malpighiales: Euphorbiaceae)	Plants
Hexacola sp. (Hymenoptera:Cynipidae)	Parasitoid insect
Hexamermis dactylocercus Poinar Jr. and Linares (Nematoda: Mermithidae)	Entomopathogenic nematode
Hibiscus rosa-sinensis L. (Malvales: Malvaceae)	Plant
Hibiscus sabdariffa L. (Malvales: Malvaceae)	Plant
Hibiscus sp. (Malvales: Malvaceae)	Plant
Hippodamia convergens Guérin-Méneville (Coleoptera: Coccinellidae)	Predatory insect
Hippodamia quinquesignata Kirby (Coleoptera: Coccinellidae)	Predatory insect
Hippodamia sp. (Coleoptera: Coccinellidae)	Predatory insect
Hiranetis sp. (Hemiptera: Reduviidae)	Predatory insect
Hirsutella (Hypocreales: Ophiocordycipitaceae)	Entomopathogenic fungi
Hirsutella eleutherathorum (Nees ex Gray) Petch (Hypocreales: Ophiocordycipitaceae)	Entomopathogenic fungus
Hirsutella nodulosa Petch (Hypocreales: Ophiocordycipitaceae)	Entomopathogenic fungus
Hirsutella sp. (Hypocreales: Ophiocordycipitaceae)	Entomopathogenic fungus
Hirsutella spp. (Hypocreales: Ophiocordycipitaceae)	Entomopathogenic fungi
Hirsutella thompsonii Fisher (Hypocreales: Ophiocordycipitaceae)	Entomopathogenic fungus
Hirsutella verticillioides Charles (Hypocreales: Ophiocordycipitaceae)	Entomopathogenic fungus
Hister bruchi Lewis (Coleoptera: Histeridae)	Predatory insect
Hololepta (=Leionota) quadridentata (Olivier) (Coleoptera:Histeridae)	Predatory insect
Homalotylus eytelweinii (Ratzeburg) (Hymenoptera: Encyrtidae)	Parasitoid insect
Hoplognathoca sp. (Hymenoptera: Mutillidae)	Parasitoid insect
Horismenus (Hymenoptera: Eulophidae)	Parasitoid insects
Horismenus crassus Hansson (Hymenoptera: Eulophidae)	Parasitoid insect
Horismenus sp. (Hymenoptera: Eulophidae)	Parasitoid insect
Horismenus sp.nr. *cupreus* (Ashm.) (Hymenoptera: Eulophidae)	Parasitoid insect
Horismenus spp. (Hymenoptera: Eulophidae)	Parasitoid insects
Hyalomyia chilensis Macq (Diptera: Tachinidae)	Parasitoid insect
Hydrellia sp. (Diptera: Ephydridae)	Herbivorous insect
Hylamorpha elegans (Burmeister) (Coleoptera: Scarabaeidae)	Herbivorous insect
Hylesinus oleiperda F. (Coleoptera: Scolytidae)	Herbivorous insect
Hyperaspis distinguenda (Muls.) (Coleoptera: Coccinellidae)	Predatory insect
Hyperaspis donzeli (Muls.) (Coleoptera: Coccinellidae)	Predatory insect
Hyperaspis festiva Muls (Coleoptera: Coccinellidae)	Predatory insect
Hyperaspis jucunda (Muls.) (Coleoptera: Coccinellidae)	Predatory insect
Hyperaspis notata Mulsant (Coleoptera: Coccinellidae)	Predatory insect
Hyperaspis onerata (Mulsant) (Coleoptera: Coccinellidae)	Predatory insect
Hyperaspis sp. (Coleoptera: Coccinellidae)	Predatory insect
Hyperaspis spp. (Coleoptera: Coccinellidae)	Predatory insects

Hyperaspis trilineata Mulsant (Coleoptera: Coccinellidae)	Predatory insect
Hypericum perforatum L. (Malpighiales:Hypericaceae)	Plant
Hypoaspis miles Berlese (Acari: Laelapidae)	Predatory mite
Hypoaspis sp. (Acari: Laelapidae)	Predatory mite
Hypogeococcus festerianus (Lizer & Trelles) (Hemiptera: Pseudococcidae)	Herbivorous insect
Hypogeococcus pungens Granara de Willink (Hemiptera: Pseudococcidae)	Herbivorous insect
Hypomicrogaster hypsipylae De Santis (Hymenoptera:Braconidae)	Parasitoid insect
Hypothenemus hampei (Ferrari) (Coleoptera: Scolytidae)	Herbivorous insect
Hypsipyla grandella (Zeller) (Lepidoptera: Pyralidae)	Herbivorous insect
Hypsonotus sp. (Coleoptera: Curculionidae)	Herbivorous insect
HzSNPV (*H. zea* nucleopolyhedroviruses)	Entomopathogenic virus
I	
Iatrophobia brasiliensis (Rübs) (Diptera: Cecidomyidae)	Herbivorous insect
Ibalia leucospoides (Hochenwarth) (Hymenoptera: Ibaliidae)	Parasitoid insect
Icerya montserratensis Riley and Howard (Hemiptera: Monophelebidae)	Herbivorous insect
Icerya purchasi Maskell (Hemiptera: Monophelebidae)	Herbivorous insect
Icerya sp. (Hemiptera: Monophelebidae)	Herbivorous insect
Ignelater luminosus (Illiger) (Coleoptera: Elateridae)	Predatory insect
Ilex paraguariensis A. St.-Hil (Aquifoliales: Aquifoliaceae)	Plant
Incamya chilensis Aldrich (Diptera: Tachinidae)	Parasitoid insect
Iphiaulax kimballi Kirkland (Hymenoptera: Braconidae)	Parasitoid insect
Iphiaulax grenadensis (Ashm.) (Hymenoptera:Braconidae)	Parasitoid insect
Iphiseiodes zuluagai Denmark & Muma (Acari: Phytoseiidae)	Predatory mite
Iphiseius (=Amblyseius) degenerans (Berlese) (Acari: Phytoseiidae)	Predatory mite
Ipobracon grenadensis Ashmead (Hymenoptera:Braconidae)	Parasitoid insect
Ipobracon puberuloides Myers (Hymenoptera: Braconidae)	Parasitoid insect
Ipomoea (Solanales: Convolvulaceae)	Plants
Ipomoea sp. (Solanales: Convolvulaceae)	Plant
Ipomoea batatas (L.) Lam. (Solanales: Convolvulaceae)	Plant
Ips spp. (Coleoptera: Curculionidae)	Herbivorous insects
Isaria (=*Paecilomyces*) (Hypocreales: Clavicipitaceae)	Entomopathogenic fungi
Isaria farinosa (Holmsk.) Fr. (Hypocreales: Clavicipitaceae)	Entomopathogenic fungus
Isaria fumosorosea Wize (Hypocreales: Clavicipitaceae)	Entomopathogenic fungus
Isaria javanica (Friedrichs & Bally) Samson & Hywel-Jones (Hypocreales: Cordycipitaceae)	Entomopathogenic fungus
Isaria sp. (Hypocreales: Clavicipitaceae)	Entomopathogenic fungus
Isaria spp. (Hypocreales: Clavicipitaceae)	Entomopathogenic fungi
Ischnapsis longirostris (Sign) (Hemiptera:Diaspididae)	Herbivorous insect
Isosomodes sp. (Hymenoptera: Eurytomidae)	Parasitoid insect
Itoplectus narangae Ashm. (Hymenoptera: Ichneumonidae)	Parasitoid insect
J	
Jaynesleskia jaynesi Towsend (Diptera: Tachinidae)	Parasitoid insect
Jocara sp. (Lepidoptera: Pyralidae)	Herbivorous insect
Juglans regia L. (Fagales: Juglandaceae)	Plant

K	
Keiferia lycopersicella (Walsingham) (Lepidoptera: Gelechiidae)	Herbivorous insect
L	
Labidura riparia (Pallas) (Dermaptera: Labiduridae)	Predatory insect
Lactuca sativa L. (Asterales: Asteraceae)	Plant
Lagochirus dezayasi Dillon (Coleoptera: Cerambycidae)	Herbivorous insect
Lamium amplexicaule L. (Lamiales: Lamiaceae)	Plant
Lapaeumides dedalus (Cram.) (Lepidoptera: Castniidae)	Herbivorous insect
Laphygma (= Spodoptera) frugiperda (J.E.Smith) (Lepidoptera: Noctuidae)	Herbivorous insect
Larra americana Saussure (Hymenoptera: Crabronidae)	Parasitod insect
Larra bicolor Fabricius (Hymenoptera: Crabronidae)	Parasitod insect
Larra sp. (Hymenoptera: Crabonidae)	Parasitod insect
Larra transandina Williams (Hymenoptera: Crabronidae)	Parasitoid insect
Lasioseius (Acari: Blattisociidae)	Predatory mite
Lasioseius chaudhrii (Wu & Wang) (Acari: Blattisociidae)	Predatory mite
Lasioseius dominicensis n. sp Abo-Shnaf, Sanchez & De Moraes (Acari: Blattisociidae)	Predatory mite
Lasioseius oryzae n. sp. Abo-Shnaf, Sanchez & De Moraes (Acari: Blattisociidae)	Predatory mite
Lasioseius prorsoperitrematus n. sp. Abo-Shnaf, Sanchez & De Moraes (Acari: Blattisociidae)	Predatory mite
Lasioseius sanchezensis n. sp. Abo-Shnaf, Sanchez & De Moraes (Acari: Blattisociidae)	Predatory mite
Lebia sp. (Coleoptera: Carabidae)	Predatory insect
Lecanicillium (=*Verticillium*) *lecanii* (Zimm.) Zare & Gams. (Hypocreales: Clavicipitaceae)	Entomopathogenic fungus
Lecanicillium (Hypocreales: Clavicipitaceae)	Entomopathogenic fungi
Lecanicillium sp. (Hypocreales: Clavicipitaceae)	Entomopathogenic fungus
Lecanobius (Hymenoptera: Eupelmidae)	Parasitoid insects
Lecanobius utilis Compere (Hymenoptera: Eupelmidae)	Parasitoid insect
Lejops mexicanus (Macquart) (Diptera: Syrphidae)	Predatory insect
Lepidelphax pistiae Remes Lenicor (Hemiptera: Delphacidae)	Herbivorous insect
Lepidosaphes beckii (Newman) (Hemiptera: Diaspididae)	Herbivorous insect
Lepidosaphes gloverii (Packard) (Hemiptera: Diaspididae)	Herbivorous insect
Leptinotarsa undecemlineata Stal (Coleoptera: Chrysomelidae)	Herbivorous insect
Leptocereus quadricostatus (Bello) Britton & rose (Caryophyllales: Cactaceae)	Plant
Leptocybe invasa Fisher & La Salle (Hymenoptera: Eulophidae)	Herbivorous insect
Leptomastidae sp. (Hymenoptera: Encyrtidae)	Parasitoid insect
Leptomastidea abnormis (Girault) (Hymenoptera: Encyrtidae)	Parasitoid insect
Leptomastix dactylopii (Howard) (Hymenoptera: Encyrtidae)	Parasitoid insect
Leptomastix epona (Noyes) (Hymenoptera: Encyrtidae)	Parasitoid insect
Leptopharsa gibbicarina Froeschner (Hemiptera: Tingidae)	Herbivorous insect
Leptopharsa heveae Drake & Poor (Hemiptera: Tingidae)	Herbivorous insect
Leptophobia aripa Boisduval (Lepidoptera: Pieridae)	Herbivorous insect
Leptopilina boulardi Förster (Hymenoptera: Figitidae)	Parasitoid insect
Leskiopalpus diadema Wied. (Diptera: Tachinidae)	Parasitoid insect
Lespesia archippivora (Riley) (Diptera: Tachinidae)	Parasitoid insect
Lespesia n.sp. (Diptera: Tachinidae)	Parasitoid insect
Lespesia sp. (Diptera: Tachinidae)	Parasitoid insect
Lestodiplosis sp. (Diptera: Cecidomyiidae)	Predatory insect

Lestophonus iceryae Williston (Diptera: Cryptochetidae)	Parasitoid insect
Leucania spp. (Lepidoptera: Noctuidae)	Herbivorous insects
Leucania unipuncta Haw. (Lepidoptera: Noctuidae)	Herbivorous insect
Leucochrysa floridana (Banks) (Neuroptera: Chrysopidae)	Predatory insect
Leucopis bella Loew (Diptera: Chamaemyiidae)	Predatory insect
Leucopis obscura Haliday (Diptera: Chamaemyiidae)	Predatory insect
Leucopodella sp. (Diptera: Syrphidae)	Predatory insect
Leucoptera coffeella (Guérin-Meneville) (Lepidoptera: Lyonetidae)	Herbivorous insect
Ligyrus maimon Erichson (Coleoptera: Dynastidae)	Herbivorous insect
Lincus sp. (Hemiptera: Pentatomidae)	Herbivorous insect
Lindorus lophanthae Casey (Coleoptera: Coccinellidae)	Predatory insect
Linepithema humile (Mayr) (Hymenoptera: Formicidae)	Harmful insect
Lioscymnus diversipes Champ. (Coleoptera: Coccinellidae)	Predatory insect
Liothrips mikaniae (Priesn.) (Thysanoptera: Phlaeothripidae)	Herbivorous insect
Liothrips urichi Karny (Thysanoptera: Phlaeothripidae)	Herbivorous insect
Lipaphis erysimi (Kltb.) (Hemiptera: Aphididae)	Herbivorous insect
Lipolexis oregmae (Gahan) (Hymenoptera: Aphidiidae)	Parasitoid insect
Lippia (= Phyla) nodiflora (L.) Michx. (Lamiales: Verbenaceae)	Plant
Liriomyza (Diptera: Agromyzidae)	Herbivorous insects
Liriomyza huidobrensis (Blanch.) (Diptera: Agromyzidae)	Herbivorous insect
Liriomyza sativae Blanchard (Diptera: Agromyzidae)	Herbivorous insect
Liriomyza sp. (Diptera: Agrommyzidae)	Herbivorous insect
Liriomyza spp. (Diptera: Agrommyzidae)	Herbivorous insects
Liriomyza trifolii (Burgess) (Diptera: Agromyzidae)	Herbivorous insect
Lissorhoptrus brevirostris Suffriam (Coleoptera: Curculionidae)	Herbivorous insect
Lissorhoptrus oryzophilus Kuschel (Coleoptera: Curculionidae)	Herbivorous insect
Listroderes costirostris Schoenherr (Coleoptera: Curculionidae)	Herbivorous insect
Litomastix sp. (Hymenoptera: Encyrtidae)	Parasitoid insect
Litomastix sp. nr. *truncatella* (Dalm.) (Hymenoptera: Encyrtidae)	Parasitoid insect
Lixadmontia franki Wood and Cave (Diptera: Tachinidae)	Parasitoid insect
Lixophaga diatraeae (Townsend) (Diptera: Tachinidae)	Parasitoid insect
Lobesia botrana (Denis & Schiffermüller) (Lepidoptera: Tortricidae)	Herbivorous insect
Longiunguis sacchari (Zhntn) (Hemiptera:Aphididae)	Herbivorous insect
Lotis neglecta Muls. (Coleoptera: Coccinellidae)	Predatory insect
Lotis nigerimma Csy (Coleoptera: Coccinellidae)	Predatory insect
Loxotoma elegans Zeller (Lepidoptera: Depressariidae)	Herbivorous insect
Lutzomyia longipalpis (Diptera: Psychodidae)	Harmful insect
Lycopersicum esculentum Mill. (Solanales: Solanaceae)	Plant
Lycosa spp. (Araneae: Lycosidae)	Predatory spiders
Lydella minense (Town.) (Diptera: Tachinidae)	Parasitoid insect
Lydinolydella metalica Townsend (Diptera: Tachinidae)	Parasitoid insect
Lygus hesperus Knigth (Hemiptera: Miridae)	Herbivorous insect
Lymantria dispar asiatica Linnaeus (Lepidoptera: Lymantriidae)	Herbivorous insect
Lysinibacillus sphaericus (Meyer & Neide) (Planococcaceae)	Entomopathogenic bacterium
Lysiphlebus testaceipes (Cresson) (Hymenoptera: Braconidae)	Parasitoid insect
M	

Maconellicoccus hirsutus (Green) (Hemiptera: Pseudococcidae)	Herbivorous insect
Macrocenctrus prolificus (Hymenoptera: Braconidae)	Parasitoid insect
*Macrocentrus ancylivorus (*Rohwer) (Hymenoptera: Braconidae)	Parasitoid insect
Macrocentrus delicatus (Cresson) (Hymenoptera: Braconidae)	Parasitoid insect
Macrocentrus sp. (Hymenoptera: Braconidae)	Parasitoid insect
Macrodactylus pulchripes Blanchard (Coleoptera: Scarabaeidae)	Herbivorous insect
Macrolophus basicornis (Stal) (Hemiptera: Miridae)	Predatory insect
Macrolophus nr *praeclarus* Distant (Hemiptera: Miridae)	Predatory insect
Macrolophus praeclarus (Distant) (Hemiptera: Miridae)	Predatory insect
Macromalon orientale Kerrich (Hymenoptera: Ichneumonidae)	Parasitoid insect
Macrophomina phaseolina (Tassi) (Botryosphaeriales: Botryosphaeriaceae)	Phytopathogenic fungus
Macrosiphum euphorbiae (Thomas) (Hemiptera: Aphididae)	Herbivorous insect
Macrosiphum rosae L. (Hemiptera: Aphididae)	Herbivorous insect
Macrocyclops (Copepoda: Cyclopidae)	Predatory crustaceans
Mahanarva fimbriolata (Stal) (Hemiptera: Cercopidae)	Herbivorous insect
*Mahanarva posticata (*Stal) (Hemiptera: Cercopidae)	Herbivorous insect
Mahanarva spectabilis (Distant) (Hemiptera: Cercopidae)	Herbivorous insect
Malachra spp. (Malvales: Malvaceae)	Plants
Manduca sexta (L.) (Lepidoptera: Sphingidae)	Herbivorous insect
Manduca sexta paphus (Cramer) (Lepidoptera: Sphingidae)	Herbivorous insect
Manduca spp. (Lepidoptera: Sphingidae)	Herbivorous insects
Mangifera indica L. (Sapindales: Anacardiaceae)	Plant
Manihot (Malpighiales: Euphorbiaceae)	Plants
Manihot esculenta Crantz (Malpighiales: Euphorbiaceae)	Plant
Maranta arundinacea L. (Zingiberales: Marantaceae)	Plant
Marisa cornuarietis L (Gastropoda: Ampullariidae)	Predatory snail
Maruca testulalis (Geyer) (Lepidoptera: Crambidae)	Herbivorous insect
Mastrus ridens Horstmann (Hymenoptera: Ichneumonidae)	Parasitoid insect
Mastrus ridibundus (Gravenhorst) (Hymenoptera: Ichneumonidae)	Parasitoid insect
Matricaria chamomilla L. (Asterales: Asteraceae)	Plant
Megacephala carolina (Linnaeus) (Coleoptera: Carabidae)	Predatory insect
Megacephala chilensis (Laporte de Castelnau) (Coleoptera: Carabidae)	Predatory insect
Megacephala sp. (Coleoptera: Carabidae)	Predatory insect
Megalopyge lanata (Stoll.) (Lepidoptera: Megalopygidae)	Herbivorous insect
Megalopygidae *(*Lepidoptera)	Herbivorous insects
Megamelus scutellaris Berg. (Hemiptera: Delphacidae)	Herbivorous insect
Megaphragma sp. (Hymenoptera:Trichogrammatidae)	Parasitoid insect
Megarhyssa nortoni (Cresson) (Hymenoptera: Ichneumonidae)	Parasitoid insect
Megaselia sp. (Diptera: Phoridae)	Parasitoid insect
Melaleucopis simmondsii Sabrosky (Diptera: Chamaemyiidae)	Predatory insect
Melanagromyza cuscutae Hering (Diptera: Agromyzidae)	Herbivorous insect
Melanagromyza eupatoriella Spencer (Diptera: Agromyzidae)	Herbivorous insect
Melanagromyza obtusa (Malloch) (Diptera: Agromyzidae)	Herbivorous insect
Melanagromyza spp. (Diptera: Agromyzidae)	Herbivorous insects
Melanaphis sacchari (Zehntner) (Hemiptera: Aphididae)	Herbivorous insect
Melanoplus differentialis (Thomas) (Orthoptera: Acrididae)	Herbivorous insect
Melanoplus spp. (Orthoptera: Acrididae)	Herbivorous insects

Melanthera aspera L. (Asterales: Asteraceae)	Plant
Melittobia sp. (Hymenoptera: Eulophidae)	Parasitoid insect
Meloidogyne (Tylenchida: Heteroderidae)	Phytopathogenic nematodes
Meloidogyne incognita (Kofoid & White) Chitwood (Tylenchida: Heteroderidae)	Phytopathogenic nematode
Meloidogyne javanica (Treub) Chitwood (Tylenchidae: Heteroderidae)	Phytopathogenic nematode
Meloidogyne spp.(Tylenchidae: Heteroderidae)	Phytopathogenic nematodes
Menochilus sexmaculatus (F.) (Coleoptera: Coccinellidae)	Predatory insect
*Mescinia parvula (*Zeller) (Lepidoptera: Pyralidae)	Herbivorous insect
Mesocyclops (Copepoda: Cyclopidae)	Predatory crustaceans
Mesocyclops longisetus (Thiébaund) (Copepoda: Cyclopidae)	Predatory crustacean
Mesocyclops sp. (Copepoda: Cyclopidae)	Predatory crustacean
Mesograpta basilaris Wiedemann (Diptera: Syrphidae)	Predatory insect
Mesograpta sp. (Diptera: Syrphidae)	Predatory insect
Metacanthus tenellus Stal (Hemiptera: Berytidae)	Predatory insect
Metaleurodicus cardini (Back) (Hemiptera: Aleyrodidae)	Herbivorous insect
Metamasius anceps Gyllenhal (Coleoptera: Curculionidae)	Herbivorous insect
Metamasius callizona (Chevrolat) (Coleoptera: Curculionidae)	Herbivorous insect
Metamasius dimidiatipennis Champeon (Coleoptera: Curculionidae)	Herbivorous insect
Metamasius hemipterus L. (Coleoptera: Curculionidae)	Herbivorous insect
Metamasius quadrilineatus Champion (Coleoptera: Curculionidae)	Herbivorous insect
Metamasius sp. (Coleoptera: Curculionidae)	Herbivorous insect
Metaphycus (Hymenoptera: Encyrtidae)	Parasitoid insects
Metaphycus anneckei Guerrieri & Noyes (Hymenoptera: Encyrtidae)	Parasitoid insect
Metaphycus cereales sp. nov. Myartseva & Ruiz (Hymenoptera:Encyrtidae)	Parasitoid insect
Metaphycus flavus (Howard) (Hymenoptera: Encyrtidae)	Parasitoid insect
Metaphycus helvolus (Compere) (Hymenoptera: Encyrtidae)	Parasitoid insect
Metaphycus lounsburyi (Howard) (Hymenoptera: Encyrtidae)	Parasitoid insect
Metaphycus stanleyi Compere (Hymenoptera: Encyrtidae)	Parasitoid insect
Metarhizium (Hypocreales: Clavicipitaceae)	Entomopathogenic fungi
Metarhizium acridum (Driver & Milner) J.F. Bisch, Rehner & Humber (Hypocreales: Clavicipitaceae)	Entomopathogenic fungus
Metarhizium anisopliae (Metchnikoff) Sorokin (Hypocreales: Clavicipitaceae)	Entomophatogenic fungus
Metarhizium brunneum Petch (Hypocreales: Clavicipitaceae)	Entomopathogenic fungus
Metarhizium sp. (Hypocreales: Clavicipitaceae)	Entomopathogenic fungus
Metarhizium spp. (Hypocreales: Clavicipitaceae)	Entomopathogenic fungi
Meteorus laphygmae Vier. (Hymenoptera: Braconidae)	Parasitoid insect
Meteorus sp. (Hymenoptera:Braconidae)	Parasitoid insect
Metopolophium dirhodum Walker (Hemiptera: Aphididae)	Herbivorous insect
Metrogaleruca obscura De Geer (Coleoptera: Chrysomelidae)	Herbivorous insect
Micranchenus lineola (F) (Hemiptera: Reduviidae)	Predatory insect
Microcera sp. (Hypocreales: Nectriaceae)	Entomopathogenic fungus
Microcharops sp. (Hymenoptera: Ichneumonidae)	Parasitoid insect
Microlarinus lareynii (Duv.) (Coleoptera: Curculionidae)	Herbivorous insect
Microlarinus lypriformis (Woll.) (Coleoptera: Curculionidae)	Herbivorous insect
Microplitis plutellae Muesbeck (Hymenoptera: Braconidae)	Parasitoid insect
Microplitis sp. (Hymenoptera: Braconidae)	Parasitoid insect
Microseromasia sphenophori Vill (Diptera: Tachinidae)	Parasitoid insect

Microsporidian sp. (Opisthosporidia)	Entomopathogenic fungus
Microtus arvalis (Pallas) (Rodentia: Cricetidae)	Harmful mammal
Migdolus fryanus (Westwood) (Coleoptera: Vesperidae)	Herbivorous insect
Mikania micrantha Kunth (Asterales: Asteraceae)	Plant
Miobiopsis diadema (Wiedemann) (Diptera:Tachinidae)	Parasitoid insect
Mirax insularis Mues. (Hymenoptera:Braconidae)	Parasitoid insect
Mocis latipes Guenée (Lepidoptera: Noctuidae)	Herbivorous insect
Mocis spp. (Lepidoptera: Noctuidae)	Herbivorous insects
Momordica charantia L. (Cucurbitales: Cucurbitaceae)	Plant
Moniliasis	Plant
Moniliophthora perniciosa (Stahel) Aime & Phillips-Mora (Agaricales: Marasmiaceae)	Phytopathogenic fungus
Moniliophthora roreri (Cif.) H.C. Evans, Stalpers, Samson & Benny (Agaricales: Marasmiaceae)	Phytopathogenic fungus
Monomorium floricola (Jerdon) (Hymenoptera: Formicidae)	Predatory insect
Mononychellus caribbeanae (McGregor) (Acari: Tetranychidae)	Herbivorous mite
Mononychellus tanajoa (Bondar) (Acari: Tetranychidae)	Herbivorous mite
Montandoniola sp. (Hemiptera: Anthocoridae)	Predatory insect
Montina confusa (Stal) (Hemiptera: Reduviidae)	Predatory insect
Mortierella (Mortierellales: Mortierellaceae)	Nematophagous fungi
Mucor (Mucorales: Mucoraceae)	Phytopathogenic fungi
Murraya J. Koenig ex L. (Sapindales: Rutaceae)	Plants
Murraya paniculata (L.) Jack (Sapindales:Rutaceae)	Plant
Musa sp. (Zingiberales: Musaceae)	Plant
Musa spp. (Zingiberales: Musaceae)	Plants
Musca domestica L. (Diptera: Muscidae)	Harmful insect
Musca spp. (Diptera: Muscidae)	Harmful insects
Muscidifurax raptor (Girault & Sanders) (Hymenoptera: Pteromalidae)	Parasitoid insect
Muscidifurax raptorellus Kogan & Legner (Hymenoptera: Pteromalidae)	Parasitoid insect
Muscidifurax spp. (Hymenoptera: Pteromalidae)	Parasitoid insects
Muscidifurax uniraptor Kogan & Legner (Hymenoptera: Pteromalidae)	Parasitoid insect
Muscidifurax zaraptor Kogan & Legner (Hymenoptera: Pteromalidae)	Parasitoid insect
Mus musculus (L.) (Rodentia: Muridae)	Harmful mammal
Mycorrhiza sp.	Symbiotic fungi
Mycosphaerella fijiensis Morelet (Mycosphaerellales: Mycosphaerellaceae)	Phytopathogenic fungus
Myelobia bimaculata (Box) (Lepidoptera: Crambidae)	Herbivorous insect
Myiopharus doryphorae (Riley) (Diptera: Tachinidae)	Parasitoid insect
Myrothecium roridum Tode ex Fr. (Hypocreales)	Phytopathogenic fungus
Mythimna unipuncta Haworth (Lepidoptera: Noctuidae)	Herbivorous insect
Myzinum ephippium F. (= *M. xanthonotus* (Rohw.) (Hymenoptera: Tiphiidae)	Parasitoid insect
Myzinum haemorrhoidalis F. (Hymenoptera: Tiphiidae)	Parasitoid insect
Myzus (Hemiptera: Aphididae)	Herbivorous insects
Myzus persicae (Sulzer) (Hemiptera: Aphididae)	Herbivorous insect
N	
Nabis capsiformis (Germar) (Hemiptera: Nabidae)	Predatory insect
Nabis sordidus Reuter (Hemiptera: Nabidae)	Predatory insect
Nabis sp. (Hemiptera: Nabidae)	Predatory insect

Nasonia spp. (Hymenoptera: Pteromalidae)	Parasitoid insects
Nasonia vitripennis (Walker) (Hymenoptera: Pteromalidae)	Parasitoid insect
Nassella (= *Stipa*) *trichotoma* Nees (Poales:Poaceae)	Plant
Nassella neesiana Trin. & Rupr. (Poales:Poaceae)	Plant
Nasutitermes ephratae (Holmgr.) (Isoptera: Termitidae)	Harmful insect
Naupactus xanthographus (Germar) (Coleoptera: Curculionidae)	Herbivorous insect
Nausigaster meridionalis Townsend (Diptera: Syrphidae)	Predatory insect
Nemorilla sp. (Diptera: Tachinidae)	Parasitoid insect
Neoaplectana (strain P2M) (Nematoda: Steinernematidae)	Entomophatogenic nematode
Neoaplectana carpocapsae Weiser (= *Steinernema feltidae*) (Nematoda: Steinernematidae)	Entomophatogenic nematode
Neocatolaccus longiventris (Gahan) (Hymenoptera: Pteromalidae)	Parasitoid insect
Neochetina bruchi Hustache (Coleoptera: Curculionidae)	Herbivorous insect
Neochetina eichhorniae Warner (Coleoptera: Curculionidae)	Herbivorous insect
Neochrysocharis sp. (Hymenoptera: Eulophidae)	Parasitoid insect
Neocurtilla hexadactyla (Perty) (Orthoptera: Gryllotalpidae)	Herbivorous insect
Neodiplogrammus quadrivittatus (Olivier) (Coleoptera: Curculionidae)	Herivorous insect
*Neodusmetia sangwani (*Subba Rao) (Hymenoptera: Encyrtidae)	Parasitoid insect
Neohydronomus affinis Hustache (Coleoptera: Curculionidae)	Herbivorous insect
Neoleucinodes elegantalis (Guenée) (Lepidoptera: Crambidae)	Herbivorous insect
Neoseiulus (= Amblyseius) californicus (McGregor) (Acari: Phytoseiidae)	Predatory mite
Neoseiulus (Acari: Phytoseiidae)	Predatory mites
Neoseiulus (Amblyseius) barkeri Hughes (Acari: Phytoseiidae)	Predatory mite
Neoseiulus (Amblyseius) cucumeris (Oudemans) (Acari: Phytoseiidae)	Predatory mite
Neoseiulus anonymus (Chant & Baker) (Acari: Phytoseiidae)	Predatory mite
Neoseiulus baraki Athias-Henriot (Acari: Phytoseiidae)	Predatory mite
Neoseiulus longispinosus (Evans) (Acari: Phytoseiidae)	Predatory mite
Neozygites fresenii (Nowakovski) Remaudière and Keller (Entomophthorales: Neozygitaceae)	Entomopathogenic fungus
Neozygites parvispora Remaudière and Keller (Entomophthorales: Neozygitaceae)	Entomopathogenic fungus
Nephaspis (Coleoptera: Coccinellidae)	predatory ladybeelte
Nephaspis amnicola Wingo (Coleoptera: Coccinellidae)	Predatory insect
Nephaspis bicolor Gordon (Coleoptera: Coccinellidae)	Predatory insect
Nephaspis nigra Gordon (Coleoptera: Coccinellidae)	Predatory insect
Nephaspis sp. (Coleoptera: Coccinellidae)	Predatory insect
Nephus regularis Sicard (Coleoptera: Coccinellidae)	Predatory insect
Nephus sp. (Coleoptera: Coccinellidae)	Predatory insect
Nesidiocoris (= *Cyrtopeltis*) *tenuis* (Reuter) (Hemiptera: Miridae)	Predatory insect
Nezara viridula (Linnaeus) (Hemiptera: Pentatomidae)	Herbivorous insect
Nicotiana glauca Graham (Solanales: Solanaceae)	Plant
Nicotiana tabacum L. (Solanales: Solanaceae)	Plant
Nipaecoccus nipae Mask. (Hemiptera: Pseudococcidae)	Herbivorous insect
Niphograpta albiguttalis (Sameal.) (Lepidoptera: Crambidae)	Herbivorous insect
Nodita sp. (Neuroptera: Chrysopidae)	Predatory insect
Nomurea rileyi (Farlow) Samson (Hypocreales: Clavicipitaceae)	Entomopathogenic fungus
Notiobia laevis bolivianus (Van Emdem) (Coleoptera: Carabidae)	Predatory insect
Notiobia schnusei Emden (Coleoptera: Carabidae)	Predatory insect
Notocyrtus dorsalis (Gray) (Hemiptera: Reduviidae)	Predatory insect

Notozullia entreriana (Berg. (Hemiptera: Cercopidae)	Herbivorous insect
NPV (*Anticarsia gemmatalis* nucleopolyhedrosis virus)	Entomopathogenic virus
Nylanderia fulva (Mayr) (Hymenoptera: Formicidae)	Predatory insect
O	
Ocyptamus (Diptera: Syrphidae)	Predatory insects
Ocyptamus clava (Fabricius) (Diptera: Syrphidae)	Predatory insect
Ocyptamus dimidiatus (Fabricius) (Diptera: Syrphidae)	Predatory insect
Ocyptamus gastrostactus (Wiedemann) (Diptera: Syrphidae)	Predatory insect
Ocyptamus stenogaster (Williston) (Diptera: Syrphidae)	Predatory insect
Odontomachus brunneus (Patton) (Hymenoptera: Formicidae)	Predatory insect
Odontosema anastrephae Borgmeier (Hymenoptera: Figitidae)	Parasitoid insect
Oebalus insulares Stal (Hemiptera: Pentatomidae)	Herbivorous insect
Oebalus mexicana (Sailer) (Hemiptera: Pentatomidae)	Herbivorous insect
Oebalus poecilus (Dallas) (Hemiptera: Pentatomidae)	Herbivorous insect
Oebalus sp. (Hemiptera: Pentatomidae)	Herbivorous insect
Oecanthus sp. (Orthoptera: Gryllidae)	Predatory insect
Oeneis nigrans Muls (Coleoptera: Coccinellidae)	Predatory insect
Oenonogastra microrhopalae Ashmead (Hymenoptera: Braconidae)	Parasitoid insect
Oenonogastra sp. (Hymenoptera: Braconidae)	Parasitoid insect
Oerskovia (Cellulomonadaceae)	Fungal pathogenic bacteria
Oerskovia turbata (Erikson) (Cellulomonadaceae)	Fungal pathogenic bacteria
Oidiopsis taurica (Lév.) E.S. Salmon (Erysiphales: Erysiphaceae)	Phytopathogenic fungus
Oidium sp.	Phytopathogenic fungus
Oiketicus sp. (Lepidoptera: Psychidae)	Herbivorous insect
Oligonychus milleri (McG.) (Acari: Tetranychidae)	Herbivorous mite
Oligosita giraulti Crawford (Hymenoptera: Trichogrammatidae)	Parasitoid insect
Oligota minuta Cameron (Coleoptera: Staphylinidae)	Predatory insect
Oligota sp. (Coleoptera: Staphylinidae)	Predatory insect
Olla v-nigrum (Mulsant) (Coleoptera: Coccinellidae)	Predatory insect
Olygonichus yothersi (McGregor) (Acari: Tetranychidae)	Herbivorous mite
Omiodes indicata (Fabricius) (Lepidoptera: Crambidae)	Herbivorous insect
Oncorhynchus mykiss (Walbaum) (Salmoniformes: Salmonidae)	Fish
Onopordum spp. (Asterales: Asteraceae)	Plants
Ontherus sulcator Fabricius (Coleoptera: Sacarabaeidae)	Herbivorous insect
Onthophagus Taurus (Schreber) (Coleoptera: Scarabaeidae)	Herbivorous insect
Ooencyrtus (Hymenoptera: Encyrtidae)	Parasitoid insects
Ooencyrtus sp. (Hymenoptera: Encyrtidae)	Parasitoid insect
Ooencyrtus submetallicus Howard (Hymenoptera: Encyrtidae)	Parasitoid insect
Ooencyrtus trinidadensis Crawford (Hymenoptera: Encyrtidae)	Parasitoid insect
Oomyzus sokolowskii (Kurdj.) (Hymenoptera: Eulophidae)	Parasitoid insect
Oomyzus sp. (Hymenoptera: Eulophidae)	Parasitoid insect
Ooyncyrtus sp. (Hymenoptera: Encyrtidae)	Parasitoid insect
Ophion spp. (Hymenoptera: Ichneumonidae	Parasitoid insects
Opius crawfordi Viereck (Hmenoptera: Braconidae)	Parasitoid insect
Opius oophilus Fullaway (Hymenoptera: Braconidae)	Parasitoid insect
Opius anastrephae Vier (Hymenoptera: Braconidae)	Parasitoid insect

Opius bellus Gahan (Hymenoptera: Braconidae)	Parasitoid insect
Opius cereus (Gah) (Hymenoptera: Braconidae)	Parasitoid insect
Opius concolor Szépligeti (Hymenoptera: Braconidae)	Parasitoid insect
Opius concolor var. *Siculus Mon.* (Hymenoptera: Braconidae)	Parasitoid insect
Opius dimidiatus Ashmead (Hymenoptera: Braconidae)	Parasitoid insect
Opius dissitus Muesebeck (Hymenoptera:Braconidae)	Parasitoid insect
Opius forticornis Cameron (Hymenoptera: Braconidae)	Parasitoid insect
Opius hirsutus Tobias (Hymenoptera: Braconidae)	Parasitoid insect
Opius incisi Silvestri (Hymenoptera: Braconidae)	Parasitoid insect
Opius longicaudatus (Ash.) (Hymenoptera: Braconidae)	Parasitoid insect
Opius oophilus Fullaway (= *Biosteres arisanus* (Sonan) (Hymenoptera: Braconidae)	Parasitoid insect
Opius sp. (Hymenoptera: Braconidae)	Parasitoid insect
Opius spp. (Hymenoptera: Braconidae)	Parasitoid insects
Opsiphanes cassina C. & R. Felder (Lepidoptera: Nymphalidae)	Herbivorous insect
Opsiphanes invirae Huebner (Lepidoptera: Nymphalidae)	Herbivorous insect
Opuntia (Caryophyllales: Cactaceae)	Plants
Opuntia aurantiaca Lindley (Caryophyllales:Cactaceae)	Plant
Opuntia dillenii (Ker-Gawler) Haworth (Cayophyllales: Cactaceae)	Plant
Opuntia ficus-indica Mill. (Caryophyllales:Cactaceae)	Plant
Opuntia lindheimeri Engelm. (Caryophyllales:Cactaceae)	Plant
Opuntia spp. (Caryophyllales: Cactaceae)	Plants
Opuntia stricta (Haw.) (Caryophyllales:Cactaceae)	Plant
Opuntia triacantha (Willdenow) (Caryophyllales:Cactacea)	Plant
Opuntia tuna (L.) Mill. (Caryophyllales:Cactaceae)	Plant
Opuntia vulgaris Mill. (Caryophyllales:Cactaceae)	Plant
Orgilus lepidus (Muesebeck) (Hymenoptera: Braconidae)	Parasitoid insect
Orgilus obscurator (Nees) (Hymenoptera: Braconidae)	Parasitoid insect
Orgilus sp. (Hymenoptera: Braconidae)	Parasitoid insect
Orius (Hemiptera: Anthocoridae)	Predatory insects
Orius euryale Herring (Hemiptera: Anthocoridae)	Predatory insect
Orius insidiosus (Say) (Hemiptera: Anthocoridae)	Predatory insect
Orius laevigatus (Fieber) (Hemiptera: Anthocoridae)	Predatory insect
Orius pumilio (Champion) (Hemiptera: Anthocoridae)	Predatory insect
Orius sp. (Hemiptera: Anthocoridae)	Predatory insect
Orius spp. (Hemiptera: Anthocoridae)	Predatory insects
Orius tristicolor (White) (Hemiptera: Anthocoridae)	Predatory insect
Ormia depleta (Wiedemann) (Diptera: Tachinidae)	Parasitoid insect
Ormyrus orientalis Walker (Hymenoptera: Ormyridae)	Parasitoid insect
Ornidia major Curran (Diptera: Syrphidae)	Predatory insect
Ornidia obesa F. (Diptera: Syrphidae)	Predatory insect
Orthezia insignis Browne (Hemiptera: Ortheziidae)	Herbivorous insect
Orthezia olivicola Beingolea (Hemiptera: Ortheziidae)	Herbivorous insect
Orthezia praelonga Douglas (Hemiptera: Ortheziidae)	Herbivorous insect
Orthezia spp. (Hemiptera: Orhteziidae)	Herbivorous insects
Orthoderella ornata Giglio-Tos (Mantodea: Mantidae)	Predatory insect
Oryza (Poales: Poaceae)	Plants
Oryza sativa L. (Poales: Poaceae)	Plant

Otiorhynchus sulcatus (Fabricius) (Coleoptera: Curculionidae)	Herbivorous insect
Oxydia sp. (Lepidoptera: Geometridae)	Herbivorous insect
Oxydia trychiata (Guenee) (Lepidoptera: Geometridae)	Herbivorous insect
Oxydia vesulia Cramer (Lepidoptera: Geometridae)	Herbivorous insect
Ouichuana picadoi Knab (Diptera: Syrphidae)	Predatory insect
P	
Pachnaeus citri Marshall (Coleoptera: Curculionidae)	Herbivorous insect
Pachnaeus litus (Germar) (Coleoptera: Curculionidae)	Herbivorous insect
Pachycrepoideus sp. (Hymenoptera: Pteromalidae)	Parasitoid insect
Pachycrepoideus vindemiae Rondani (Hymenoptera: Pteromalidae)	Parasitoid insect
Pachylister chinensis Quensel (Coleoptera: Histeridae)	Predatory insect
Pachyneuron albutium Walker (Hymenoptera: Pteromalidae)	Parasitoid insect
Pachyneuron sp. (Hymenoptera: Pteromalidae)	Parasitoid insect
Pachyneuron (Hymenoptera: Pteromalidae)	Parasitoid insects
Pachyneuron aphidis (Bouché) (Hymenoptera: Pteromalidae)	Parasitoid insect
Paecilomyces (Hypocreales: Clavicipitaceae)	Nematopathogenic fungi
Paecilomyces fumosoroseus (Wize) (Hypocreales: Clavicipitaceae)	Nematopathogenic fungus
Paecilomyces lilacinus (Thom) Samson (Eurotiales: Trichocomaceae)	Nematopathogenic fungus
Paecilomyces sp. (Eurotiales: Trichocomaceae)	Nematopathogenic fungus
Paecilomyces tenuipes (Peck) Samson (Eurotiales: Trichocomaceae)	Nematopathogenic fungus
Paederus sp. (Coleoptera: Staphylinidae)	Predatory insect
Palpada mexicana (Macquart) (Diptera: Syrphidae)	Predatory insect
Palpada pusila (Macquart) (Diptera: Syrphidae)	Predatory insect
Palpada ruficeps (Macquart) (Diptera: Syrphidae)	Predatory insect
Palpada solennis (Walter) (Diptera: Syrphidae)	Predatory insect
Palpita persimilis Munroe (Lepidoptera: Crambidae)	Herbivorous insect
Palpozenillia diatraea Townsend (Diptera: Tachinidae)	Parasitoid insect
Palpozenillia palpalis Aldrich (Diptera: Tachinidae)	Parasitoid insect
Pammaecerus leptotrichopus (B.-B.) (Diptera:Tachinidae)	Parasitoid insect
Panonychus citri (McGregor) (Trombidiformes:Tetranychidae)	Herbivorous mite
Panonychus ulmi (Koch) (Trombidiformes: Tetranychidae)	Herbivorous mite
Pantoea agglomerans (Ewing and Fife) Gavini et al (Enterobacteriales: Enterobacteriacea)	Phytopathogenic bacterium
Pantomorus cervinus (Boh.) (Coleoptera: Curculionidae)	Herbivorous insect
Papilio demoleus L. (Lepidoptera: Papilionidae)	Herbivorous insect
Paracoccus marginatus Williams and Granara de Willink (Hemiptera: Pseudococcidae)	Herbivorous insect
Paracremonium (*Acremonium*) *pembeum* S.C. Lynch & Eskalen (Hypocreales: Hypocreaceae)	Phytopathogenic fungus
Parahormius pallidipes Ashm. (Hymenoptera: Braconidae)	Parasitoid insect
Parania tricolor (Szépligeti) (Hymenoptera: Ichneumonidae)	Parasitoid insect
Paranosema locustae Camming (Microsporidia)	Entomopathogenic fungus
Paratheresia claripalpis Wulp. (Diptera: Tachinidae)	Parasitoid insect
Pareuchaetes pseudoinsulata Rego Barros (Lepidoptera: Arctiidae)	Herbivorous insect
Parkinsonia aculeata L. (Fabales:Fabaceae)	Plant
Parlatoria oleae (Colvée) (Hemiptera: Diaspididae)	Herbivorous insect
Parthenium hysterophorus L. (Asterales:Asteraceae)	Plant
Parthenolecanium corni (Bouché) (Hemiptera: Coccidae)	Herbivorous insect

Parthenolecanium persicae (Fabricius) (Hemiptera: Coccidae)	Herbivorous insect
Passiflora edulis fo. flavicarpa O. Deg. (Malpighiales:Passifloraceae)	Plant
Passiflora edulis Sims. (Malpighiales:Passifloraceae)	Plant
Pasteuria nishizawae Sayre (Bacillales: Pasteuriaceae)	Nematopathogenic bacterium
Pasteuria penetrans (ex Thorne) Sayre & Starr (Bacillales: Pasteuriaceae)	Nematopathogenic bacterium
Patasson nitens (Girault) (Hymenoptera: Mymaridae)	Parasitoid insect
Pauridia peregrina (Timberlake) (Hymenoptera: Encyrtidae)	Parasitoid insect
Pectinophora gossipiella (Saunders) (Lepidoptera: Gelechiidae)	Herbivorous insect
Pectobacterium atrosepticum (Van Hall) (Enterobacteriales: Enterobacteriaceae)	Phytopathogenic bacterium
Pectobacterium carotovorum (Jones) Waldee subsp. Carotovorum (Enterobacteriales: Enterobacteriaceae)	Phytopathogenic bacterium
Pediobius cajanus Taveras & Hansson (Hymenoptera: Eulophidae)	Parasitoid insect
Pediobius furvus (Gah.) (Hymenoptera: Eulophidae)	Parasitoid insect
Peleopoda sp. (Lepidoptera: Oecophoridae)	Herbivorous insect
Peleopoda spp. (Lepidoptera: Oecophoridae)	Herbivorous insects
Pellioditis pellio (Schneider) (Nematoda: Rhabditidae)	Entomopathogenic nematode
Penicillium (Eurotiales: Trichocornaceae)	Antagonistic fungi
Penicillium notatum Thom (Eurotiales: Trichocornaceae)	Antogonistic fungus
Pentagona (Aulacaspis) pentagona Cockerell (Hemiptera: Diaspididae)	Herbivorous insect
Penthobruchus germaini (Pic.) (Coleoptera: Bruchidae)	Predatory insect
Pentilia castanea Muls (Coleoptera:Coccinellidae)	Predatory insect
Pentilia egena Muls (Coleoptera: Coccinellidae)	Predatory insect
Pentilia insidiosa Mulsant (Coleoptera: Coccinellidae)	Predatory insect
Pentilia spp. (Coleoptera: Coccinellidae)	Predatory insects
Perileucoptera coffeella Guer. (Lepidoptera: Lyonetidae)	Herbivorous insect
Perisierola nigrifemur Ashmead (Hymenoptera: Bethylidae)	Parasitoid insect
Peristenus relictus (= *stygicus*) Loan (Hymenoptera: Braconidae)	Parasitoid insect
Perkinsiella saccharicida Kirkaldy (Hemiptera: Delphacidae)	Herbivorous insect
Peronospora (Peronosporales: Peronosporaceae)	Phytopathogenic fungi
Persea americana Mill (Laurales:Lauraceae)	Plant
Pestalotia palmarum (Cooke) (Xylariales: Sporocadaceae)	Phytopathogenic fungus
Petrakina mirabilis Ciferri (Asterinaceae)	Phytopathogenic fungus
Petrusa epilepsis Kirkaldy (Hemiptera: Fulgoridae)	Herbivorus insect
Phalacrococcus howertoni Hodges & Hodgson (Hemiptera: Coccidae)	Herbivorous ninsect
Phalacrus obscurus Sharp (Coleoptera: Phalacridae)	Fungal spore feeding coleopteran
Phanerotoma bennetti Mues (Hymenoptera: Braconidae)	Parasitoid insect
Phanerotoma sp. (Hymenoptera: Braconidae)	Parasitoid insect
Phaseolus lunatus Linnaeus (Fabales:Fabaceae)	Plant
Phaseolus vulgaris L. (Fabales:Fabaceae)	Plant
Pheidole fallax Mayr (Hymenoptera: Formicidae)	Predatory insect
Pheidole megacephala (F.) (Hymenoptera: Formicidae)	Predatory insect
Pheidole radoszkowskii (Forel) (Hymenoptera: Formicidae)	Predatory insect
Phelister rufinotus Marseul (Coleoptera: Histeridae)	Predatory insect
Phenacoccus gossypii Townsend & Cockerell (Hemiptera: Pseudococcidae)	Herbivorous insect
Phenacoccus herreni Cox & Williams (Hemiptera: Pseudococcidae)	Herbivorous insect
Phenacoccus manihoti Matile-Ferrero (Hemiptera: Pseudococcidae)	Herbivorous insect
Pheropsophus aequinoctialis L. (Coleoptera: Carabidae)	Predatory insect

Philonthus quadraticeps Boheman (Coleoptera: Staphylinidae)	Predatory insect
Philornis downsi Dodge and Aitkem (Diptera: Muscidae)	Harmful insect
Phlugis teres DeGeer (Orthoptera: Tettigoniidae)	Predatory insect
Phlyctaenodes bifilalis Hamp. (Lepidoptera: Pyraustidae)	Herbivorous insect
Phoma sp. (Pleosporales: Didymellaceae)	Phytopathogenic fungi
Phoracantha Newman (Coleoptera: Cerambycidae)	Herbivorous insects
Phoracantha semipuntata (Fabricius) (Coleoptera: Cerambycidae)	Herbivorous insect
Phragmidium violaceum (Schultz) G. Winter (Uredinales: Phagmidiaceae)	Phytopathogenic fungus
Phthorimaea operculella (Zeller) (Lepidoptera: Gelechiidae)	Herbivorous insect
Phyllocnistis citrella Stainton (Lepidoptera: Gracillariidae)	Herbivorous insect
Phyllocoptruta oleivora (Ashmead) (Acari: Eriophyidae)	Herbivorous mite
Phyllophaga antiguae (Arr.) (Coleoptera: Scarabeidae)	Herbivorous insect
Phyllophaga smithi Flecha (Coleoptera: Scarabaeidae)	Herbivorous insect
Phyllophaga sp. (Coleoptera: Scarabaeidae)	Herbivorous insect
Phyllophaga spp. (Coleoptera: Scarabaeidae)	Herbivorous insects
Phyllosticta citricarpa (MacAlpine) (Botryosphaeriales: Botryosphaeriaceae)	Phytopathogenic fungus
Phymastichus coffea LaSalle (Hymenoptera: Eulophidae)	Parasitoid insect
Phymata fasciata (Gray) (Hemiptera: Reduviidae)	Predatory insect
Phymata sp. aff. *fortificata* (Herrich-Schäffer) (Hemiptera: Reduviidae)	Predatory insects
Phyrdenus sp. (Coleoptera: Curculionidae)	Herbivorous insects
Physalis peruviana L. (Solanales: Solanaceae)	Plant
Physalis sp. (Solanales: Solanaceae)	Plant
Phytomyptera sp. (Diptera: Tachinidae)	Parasitoid insect
Phytonemus pallidus (Banks) (Acari: Tarsonemidae)	Harmful mite
Phytophthora (Pernosporales: Phythiaceae)	Phytopathogenic fungi
Phytophthora cactorum (Lebert & Cohn) (Pythiales: Pythiaceae)	Phytopathogenic fungus
Phytophthora capsici Leonian (Peronosporales: Pythiaceae)	Phytopathogenic fungus
Phytophthora cinnamomi Rands (Peronosporales: Peronosporaceae)	Phytopathogenic fungus
Phytophthora infestans (Mont.) de Bary (Peronosporales: Peronosporaceae)	Phytopathogenic fungus
Phytophthora palmivora Butler (Peronosporales: Peronosporaceae)	Phytopathogenic fungus
Phytophthora sp. (Pythiales: Pythiaceae)	Phytopathogenic fungus
Phytophthora spp. (Pythiales: Pythiaceae)	Phytopathogenic fungi
Phytoseiulus longipes Evans (Acari: Phytoseiidae)	Predadory mite
Phytoseiulus macropilis Banks (Acari: Phytoseiidae)	Predatory mite
Phytoseiulus persimilis Athias-Henriot (Acari: Phytoseiidae)	Predatory mite
Phytoseiulus persimilis Athias-Henriot (Acari: Phytoseiidae)	Predatory mite
Phytoseiulus sp. (Acari: Phytoseiidae)	Predatory mite
Phytoseius dominicensis Ferragut & Moraes sp. nov. (Acari: Phytoseiidae)	Predatory mite
Piaractus mesopotamicus (Holmberg) (Characiformes: Characidae)	Fish
Pieris brassicae (L.) (Lepidoptera: Pieridae)	Herbivorous insect
Piezodorus guildinii (Westwood) (Hemiptera: Pentatomidae)	Herbivorous insect
Pimpla sp. (Hymenoptera: Ichneumonidae)	Parasitoid insects
Pineus boerneri Annand (Hemiptera: Adelgidae)	Herbivorous insect
Pinnaspis strachani Ferris and Rao (Hemiptera: Diaspididae)	Herbivorous insect
Pinus (Pinales: Pinaceae)	Plants
Pinus caribaea Morelet (Pinales: Pinaceae)	Plant
Pinus kesiya (Pinales:Pinaceae)	Plant

Pinus maximinoi (Pinales:Pinaceae)	Plant
Pinus oocarpa (Pinales:Pinaceae)	Plant
Pinus patula Schltdl. & Cham. (Pinales:Pinaceae)	Plant
Pinus radiata D. Don (Pinales: Pinaceae)	Plant
Pinus spp. (Pinales: Pinaceae)	Plants
Pinus tecunumanii (Pinales:Pinaceae)	Plant
Pissodes castaneus (De Geer) (Coleoptera: Curculionidae)	Herbivorous insect
Pistia stratiotes L. (Alismatales: Araceae)	Plant
Pitangus sulphuratus sulphuratus (L.) (Passeriformes: Tyrannidae)	Predatory bird
Plaesius javanus Erichson (Coleoptera: Histeridae)	Predatory insect
Plagioprospherysa trinitatis Thomps (Diptera: Tachinidae)	Parasitoid insect
Plagiotrypes sp. (Hymenoptera: Ichneumonidae)	Parasitoid insect
Planococcus citri Risso (Hemiptera: Pseudococcidae)	Herbivorous insect
Planococcus ficus (Signoret) (Hemiptera: Pseudococcidae)	Herbivorous insect
Planacoccus minor Maskell (Hemiptera: Pseudococcidae)	Herbivorous insect
Plasmodiophora brassicae Woronin (Plasmodiophorida: Plasmodiophoraceae)	Phytopathogenic fungus
Platystasius citri Nixon (Hymenoptera: Platygastridae)	Parasitoid insect
Plesiometa argyra (Walckenaer) (Araneae: Tetragnathidae)	Predatory spider
Plutella sp. (Lepidoptera: Plutellidae)	Herbivorous insect
Plutella xylostella (Linnaeus) (Lepidoptera: Plutellidae)	Herbivorous insect
Plutella xylostella polyhedrosis virus	Entomopathogenic virus
Pochonia (Hypocreales: Clavicipitaceae)	Nematophagous fungi
Pochonia chlamydosporia (Goddard) Zare & Gams (Hypocreales: Clavicipitaceae)	Nematophagous fungus
Pochonia chlamydosporia var. *catenulata* (Hypocreales: Clavicipitaceae)	Nematophagous fungus
Pochonia chlamydosporia var. *chlamydosporia* (Goddard) Gams and Zare (Hypocreales: Clavicipitaceae)	Nematophagous fungus
Podischnus agenor (Olivier) (Coleoptera: Scarabaeidae)	Herbivorous insect
Podisus connexivus Bergroth (Hemiptera: Pentatomidae)	Herbivorous insect
Podisus maculiventris (Say) (Hemiptera: Pentatomidae)	Herbivorous insect
Podisus nigrispinus (Dallas) (Hemiptera: Pentatomidae)	Herbivorous insect
Podisus sp. (Hemiptera: Pentatomidae)	Herbivorous insect
Podosphaera pannosa (Wallr.) de Bary (Erysiphaceae)	Phytophagous fungus
Poecilia reticulata Rosen & Bailey (Cyprinodontiformes: Poeciliidae)	Predatory fish
PoGV *Phthorimaea operculella*	Entomopathogenic virus
Polistes canadensis (Linné) (Hymenoptera: Vespidae)	Predatory insect
Polistes canadensis infuscatus Lep. (Hymenoptera: Vespidae)	Predatory insect
Polistes cavapyta Saussure (Hymenoptera: Vespidae)	Predatory insect
Polistes cinctus cinctus Lepeletier (Hymenoptera: Vespidae)	Predatory insect
Polistes cinctus barbadensis Richards (Hymenoptera: Vespidae)	Predatory insect
Polistes crinitus (Felton) (Hymenoptera: Vespidae)	Predatory insect
Polistes infuscatus ecuadorius Richards (Hymenoptera: Vespidae)	Predatory insect
Polistes panamensis Holmgren (Hymenoptera: Vespidae)	Predatory insect
Polistes sp. (Hymenoptera: Vespidae)	Predatory insect
Polistes spp. (Hymenoptera: Vespidae)	Predatory insects
Polistes versicolor (Olivier) (Hymenoptera: Vespidae)	Predatory insect
Polistes versicolor vulgaris Beq. (Hymenoptera: Vespidae)	Predatory insect
Polybia chrysothorax (Web.) (Hymenoptera: Vespidae)	Predatory insect

Polybia ignobilis (Haliday) (= *Polybia atra* Saussure) (Hymenoptera: Vespidae)	Predatory insect
Polybia liliacea (F.) (Hymenoptera: Vespidae)	Predatory insect
Polybia nigra Saussure (Hymenoptera: Vespidae)	Predatory insect
Polybia occidentalis (Olivier) (Hymenoptera: Vespidae)	Predatory insect
Polybia paulista Ihering (Hymenoptera: Vespidae)	Predatory insect
Polybia rejecta (F.) (Hymenoptera: Vespidae)	Predatory insect
Polybia scutellaris (White) (Hymenoptera: Vespidae)	Predatory insect
Polybia sericea (Oliv.) (Hymenoptera: Vespidae)	Predatory insect
Polybia sp. (Hymenoptera: Vespidae)	Predatory insect
Polybia striata (F.) (Hymenoptera: Vespidae)	Predatory insect
Polyhedrosis Virus (NPV)	Entomopathogenic virus
Polyphagotarsonemus latus (Banks) (Acari: Tarsonemidae)	Herbivorous mite
Pomacea dolioides (Reeve) (Ampullaridae)	Herbivorous snail
Portulaca oleracea L. (Caryophyllales: Portulacaceae)	Plant
Pouteria lucuma (Ruiz & Pav.) Kuntze (Ericales: Sapotaceae)	Plant
Praon gallicum Stary (Hymenoptera: Braconidae)	Parasitoid insect
Praon volucre (Haliday) (Hymenoptera: Braconidae)	Parasitoid insect
Praticolella griseola Pfeiffer (Gastropoda: Polygyridae)	Herbivorous snail
Pratylenchus brachyurus (Godfrey) Filipjev & S. Stekhoven (Tylenchida: Pratylenchidae)	Phytopathogenic nematode
Pratylenchus spp. (Tylenchida: Pratylenchidae)	Phytopathogenic nematodes
Pratylenchus zeae Graham (Tylenchida: Pratylenchidae)	Phytopathogenic nematode
Premnotrypes latithorax (Pierce) (Coleoptera: Curculionidae)	Herbivorous insect
Premnotrypes spp. (Coleoptera: Curculionidae)	Herbivorous insects
Premnotrypes vorax (Hustache) (Coleoptera: Curculionidae)	Herbivorous insect
Pristomerus (Hymenoptera: Ichneumonidae)	Parasitoid insects
Procalus mutans (Blanchard) (Coleoptera: Chrysomelidae)	Herbivorous insect
Procalus reduplicatus (Bechyné) (Coleoptera: Chrysomelidae)	Herbivorous insect
Prochiloneurus sp. (Hymenoptera: Encyrtidae)	Parasitoid insect
Prodilis (Neoporia) sp. (Coleoptera: Coccinellidae)	Predatory insect
Prodilis sp. (Coleoptera: Coccinellidae)	Predatory insect
Prodiplosis longifila Gagné (Diptera: Cecidomyiidae)	Herbivorous insect
Promicrogaster sp. (Hymenoptera: Braconidae)	Parasitoid insect
Proprioseiopsis sandersi (Chant) (Acari: Phytoseiidae)	Predatory mite
Proprioseiopsis sp. (Acari: Phytoseiidae)	Predatory mite
Prorops nasuta Waterston (Hymenoptera: Bethylidae)	Parasitoid insect
Prosapia simulans (Walker) (Hemiptera: Cercopidae)	Herbivorous insect
Prosapia spp. (Homoptera: Cercopidae)	Herbivorous insects
Prosopidopsilla flava (Homoptera: Psillidae)	Herbivorous insect
Prosopis ruscifoilia Griseb. (Fabales: Fabaceae)	Plant
Prosopis spp. (Fabales:Fabaceae)	Plants
Prospaltella berlessi How (Hymenoptera: Aphelinidae)	Parasitoid insect
Prospaltella opulenta Silvestri *(=Encarsia opulenta)* (Hymenoptera: Aphelinidae)	Parasitoid insect
Protolaccus bacchadis Burks (Hymenoptera: Pteromalidae)	Parasitoid insect
Protoparce sexta paphus (Cr.) (Lepdidoptera: Sphingidae)	Herbivorous insect
Protopulvinaria pyriformis (Cockerell) (Hemiptera: Coccidae)	Herbivorous insect
Pseudacteon bifidus Brown (Diptera: Phoridae)	Predatory insect
Pseudacteon curvatus Borgmeier (Diptera: Phoridae)	Predatory insect

Pseudacteon litoralis Borgmeier (Diptera: Phoridae)	Predatory insect
Pseudacteon sp. (Diptera: Phoridae)	Predatory insect
Pseudacteon tricuspis Borgemeier(Diptera: Phoridae)	Predatory insect
Pseudacysta perseae (Heidemann) (Hemiptera: Tingidae)	Herbivorus insect
Pseudaletia adultera (Schaus) (Lepidoptera: Noctuidae)	Herbivorous insect
Pseudaletia sequax (Franclemont) (Lepidoptera: Noctuidae)	Herbivorous insect
Pseudapanteles dignus Muesebeck (Hymenoptera: Braconidae)	Parasitoid insect
Pseudapanteles sp. (Hymenoptera: Braconidae)	Parasitoid insect
Pseudaphycus angelicus (Howard) (Hymenoptera: Encyrtidae)	Parasitoid insect
Pseudaphycus flavidulus (Bréthes) (Hymenoptera: Encyrtidae)	Parasitoid insect
Pseudaphycus utilis Timberlake (Hymenoptera: Encyrtidae)	Parasitoid insect
Pseudaulacaspis pentagona (Targioni) (Hemiptera: Diaspididae)	Herbivorous insect
Pseudleptomastrix mexicana Noyes e Schauff (Hymenoptera: Encyrtidae)	Parasitoid insect
Pseudoazya (Azya) trinitatis (Marshall) (Coleoptera: Coccinellidae)	Predatory insect
Pseudoazya trinitatis Gordon (Coleoptera: Coccinellidae)	Predatory insect
Pseudocercospora fijiensis (Morelet) (Capnodiales: Mycosphaerellaceae)	Phytopathogenic fungus
Pseudococcus calceolariae (Maskell) (Hemiptera: Pseudococcidae)	Herbivorous insect
Pseudococcus elisae Borchsenius (Hemiptera: Pseudococcidae)	Herbivorous insect
Pseudococcus longispinus (Targioni & Tozzetti) (Hemiptera: Pseudococcidae)	Herbivorous insect
Pseudococcus spp. (Hemiptera: Pseducoccidae)	Herbivorous insects
Pseudococcus viburni (Signoret) (Hemiptera: Pseudococcidae)	Herbivorous insect
Pseudococcus Westwood (Hemiptera: Pseudococcidae)	Herbivorous insects
Pseudodorus clavatus (F.*)* (Diptera: Syrphidae)	Predatory insect
Pseudogonatopus saccharivorae Richards (Hymenoptera: Dryinidae)	Parasitoid insect
Pseudomonas (Pseudomonadales: Pseudomonadaceae)	Phytopathogenic and antagonistic bacteria
Pseudomonas aeruginosa (Schroter) Migula (Pseudomonadales: Pseudomonadaceae)	Antagonistic bacterium
Pseudomonas fluorescens Flügge (Pseudomonadales: Pseudomonadaceae)	Antagonistic bacterium
Pseudomonas putida Trevisan (Pseudomonadales: Pseudomonadaceae)	Antagonistic bacterium
*Pseudomonas solanacearum (*Smith) *(= Ralstonia solanacearum)* (Pseudomonadales: Pseudomonadaceae)	Phytopathogenic bacterium
Pseudomonas spp. (Pseudomonales: Pseudomonadaceae)	Phytopathogenic and antagonistic bacteria
Pseudoparasitus sp. (Acari: Laelapidae)	Predatory mite
Pseudophycus perdignus (Compere) (Hymenoptera: Encyrtidae)	Parasitoid insect
Pseudoplusia (= *Chrysodeixis*) *includens* (Walker) (Lepidoptera: Noctuidae)	Herbivorous insect
Psidium guajava L. (Myrtales: Myrtaceae)	Plant
Psyllaeaphagus blitens Riek (Hymenoptera: Encyrtidae)	Parasitoid insect
Psyllaephagus pilosus Noyes (Hymenoptera: Encryrtidae)	Parasitoid insect
Psyllaephagus yaseeni Noyes (Hymenoptera: Encyrtidae)	Parasitoid insect
Psyllobora divisa (F.) (Coleoptera: Coccinellidae)	Predatory insect
Psyttalia concolor (Szepligati) (Hymenopteraa: Braconidae)	Parasitoid insect
Psyttalia incise (Silvestri) (Hymenoptera: Braconidae)	Parasitoid insect
Pteromalus puparum (Linné) (Hymenoptera: Pteromalidae)	Parasitoid insect
Pteroptrix smithi (Compere) Hymenoptera: Aphelinidae)	Parasitoid insect
Pterostichus sp. (Coleoptera: Carabidae)	Predatory insect
Puccinia chondrillina (Bubak & Syd.) (Puccinales:Pucciniaceae)	Phytopathogenic fungus
Puccinia gramimis f.sp. *raza* Ug99 (Pucciniales: Puccinaceae)	Phytopathogenic fungus

Pullus gilae (Casey) (Coleoptera: Coccinellidae)	Predatory insect
Pullus loewii Mulsant (Coleoptera: Coccinellidae)	Predatory insect
Pullus spp. (Coleoptera: Coccinellidae)	Predatory insects
Pulvinaria mesembryanthemi Vallot (Hemiptera: Coccidae)	Herbivorous insect
Pulvinaria psidii Maskell (Hemiptera: Coccidae)	Herbivorous insect
Pulvinaria pyriformis (Ckll.) (Hemiptera: Coccidae)	Herbivorous insect
Purpureocillium (Hypocreales: Ophiocordycipitaceae)	Nematopathogenic fungi
Purpureocillium lilacinum (Thom) Luangsaard, Hou-braken, Hywl-Jones and Samson (Hypocreales: Ophiocordycipitaceae)	Nematopathogenic fungus
Puto barberi (Ckll.) (Hemiptera: Pseudococcidae)	Herbivorous insect
Pyemotes ventricosus (Newport) (Acarina: Pyemotidae)	Predatory mite
Pyrenochaeta lycopersici (Schneider & Gerlach) (Pleosporales)	Phytopathogenic fungus
Pyricularia oryzae Cavara (Magnaporthales: Magnaporthaceae)	Phytopathogenic fungus
Pyricularia sp. (Magnaporthaceae)	Phytopathogenic fungi
Pyrophorus luminosus Illiger (Coleoptera: Elateridae)	Predatory insect
Pythium (Peronosporales: Pythiaceae)	Phytopathogenic fungi
Pythium sp. (Peronosporales: Pythiaceae)	Phytopathogenic fungus
Pythium ultimum Trow (Peronosporales: Pythiaceae)	Phytopathogenic fungus
Q	
Quadraspidiotus perniciosus Comstock (Hemiptera: Diaspididae)	Herbivorous insect
Quadrastichus erythrinae Kim (Hymenoptera: Eulophidae)	Parasitoid insect
R	
Rachiplusia nu (Guenée) (Lepidoptera: Noctuidae)	Herbivorous insect
Radopholus similis (Cobb) Thorne (Tylenchida: Pratylenchidae)	Phytopathogenic nematode
Raffaelea lauricola T.C. Harr., Fraedrich & Aghayeva (Ophiostomatales: Ophiostomataceae)	Phytopathogenic fungus
Ralstonia solanacearum (Smith) (Burkholderiales: Burkoholderiaceae)	Phytopathogenic bacteria
Ralstonia solanacearum raza 2 (Smith)(Burkholderiales: Burkoholderiaceae)	Phytopathogenic bacteria
Raoiella indica Hirst (Acari: Tenuipalpidae)	Herbivorous mite
Rastrococcus invadens Williams (Hemiptera: Pseudococcidae)	Herbivorous insect
Rattus argentiventer (Robinson & Kloss) (Rodentia: Muridae)	Harmful mammal
Rattus rattus L. (Rodentia: Muridae)	Harmful mammal
Rattus sp. (Rodentia: Muridae)	Harmful mammal
Repipta sp. (Hemiptera: Reduviidae)	Predatory insect
Rhaconotus rosiliensis Lal. (Hymenoptera: Braconidae)	Parasitoid insect
Rhaconotus sp. (Hymenoptera: Braconidae)	Parasitoid insect
*Rhaphitelus maculatu*s Walker (Hymenoptera: Pteromalidae)	Parasitoid insect
Rhigopsidius tucumanus Heller (Coleoptera: Curculionidae)	Herbivorous insect
Rhinella marina L. (Anura: Bufonidae)	Predatory amphibian
Rhingia nigra Mcquart (Diptera: Syrphidae)	Predatory insect
Rhinocyllus conicus Frölich (Coleoptera: Curculionidae)	Herbivorous insect
Rhinoleucophenga sp. (Diptera: Drosophilidae)	Herbivorous insect
Rhipicephalus spp. (Ixodida: Ixodidae)	Harmful ticks
Rhizaspidiotus donacis (Leonardi) (Hemiptera: Diaspididae)	Herbivorous insect
Rhizobius lophantae (Blaisd) (Coleoptera: Coccinellidae)	Predatory insect
Rhizoctonia solani Kuhn (Cantharellales: Ceratobasidiaceae)	Phytopathogenic fungus

Rhizoctonia sp. (Cantharellales:Ceratobasidiaceae)	Phytopathogenic fungus
Rhizoctonia spp. (Cantharellales: Ceratobasidiaceae)	Phytopathogenic fungi
Rhodococcus chubuensis Tsukamura (Actinomycetales: Nocardiaceae)	Phytopathogenic bacterium
Rhodotorula glutinis (Fresenius) Harrison (Sporidiobolales: Sporidiobolaceae)	Antagonistic
Rhopalosiphum (Hemiptera: Aphididae)	Herbivorous insect
Rhopalosiphum maidis (Fitch) (Hemiptera: Aphididae)	Herbivorous insect
Rhyacionia buoliana (Denis & Schiffermüller) (Lepidoptera: Tortricidae)	Herbivorous insect
Rhyacionia frustrana (Comstock) (Lepidoptera: Tortricidae)	Herbivorous insect
Rhynchophorus palmarum L. (Coleoptera: Curculionidae)	Herbivorous insect
Rhynchosia minima (L.) DC. (Fabales: Fabaceae)	Plant
Rhynchosia reticulata (Sw.) DC. (Fabaceae)	Plant
Rhyssa persuasoria (L.) (Hymenoptera: Icchneumonidae)	Parasitoid insect
Rhyssomatus marginatus Fahraeus (Coleoptera: Curculionidae)	Herbivorous insect
Rhyzobius purchellus Montrozier (Coleoptera: Coccinellidae)	Predatory insect
Rhyzobius ventralis (Erichson) (Coleoptera: Coccinellidae)	Predatory insect
Ricinus communis L. (Malpighiales: Euphorbiaceae)	Plant
Rizoctonia (Cantharellales: Ceratobasidiaceae)	Antagonistic and Entomopathogenic fungi
Rodolia (Novius) cardinalis Mulsant (Coleoptera: Coccinellidae)	Predatory insect
Rogas aligarhensis (Quadri) (Hymenoptera: Braconidae)	Parasitoid insect
Rogas gossypii Muesebeck (Hymenoptera: Braconidae)	Parasitoid insect
Rogas spp. (Hymenoptera: Braconidae)	Parasitoid insects
Rogas vaughani Muesebeck (Hymenoptera: Braconidae)	Parasitoid insect
Romanomermis culicivorax Ross & Smith (Rhabditida: Mermithidae)	Entomopathogenic nematode
Romanomermis iyengari (Welch) (Rhabditida: Mermithidae)	Entomopathogenic nematode
Rosellinia sp. (Xylariales: Xylariaceae)	Phytopathogenic fungus
Rostrhamus sociabilis (Vieillot) (Accipitriformes: Accipitridae)	Predatory bird
Rothschildia aroma Schaus (Lepidoptera: Saturniidae)	Herbivorous insect
Rubus fruticosus L. (Rosales: Rosaceae)	Plant
Rubus glaucus Benth. (Rosales: Rosaceae)	Plant
Rubus niveus Thunb. (Rosales: Rosaceae)	Plant
Rubus spp. (Rosales: Rosaceae)	Plants
Rubus ulmifolius (Schott.) (Rosales: Rosaceae)	Plant
Rupela albinella (Cr.) (Lepidoptera: Pyralidae)	Herbivorous insect
Rutela lineola (L.) (Coleoptera: Scarabaeidae)	Herbivorous insect
Rynchophorus ferrugineus Olivier (Coleoptera: Curculionidae)	Herbivorous insect
S	
Sacadodes pyralis (Dyar) (Lepidoptera: Noctuidae)	Herbivorous insect
Saccharicoccus sacchari Cockerell (Hemiptera: Pseudococcidae)	Herbivorous insect
Saccharosydne saccharivora (Westwood) (Hemiptera: Delphacidae)	Herbivorous insect
Sagalassa valida Walker (Lepidoptera: Glyphipterigidae)	Herbivorous insect
Saimiri sciureus (L.) (Primatas: Cebidae)	Predatory mammal
Saissetia coffeae (Walker) (Hemiptera: Coccidae)	Herbivorous insect
Saissetia nigra (Nietner) (Hemiptera: Coccidae)	Herbivorous insect
Saissetia oleae Olivier (Hemiptera: Coccidae)	Herbivorous insect
Saissetia spp. (Hemiptera: Coccidae)	Herbivorous insects

Salpichroa origanifolia (Lam.) Baill (Solanales: Solanaceae)	Plant
Salpingogaster nigra Shiner (Diptera: Syrphidae)	Predatory insect
Salvinia molesta D. Mitch. (Polypodiopsida: Salvinaceae)	Plant
Sarcodexia sternodontis Townsend (Diptera: Sarcophagidae)	Parasitoid insect
Sarcophaga acridiorum Weyenberg (Diptera: Sarcophagidae)	Parasitoid insect
Sarcophaga caridei Brèthes (Diptera: Sarcophagidae)	Parasitoid insect
Sarcophaga sp. (Diptera: Sarcophagidae)	Parasitoid insect
Sarocladium oryzae (Sawada) W. Gams & D. Hawksw. (Ascomycetes)	Phytopathogenic fungus
Sarucallis kahawaluokalani (Kirkaldy) (Hemiptera: Aphididae)	Herbivorous insect
Scapteriscus abbreviatus Scudder (Orthoptera: Gryllotalpidae)	Herbivorous insect
Scapteriscus didactylus (Latreille) (Orthoptera: Gryllotalpidae)	Herbivorous insect
Scapteriscus spp. (Orthoptera: Gryllotalpidae)	Herbivorous insects
Scapteriscus vicinus Latr (Orthoptera: Gryllotalpidae)	Herbivorous insect
Scaptia lata (Guérin-Méneville) (Diptera: Tabanidae)	Harmful insect
Scelio aegyptiacus Priesner (Hymenoptera: Scelionidae)	Parasitoid insect
Scelio famelicus (Say) (Hymenoptera: Scelionidae)	Parasitoid insect
Scelio sp. nr. *serdangensis* (Timb.) (Hymenoptera: Scelionidae)	Parasitoid insect
Schistocerca americana (Drury) (Orthoptera: Acrididae)	Herbivorous insect
Schistocerca paranensis (Burmeister & H.) (Orthoptera: Acrididae)	Herbivorous insect
Schistocerca cancellata (Serville) (Orthoptera: Acrididade)	Herbivorous insect
Schistocerca interrita Scudder (Orthoptera: Acrididae)	Herbivorous insect
Schistocerca pallens (Thnb.) (Orthoptera: Acrididae)	Herbivorous insect
Schistocerca piceifrons piceifrons Walker (Orthoptera: Acrididae)	Herbivorous insect
Schistocerca spp. (Orthoptera: Acrididae)	Herbivorous insects
Schistosoma mansoni Sambon (Diplostomida: Schistosomatidae)	Harmful invertebrate
Schizaphis graminum (Rondani) (Hemiptera: Aphididae)	Herbivorous insect
Scirtothrips dorsalis Hood (Thysanoptera: Thripidae)	Herbivorous insect
Scleria melaleuca Rchb. F.ex. Schltdl. Cham. (Poales: Cyperaceae)	Plant
Sclerotinia (Helotiales: Sclerotiniaceae)	Phytopathogenic fungi
Sclerotinia minor (Jagger) (Helotiales: Sclerotiniaceae)	Phytopathogenic fungus
Sclerotinia sclerotiorum (Lib.) de Bary (Helotiales: Sclerotiniaceae)	Phytopathogenic fungus
Sclerotium (Atheliales: Atheliaceae)	Phytopathogenic fungi
Sclerotium cepivorum Berk. (Atheliales: Atheliales)	Phytopathogenic fungus
Sclerotium rolfsi Sacc (Atheliales: Atheliaceae)	Phytopathogenic fungus
Scolytus rugulosus (Muller) (Coleoptera: Curculionidae)	Herbivorous insect
Scolothrips sp. (Thysanoptera: Thripidae)	Herbivorous insect
Scotussa lemniscata (Stal (Orthoptera: Acrididae)	Herbivorous insect
Scrobipalpula (Lepidoptera: Gelechiidae)	Herbivorous insect
Scutellista (Hymenoptera: Pteromalidae)	Parasitoid insects
Scutellista cyanea Motschulsky (Hymenoptera: Pteromalidae)	Parasitoid insect
Scymnus coccivora Aiyyar (Coleoptera:Coccinellidae)	Predatory insect
Scymnus rubicundus Erichson (Coleoptera: Coccinellidae)	Predatory insect
Scymnus smithianus Silvestri (Coleoptera: Coccinellidae)	Predatory insect
Scymnus sp. (Coleoptera: Coccinellidae)	Predatory insect
Scymnus spp. (Coleoptera: Coccinellidae)	Predatory insects
Selenaspidus articulatus Morgan (Hemiptera: Diaspididae)	Herbivorous insect
Selenothrips rubrocinctus (Giard) (Thysanoptera: Thripidae)	Herbivorous insect

Selitrichodes neseri Kelly & La Salle (Hymenoptera: Eulophidae)	Parasitoid insect
SeNPV *Spodoptera exigua*	Entomopathogenic virus
Sesbania punicea (Cav.) (Fabales: Fabaceae)	Plant
SfMNPV (*S. frugiperda* nucleopolyhedrovirus)	Entomopathogenic virus
Sibine fusca (Stoll) (Lepidoptera: Limacodidae)	Herbivorous insect
Sibine sp. (Lepidoptera: Limacodidae)	Herbivorous insect
Sida spp. (Malvales: Malvaceae)	Plants
Signiphora aleyrodis Ashmead (Hymenoptera: Signiphoridae)	Parasitoid insect
Signiphora sp. (Hymenoptera: Signiphoridae)	Parasitoid insect
Silybum spp. (Asterales: Asteraceae)	Plants
Simplicillium (Hypocreales: Cordycipitaceae)	Entomopathogenic fungi
Simplicillium spp. (Hypocreales: Cordycipitaceae)	Entomopathogenic fungi
Sinea sp. (Hemiptera: Reduviidae)	Predatory insect
Singhiella simplex (Singh) (Hemiptera: Aleyrodidae)	Herbivorous insect
Sipha flava (Forbes) (Hemiptera: Aphididae)	Herbivorous insect
Sirentha carinata (Fabricius) (Hemiptera: Reduviidae)	Predatory insect
Sirex (Hymenoptera: Siricidae)	Herbivorous insects
Sirex noctilio Fabricius (Hymenoptera: Siricidae)	Herbivorous insect
Sitobion avenae (Fabricius) (Hemiptera: Aphididae)	Herbivorous insect
Sitophylus zeamais (Motschulsky) (Coleoptera: Curculionidae)	Herbivorous insect
Sitotroga cerealella Oliver (Lepidoptera: Gelechiidae)	Herbivorous insect
Sitotroga sp. (Lepidoptera: Gelechiidae)	Herbivorous insect
Smicra (= Conura) punctata (Fabricius) (Hymenoptera: Chalcididae)	Parasitoid insect
Smicra emarginata (Fabricius) (Hymenoptera: Chalcididae)	Parasitoid insect
Smicra flavopicta Cresson (Hymenoptera: Chalcididae)	Parasitoid insect
Smicra ignea (Cresson) (Hymenoptera: Chalcididae)	Parasitoid insect
Smicronyx roridus Marshall (Coleoptera: Curculionidae)	Herbivorous insect
Smicronyx rufovittatus Anderson (Coleoptera: Curculionidae)	Herbivorous insect
Sogatodes oryzicola (Muir) (Homoptera: Delphacidae)	Herbivorous insect
Sogatodes oryzicola (Muir) (Homoptera: Delphacidae)	Herbivorous insect
Solanum (Solanales: Solanaceae)	Plants
Solanum americanum Mill. (Solanales: Solanaceae)	Plant
Solanum betacea Cav. (Solanales: Solanaceae)	Plant
Solanum jamaicense Mill (Solanales: Solanaceae)	Plant
Solanum lycopersicum L. (Solanales: Solanaceae)	Plant
Solanum melongena L. (Solanales: Solanaceae)	Plant
Solanum quitoense var. Septentrional Lam. (Solanales: Solanaceae)	Plant
Solanum syssimbrifolium Lam (Solanales: Solanaceae)	Plant
Solanum torvum Sw. (Solanales: Solanaceae)	Plant
Solanum tuberosum L. (Solanales: Solanaceae)	Plant
Solanum tuberosum L. spp. *andigena* (Solanales: Solanaceae)	Plant
Solanum tuberosum L. spp. *tubersoum* (Solanales: Solanaceae)	Plant
Solanum viarum Dunal (Solanales:Solanaceae)	Plant
Solenopsis geminata F. (Hymenoptera: Formicidae)	Predatory / harmful insect
Solenopsis invicta Buren (Hymenoptera: Formicidae)	Predatory / harmful insect
Solenopsis richteri Forel (Hymenoptera: Formicidae)	Predatory / harmful insect
Solenopsis sp. (Hymenoptera: Formicidae)	Predatory / harmful insect

Solenopsis spp. (Hymenoptera: Formicidae)	Predatory / harmful insects
Sonchus oleraceous L. (Asterales: Asteraceae)	Plant
Sorghum bicolor (L.) Moensch (Poales: Poaceae)	Plant
Spalangia cameroni (Perkins) (Hymenoptera: Pteromalidae)	Parasitoid insect
Spalangia endius (Walker) (Hymenoptera: Pteromalidae)	Parasitoid insect
Spalangia gemina Boucek (Hymenoptera: Pteromalidae)	Parasitoid insect
Spalangia nigra Latreille (Hymenoptera: Pteromalidae)	Parasitoid insect
Spalangia nigroaenea Curtis (Hymenoptera: Pteromalidae)	Parasitoid insect
Spalangia simplex Perkins (Hymenoptera: Pteromalidae)	Parasitoid insect
Spalangia sp. (Hymenoptera: Pteromalidae)	Parasitoid insect
Spalangia spp. (Hymenoptera: Peromalidae)	Parasitoid insects
SpeVG *Spodoptera eridania*	Entomopathogenic virus
SpeVPN *Spodoptera eridania*	Entomopathogenic virus
SpfVG *Spodoptera frugiperda*	Entomopathogenic virus
SpfVPN *Spodoptera frugiperda*	Entomopathogenic virus
Sphaerotheca fuliginea (Schltdl.) Pollacci (Erysiphales: Erysiphaceae)	Phytopathogenic fungus
Sphaerotheca macularis (Wallr.) (Erysiphales: Erysiphaceae)	Phytopathogenic fungus
Sphegigaster sp. (Hymenoptera: Pteromalidae)	Parasitoid insect
Sphenarium purpurascens Charpentier (Orthoptera: Pyrgomorphidae)	Herbivorous insect
Sphenophorus levis Vaurie (Coleoptera:Curculionidae)	Herbivorous insect
Sphictyrtus sp. (Hemiptera: Coreidae)	Herbivorous insect
Sphiximorpha barbipes (Loew) (Diptera: Syrphidae)	Predatory insect
Spicaria javanica Bally (Hypocreales: Cordycipitaceae)	Entomopathogenic fungus
Spilocalcis sp. (Hymenoptera: Chalcididae)	Parasitoid insect
Spilochalcis dux (Walker) (Hymenoptera: Chalcididae)	Parasitoid insect
Spilochalcis fulvomaculata (Cameron) (Hymenoptera: Chalcididae)	Parasitoid insect
Spilochalcis hirtifemora (Ashmead.) (Hymenoptera: Chalcididae)	Parasitoid insect
Spilochalcis torvina (Cress.) (Hymenoptera: Chalcididae)	Parasitoid insect
Spiracantha cornifolia Kunth (Asterales: Asteraceae)	Plant
SpocVPN *Spodoptera ochrea*	Entomopathogenic virus
Spodoptera (= Prodenia) eridania (Cram.) (Lepidoptera: Noctuidae)	Herbivorous insect
Spodoptera (Lepidoptera: Noctuidae)	Herbivorous insects
Spodoptera dolichos (F.) (Lepidoptera: Noctuidae)	Herbivorous insect
Spodoptera eridania (Cram.) (Lepidoptera: Noctuidae)	Herbivorous insect
Spodoptera exigua (Hübner) (Lepidoptera: Noctuidae)	Herbivorous insect
Spodoptera frugiperda (J.E. Smith*)* (Lepidoptera: Noctuidae)	Herbivorous insect
Spodoptera latifascia (Wlk.) (Lepidoptera: Noctuidae)	Herbivorous insect
Spodoptera omithogalli (Gn.) (Lepidoptera: Noctuidae)	Herbivorous insect
Spodoptera sp. (Lepidoptera: Noctuidae)	Herbivorous insect
Spodoptera spp. (Lepidoptera: Noctuidae)	Herbivorous insects
Spodoptera sunia (Guenee) (Lepidoptera: Noctuidae)	Herbivorous insect
Spondias mombin L. (Sapindales: Anacardiaceae)	Plant
Spondias sp. (Sapindales: Anacardiaceae)	Plant
Sporothrix (Ophiostomatales: Ophiostomataceae)	Fungal pathogenic fungus
Stantonia sp. (Hymenoptera: Braconidae)	Parasitoid insect
Stegasta basquella (Chamb.) (Lepidoptera:Gelechiidae)	Herbivorous insect
Steinernema (Rhabditida: Steinernematidae)	Entomopathogenic nematode

Steinernema australe Edgington, Buddie, Tymo, Hunt, Nguyen, France, Merino & Moore (Rhabditida: Steinernematidae)	Entomopathogenic nematode
Steinernema bibionis Bobien (Rhabditida: Steinernematidae)	Entomopathogenic nematode
Steinernema brazilense Nguyen, Ginarte, Leite, Santo & Harakava (Rhabditida: Steinernematidae)	Entomopathogenic nematode
Steinernema carpocapsae (Weiser) Wouts, Mracek, Gerdin & Bedding (Rhabditida: Steinernematidae)	Entomopathogenic nematode
Steinernema feltiae (Fiiipjev) Wouts, Mracek, Gerdin & Bedding (Rhabditida: Steinernematidae)	Entomopathogenic nematode
Steinernema glaseri (Steiner) Wouts, Mracek, Gerdin & Bedding (Rhabditidae: Steinernematidae)	Entomopathogenic nematode
Steinernema puertoricense Roman & Figueroa(Rhabditidae: Steinernematidae)	Entomopathogenic nematode
Steinernema sp. (Nematoda: Steinernematidae)	Entomopathogenic nematode
Steinernema spp. (Nematoda: Steinernematidae)	Entomopathogenic nematodes
Steinernema unicornum Egington, Buddie Tymo, France, Merino & Hunt (Rhabditidae: Steinernematidae)	Entomopathogenic nematode
Steneotarsonemus konoi Smiley & Enmanouel (Acari: Tarsonemidae)	Herbivorous mite
Steneotarsonemus spinki Smiley (Acari: Tarsonemidae)	Herbivorous mite
Stenocranophilus quadratus Pierce (Strepsiptera: Halictophagidae)	Parasitoid insect
Stenoma cecropia (Meyrick) (Lepidoptera: Elachistidae)	Herbivorous insect
Stenoma impressella (Busck) (Lepidoptera: Elachistidae)	Herbivorous insect
Stenoma sp. (Lepidoptera: Elachistidae)	Herbivorous insect
Stenopelmus rufinasus Gyllenhal (Coleoptera: Erirhinidae)	Herbivorous insect
Sternechus subsignatus Boheman (Coleoptera: Curculionidae)	Herbivorous insect
Stethorus caribus Gordon & Chapin (Coleoptera: Coccinellidae)	Predatory insect
Stethorus punctillum (Weise) (Coleoptera: Coccinellidae)	Predatory insect
Stethorus salutaris Kapur (Coleoptera: Coccinellidae)	Predatory insect
Stethorus sp. (Coleoptera: Coccinelidae)	Predatory insect
Stomoxys calcitrans (L.) (Diptera: Muscidae)	Harmful insect
Stomoxys spp. (Diptera: Muscidae)	Harmful insects
Strabotes rupelae nov. spec Zwart (Hymenoptera: Ichneumonidae)	Parasitoid insect
Stratiolaelaps scimitus Womersley (Acari: Laelapidae)	Predatory mite
Streptomyces (Actinomycetales: Streptomycetaceae)	Plant pathogenic bacteria
Streptomyces racemochromogenes Sugai (Actinomycetales: Streptomycetaceae)	Plant pathogenic bacterium
Strymon basalides H. (Lepidoptera: Lycaenidae)	Herbivorous insect
Stylopage hadra Drechsler (Zoopagales: Zoopagaceae)	Nematophagous fungus
Sulcophanaeus menelas (Laporte) (Coleoptera: Scarabaeidae)	Herbivorous insect
Swietenia macrophylla G. King (Sapindales: Meliaceae)	Plant
Swietenia mahagoni Jacq. (Sapindales: Meliaceae)	Plant
Swietenia spp. (Meliaceae)	Plants
Swinglea glutinosa (Blanco) Merr. (Sapindales: Rutaceae)	Plant
Syllepte helcitalis (Wlk.) (Lepidoptera: Crambidae)	Herbivorous insect
Symmetrischema tangolias (Gyer.) (Lepidoptera: Gelechiidae)	Herbivorous insect
Sympherobius barberi (Banks) (Neuroptera: Hemerobiidae)	Predatory insect
Sympherobius sp. (Neuroptera: Hemerobiidae)	Predatory insect
Syneura cocciphila (Coquillett) (Diptera: Phoridae)	Predatory insect
Synoeca cyanea (Fabricius) (Hymenoptera: Vespidae)	Predatory insect
Synopeas sp. (Hymenoptera: Platygasteridae)	Parasitoid insect
Syntomosphyrum indicum Silv. (Hymenoptera: Eulophidae)	Parasitoid insect
Syrphophagus aphidivorus (Mayr) (Hymenoptera: Encyrtidae)	Parasitoid insect

Syrphophagus nigricornis (De Santis) (Hymenoptera: Encyrtidae)	Parasitoid insect
Syrphophagus sp. (Hymenoptera: Encyrtidae)	Parasitoid insect
Syrphus (Diptera: Syrphidae)	Predatory insects
Systoechus vulgaris Loew (Diptera: Bombyliidae)	Predatory insect
Syzygium samarangense (Blume) Merr. & Perry (Myrtales: Myrtaceae)	Plant
T	
Tachinaephagus zealandicus Ashmead (Hymenoptera: Encyrtidae)	Parasitoid insect
Tamarixia leucaenae Boucek (Hymenoptera: Eulophidae)	Parasitoid insect
Tamarixia radiata (Waterston) (Hymenoptera: Eulophidae)	Parasitoid insect
Tamarixia triozae (Burks) (Hymenoptera: Eulophidae)	Parasitoid insect
Tapinoma melanocephalum (Fab.) (Hymenoptera: Formicidae)	Predatory insect
Tarophagous esculenta (Homoptera: Delphacidae)	Herbivorous insect
Tecia solanivora (Povolný) (Lepidoptera: Gelechiidae)	Herbivorous insect
Tectona grandis L. (Lamiales: Lamiaceae)	Plant
Telchin licus (Drury) (Lepidoptera: Castniidae)	Herbivorous insect
Telenomus (Hymenoptera: Platigastridae)	Parasitoid insects
Telenomus alecto Crawford (Hymenoptera: Platigastridae)	Parasitoid insect
Telenomus alsophilae Viereck (Hymenoptera: Platigastridae)	Parasitoid insect
Telenomus basalis (Wollaston) (Hymenoptera: Platigastridae)	Parasitoid insect
Telenomus connectans Ashm (Hymenoptera: Platigastridae)	Parasitoid insect
Telenomus dilophonotae Cam. (Hymenoptera: Platigastridae)	Parasitoid insect
Telenomus fariai Costa Lima (Hymenoptera: Platigastridae)	Parasitoid insect
Telenomus nawaii Ash. (Hymenoptera: Platigastridae)	Parasitoid insect
Telenomus nigrocoxalis Ashm (Hymenoptera: Platigastridae)	Parasitoid insect
Telenomus podisi Ashmead (Hymenoptera: Platigastridae)	Parasitoid insect
Telenomus remus Nixon (Hymenoptera: Platigastridae)	Parasitoid insect
Telenomus rowani (Gahan) (Hymenoptera: Platigastridae)	Parasitoid insect
Telenomus sp. (Hymenoptera: Platigastridae)	Parasitoid insect
Telenomus sp. near *alecto* (Hymenoptera: Platigastridae)	Parasitoid insect
Telenomus sp. prob. *dilophonotae* Cam. (Hymenoptera: Platigastridae)	Parasitoid insect
Telsimia nitida Chapin (Coleoptera: Coccinellidae)	Predatory insect
Telsimia sp. (Coleoptera: Coccinellidae)	Predatory insect
Temelucha sp. (Hymenoptera: Ichneumonidae)	Parasitoid insect
Thelohania solenopsae (J.D. Knell, G.E. Allen & E.I. Hazard) (Microsporida: Thelohaniidae)	Parasitic protozoan
Termatophylidea opaca Carvalho (Hemiptera: Miridae)	Predatory insect
Tetracnemus pretiosus (Timberlake) (Hymenoptera: Encyrtidae)	Parasitoid insect
Tetramesa romana (Walker) (Hymenoptera: Eurytomidae)	Herbivorous insect
Tetranychus cinnabarinus (Boisduval) (Acari: Tetranychidae)	Herbivorous mite
Tetranychus evansi Banks & Pritchard (Acari: Tetranychidae)	Herbivorous mite
Tetranychus gloveri Banks (Acari: Tetranychidae)	Herbivorous mite
Tetranychus lintearius (Dufour) (Acari: Tetranychidae)	Herbivorous mite
Tetranychus spp. (Acari: Tetranychidae)	Herbivorous mites
Tetranychus tumidus (Banks) (Acari: Tetranychidae)	Herbivorous mite
Tetranychus urticae Koch (Acari: Tetranychidae)	Herbivorous mite
Tetrastichus (= Oomyzus) sokolowski Kurd. (Hymenoptera:Eulophidae)	Parasitoid insect

Tetrastichus gala Walker (= *Tetrastichus marylandensis)* (Hymenoptera:Eulophidae)	Parasitoid insect
Tetrastichus gala Walker (Hymenoptera:Eulophidae)	Parasitoid insect
Tetrastichus gallerucae (Fonscolmber) (Hymenoptera: Eulophidae)	Parasitoid insect
Tetrastichus giffardianus Silvestri (Hymenoptera: Eulophidae)	Parasitoid insect
Tetrastichus haitiensis Gah. (Hymenoptera: Eulophidae)	Parasitoid insect
Tetrastichus howardi (Olliff) (Hymenoptera: Eulophidae)	Parasitoid insect
Tetrastichus sp. (Hymenoptera: Eulophidae)	Parasitoid insect
Tetrastichus sp. nr. *vaquitarum* Wolc. (Hymenoptera: Eulophidae)	Parasitoid insect
Tetrastichus spirabilis Waterston (Hymenoptera: Eulophidae)	Parasitoid insect
Thalassa montesumae Mulsant (Coleoptera: Coccinellidae)	Predatory insect
Thaumastocoris peregrinus Carpintero & Dellapé (Hemiptera: Thaumastocoridae)	Herbivorous insect
Thaumatotibia (= *Cryptophlebia*) *leucotreta* Meyrich (Lepidoptera: Tortricidae)	Herbivorous insect
Thecla basilides (Geyer) (Lepidoptera: Lycaenidae)	Herbivorous insect
Theobroma cacao L. (Malvales: Malvaceae)	Plant
Theridula gonygaster (Simon) (Araneae: Theridiidae)	Predatory spider
Therioaphis trifolii Monell (Hemiptera: Aphididae)	Herbivorous insect
Theronia lineata (Fabricius) (Hymenoptera: Ichneumonidae)	Parasitoid insect
Theronia sp. (Hymenoptera: Ichneumonidae)	Parasitoid insect
Thersilochus argentinensis (Blanchard) (Hymenoptera: Ichneumonidae)	Parasitoid insect
Thersilochus parkeri (Blanch.) (Hymenoptera: Ichneumonidae)	Parasitoid insect
Thiara granifera (Lamarck) (Caenogastridae: Thiaridae)	Herbivorous snail
Thiara tuberculata (Muller) (Caenogastridae: Thiaridae)	Herbivorous snail
Thielaviopsis paradoxa (De Seynes) (Microascales: Ceratocystidaceae)	Phytopathogenic fungus
Thripastichus gentilei (Del Guercio) (Hymenoptera: Eulophidae)	Parasitoid insect
Thrips palmi Karny (Thysanoptera: Thripidae)	Herbivorous insect
Thrips tabaci Linderman (Thysanoptera: Thripidae)	Herbivorous insect
Thyrinteina arnobia (Stoll) (Lepidoptera: Geometridae)	Herbivorous insect
Tibraca limbativentris Stal (Hemiptera: Pentatomidae)	Herbivorous insect
Tilletia indica Mitra (Tilletiales)	Phytopathogenic fungus
Tiphia parallela Smith (Hymenoptera: Scoliidae)	Parasitoid insect
Tiphia sp. (Hymenoptera: Scoliidae)	Parasitoid insect
Tipophorus nigritus F. (Coleoptera: Chrysomelidae)	Herbivorous insect
Tomaspis aff. *pubescens* Fabr. (Homoptera: Cercopidae)	Herbivorous insect
Tomaspis saccharina Dist. (Homoptera: Cercopidae)	Herbivorous insect
Tomaspis sp. (Homoptera: Cercopidae)	Herbivorous insect
Toxomenus floralis (F.) (Diptera: Syrphidae)	Predatory insect
Toxomerus (Diptera: Syrphidae)	Predatory insects
Toxomerus watsoni (Curran) (Diptera: Syrphidae)	Predatory insect
Toxoptera (Hemiptera: Aphididae)	Herbivorous insects
Toxoptera aurantii (Boyer de Fonscolombe) (Hemiptera: Aphididae)	Herbivorous insect
Toxoptera citricidus (Kirk.)(syn. *Toxoptera citricida*) (Hemiptera: Aphididae)	Herbivorous insect
Toxoptera spp. (Hemiptera: Aphididae)	Herbivorous insects
Toxorhynchites brevipalpis (Theobald) (Diptera: Culicidae)	Predatory insect
Toxotrypana curvicauda Gerstaecker (Diptera: Tephritidae)	Herbivorous insect
Trialeurodes abutilonea (Haldeman) (Hemiptera: Aleyrodidae)	Herbivorous insect
Trialeurodes spp. (Hemiptera: Aleyrodidae)	Herbivorous insects
Trialeurodes vaporariorum Westwood (Hemiptera: Aleyrodidae)	Herbivorous insect

Triaspis sp. (Hymenoptera: Braconidae)	Parasitoid insect
Triatoma rubrofaciata (DeG.) (Hemiptera: Reduviidae)	Harmful insect
Triatoma spp. (Hemiptera: Reduviidae)	Harmful insects
Tribulus cistoides L. (Zygophyllales: Zygophyllaceae)	Plant
Tribulus terrestris L. (Zygophyllales: Zygophyllaceae)	Plant
Trichocirocalus horridus Panz. (Coleoptera: Curculionidae)	Herbivorous insect
Trichoderma (Hypocreales: Hypocreaceae)	Fungalpathogenic and antagonistic fungi
Trichoderma asperellum Samuels, Lieckf. & Nirenberg (Hypocreales: Hypocreaceae)	Fungalpathogenic and antagonistic fungus
Trichoderma atroviride (Karsten) Bissett (Hypocreales: Hypocreaceae)	Fungalpathogenic and antagonistic fungus
Trichoderma crassum Bisset (Hypocreales: Hypocreaceae)	Fungalpathogenic and antagonistic fungus
Trichoderma gamsii Samuels & Druzhin (Hypocreales: Hypocreaceae)	Fungalpathogenic and antagonistic fungus
Trichoderma hamatum (Bonord.) Bainier (Hypocreales: Hypocreaceae)	Fungalpathogenic and antagonistic fungus
Trichoderma harzianum Rifai (Hypocreales: Hypocreaceae)	Fungalpathogenic and antagonistic fungus
Trichoderma koningii Oudem (Hypocreales: Hypocreaceae)	Fungalpathogenic and antagonistic fungus
Trichoderma koningiopsis Samuels, C. Suarez & H.C. Evans (Hypocreales: Hypocreaceae)	Fungalpathogenic and antagonistic fungus
Trichoderma lignorum (Tode) Harz (Hypocreales: Hypocreaceae)	Fungalpathogenic and antagonistic fungus
Trichoderma longibrachiatum Rifai (Hypocreales: Hypocreaceae)	Fungalpathogenic and antagonistic fungus
Trichoderma martiale Samuels (Hypocreales: Hypocreaceae)	Fungalpathogenic and antagonistic fungus
Trichoderma ovalisporum Samuels & Schroers (Hypocreales: Hypocreaceae)	Fungalpathogenic and antagonistic fungus
Trichoderma pseudokoningii Rifai (Hypocreales: Hypocreaceae)	Fungalpathogenic and antagonistic fungus
Trichoderma sp. (Hypocreales: Hypocreaceae)	Fungalpathogenic and antagonistic fungus
Trichoderma spp. (Hypocreales: Hypocreaceae)	Fungalpathogenic and antagonistic fungi
Trichoderma stromaticum Samuels & Pardo-Schulth (Hypocreales: Hypocreaceae)	Fungalpathogenic and antagonistic fungus
Trichoderma virens (Miller, Giddens & Foster) von Arx (Hypocreales: Hypocreaceae)	Fungalpathogenic and antagonistic fungus
Trichoderma viride Pers. (Hypocreales: Hypocreaceae)	Fungalpathogenic and antagonistic fungus
Trichogramma (Hymenoptera: Trichogrammatidae)	Parasitoid insects
Trichogramma (*semifumatum*) *pretiosum* Riley (Hymenoptera: Trichogrammatidae)	Parasitoid insect
Trichogramma atopovirilia Oatman and Platner (Hymenoptera: Trichogrammatidae)	Parasitoid insect
Trichogramma australicum (Girult.) (Hymenoptera: Trichogrammatidae)	Parasitoid insect
Trichogramma beckeri (Nagarkatti) (Hymenoptera: Trichogrammatidae)	Parasitoid insect
Trichogramma bennetti Nagaraja & Nagarkatti (Hymenoptera: Trichogrammatidae)	Parasitoid insect
Trichogramma brasiliensis (Ashm.) (Hymenoptera: Trichogrammatidae)	Parasitoid insect
Trichogramma brassicae Bezdenko (Hymenoptera: Trichogrammatidae)	Parasitoid insect
Trichogramma bruni Nagaraja (Hymenoptera: Trichogrammatidae)	Parasitoid insect
Trichogramma cacoeciae Marchal (Hymenoptera: Trichogrammatidae)	Parasitoid insect
Trichogramma chilonis Ishii (Hymenoptera: Trichogrammatidae)	Parasitoid insect
Trichogramma dendrolimi Matsumura (Hymenoptera: Trichogrammatidae)	Parasitoid insect

Trichogramma diazi Velásquez and Terán (Hymenoptera: Trichogrammatidae)	Parasitoid insect
Trichogramma euproctidis (Girault) (Hymenoptera: Trichogrammatidae)	Parasitoid insect
Trichogramma evanescens Westwood (Hymenoptera: Trichogrammatidae)	Parasitoid insect
Trichogramma exiguum Pinto & Platner (Hymenoptera: Trichogrammatidae)	Parasitoid insect
*Trichogramma fasciatum (*Perkins) (= *minutum* auct.) (Hymenoptera:Trichogrammatidae)	Parasitoid insect
Trichogramma fasciatum (Perkins) (Hymenoptera: Trichogrammatidae)	Parasitoid insect
Trichogramma fuentesi Torre (Hymenoptera: Trichogrammatidae)	Parasitoid insect
Trichogramma galloi Zucchi (Hymenoptera: Trichogrammatidae)	Parasitoid insect
Trichogramma lasallei Pinto (Hymenoptera: Trichogrammatidae)	Parasitoid insect
Trichogramma lopezandinensis Sarmiento (Hymenoptera: Trichogrammatidae)	Parasitoid insect
Trichogramma marandobai Brun, Moraes e Soares (Hymenoptera: Trichogrammatidae)	Parasitoid insect
Trichogramma minutum Riley (Hymenoptera: Trichogrammatidae)	Parasitoid insect
Trichogramma near *pretiosum* (Hymenoptera: Trichogrammatidae)	Parasitoid insect
*Trichogramma nerudai (*Pintureau & Gerding) (Hymenoptera: Trichogrammatidae)	Parasitoid insect
Trichogramma nubilale Ertie & Davis (Hymenoptera: Trichogrammatidae)	Parasitoid insect
Trichogramma obscurum Pinto (Hymenoptera: Trichogrammatidae)	Parasitoid insect
Trichogramma perkinsi Girault (Hymenoptera: Trichogrammatidae)	Parasitoid insect
Trichogramma pintoi Voegelé (Hymenoptera: Trichogrammatidae)	Parasitoid insect
Trichogramma platneri Nagarkatti (Hymenoptera: Trichogrammatidae)	Parasitoid insect
Trichogramma pretiosum Riley (Hymenoptera: Trichogrammatidae)	Parasitoid insect
Trichogramma rojasi Nagaraja & Nagarkatti (Hymenoptera: Trichogrammatidae)	Parasitoid insect
Trichogramma semifumatum (Perkins) (Hymenoptera: Trichogrammatidae)	Parasitoid insect
Trichogramma sp. (Hymenoptera: Trichogrammatidae)	Parasitoid insect
Trichogramma sp1 (Hymenoptera: Trichogrammatidae)	Parasitoid insect
Trichogramma sp2 (Hymenoptera: Trichogrammatidae)	Parasitoid insect
Trichogramma spp. (Hymenoptera: Trichogrammatidae)	Parasitoid insects
Trichogramma terani Velásquez and Terán (Hymenoptera: Trichogrammatidae)	Parasitoid insect
Trichogrammatoidea armigera (Nagaraja) (Hymenoptera: Trichogrammatidae)	Parasitoid insect
Trichogrammatoidea bactrae Nagaraja (Hymenoptera: Trichogrammatidae)	Parasitoid insect
Trichogrammatoidea cryptophlebiae Nagaraja (Hymenoptera: Trichogrammatidae)	Parasitoid insect
Trichogrammatoidea robusta Nagaraja *(*as *T. nana* Zhnt.*)* (Hymenoptera: Trichogrammatidae)	Parasitoid insect
Trichogrammatoidea sp. (Hymenoptera: Trichogrammatidae)	Parasitoid insect
Trichomalopsis Crawford (Hymenoptera: Pteromalidae)	Parasitoid insect
Trichoplusia ni (Hubner) (Lepidoptera: Noctuidae)	Herbivorous insect
Trichoplusia ni nucleopolyhedrovirus (TnNPV)	Entomopathogenic virus
Trichoplusia sp. (Lepidoptera: Noctuidae)	Herbivorous insect
Trichopoda giacomelli (Blanchard) (Diptera: Tachinidae)	Parasitoid insect
Trichopoda pennipes (F.) (Diptera: Tachinidae)	Parasitoid insect
Trichopoda pilipes F. (Diptera: Tachinidae)	Parasitoid insect
Trichopoda sp. (Diptera: Tachinidae)	Parasitoid insect
Trichopria drosophilae (Hymenoptera: Diapriidae)	Parasitoid insect
Trichospilus diatraeae (C. and M.) (Hymenoptera: Eulophidae)	Parasitoid insect
Trichospilus pupivora (= *pupivorus*) Ferriere (Hymenoptera: Eulophidae)	Parasitoid insect
Trioxys pallidus (Haliday) (Hymenoptera: Braconidae)	Parasitoid insect
Trissolcus (Hymenoptera: Platigastridae)	Parasitoid insects
Trissolcus basalis (Wollaston) (Hymenoptera: Platygastridae)	Parasitoid insect

Trissolcus brochymenae Ashmead (Hymenoptera: Platygastridae)	Parasitoid insect
Trissolcus leviventris (Cameron) (=*Dissolcus paraguayensis* Brethes) (Hymenoptera: Platygastridae)	Parasitoid insect
Trissolcus mitsukurii (Ashm.) (Hymenoptera: Platigastridae)	Parasitoid insect
Trissolcus sp. (Hymenoptera: Platygastridae)	Parasitoid insect
Trissolcus spp. (Hymenoptera: Platygastridae)	Parasitoid insects
Trissolcus teretis Johnson (Hymenoptera: Platygastridae)	Parasitoid insect
Trissolcus urichi Crawford (Hymenoptera: Platygastridae)	Parasitoid insect
Triticum aestivum (L.) (Poales: Poaceae)	Plant
Trogoderma granarium (Everts) (Coleoptera: Dermestidae)	Herbivorous insect
Trox suberosus F. (Coleoptera: Trogidae)	Saprophagous insect
*Trybliographa dac*i Weld (Hymenoptera: Cynipidae)	Parasitoid insect
Trypanosoma cruzi Chagas (Trypanosomatidae)	Harmful protozoan
Tsukamurella paurometabola (Steinhaus) (= *Corynebacterium)* (Corynebacteriales:Tsukamurellaceae)	Nematopathogenic bacterium
Tucumania tapiacola Dyar (Lepidoptera: Pyralidae)	Herbivorous insect
Tupiocoris notatus (Distant) (Hemiptera: Miridae)	Predatory insect
Tuta absoluta (Meyrick) (Lepidoptera: Gelechiidae)	Herbivorous insect
Typha domingensis (Pers.) (Poales: Thyphaceae)	Plant
Typhlodromina (Acari: Phytoseiidae)	Predatory mites
Typhlodromips (= *Amblyseius*) *swirskii* (Athias-Henriot) (Acari: Phytoseiidae)	Predatory mite
Typhlodromus citri Garman & McG (Acari: Phytoseiidae)	Predatory mite
Typhlodromus occidentalis (= *Galandromus*) Nesbitt (Acari: Phytoseiidae)	Predatory mite
Typhloseiopsis adventitius Ferragut & Moraes sp. Nov (Acari: Phytoseiidae)	Predatory mite
Tytthus mundulus (Breddin) (Hemiptera: Miridae)	Predatory mirid
Tytthus parviceps Reuter (Hemiptera: Miridae)	Predatory mirid
U	
Ulex europaeus L. (Fabales Fabaceae)	Plant
Unaspis citri (Comstock) (Hemiptera: Diaspididae)	Herbivorous insect
Uredo eichhorniae Fragoso and Ciferri (Pucciniales)	Phytopathogenic fungus
Urena lobata L. (Malvales:Malvaceae)	Plant
Urena trilobata Vell. (Malvales: Malvaceae)	Plant
Uromyces pencanus (Diet. & Neg.) (Puciniales: Pucciniaceae)	Phytopathogenic fungus
Urosigalphus eulechriopis Cushman (Hymenoptera: Braconidae)	Parasitoid insect
Urtica urens L. (Urticales: Urticaceae)	Plant
Ustilago scitaminea Sydow (Ustilaginales)	Phytopathogenic fungus
Utetes anastrephae (Viereck) (Hymenoptera: Braconidae)	Parasitoid insect
Utetheisa ornatrix (L.) (Lepidoptera: Erebidae)	Herbivorous insect
V	
Varroa destructor Anderson & Truemann (Parasitiformes: Varroidae)	Harmful mite
Vehilius celeus Mab. (Lepidoptera: Hesperiidae)	Herbivorous insect
*Venturia (*Hymenoptera: Ichneumonidae)	Parasitoid insects
Venturia ovivenans nov. spec Zwart (Hymenoptera: Ichneumonidae)	Parasitoid insect
Venturia sp. (Hymenoptera: Ichneumonidae)	Parasitoid insect
*Verticillium (*Hypocreales: Plectosphaerellaceae)	Entomopathogenic fungus
Verticillium sp. (Hypocreales: Plectosphaerellaceae)	Entomopathogenic fungus

Vigna sesquipedalis (L.) (Fabales: Fabaceae)	Plant
Vincentodiplosis pseudococci (Felt) (Diptera: Cecidomyiidae)	Predatory insect
Vinsonia stellifera (Westw.) (Hemiptera: Coccidae)	Herbivorous insect
Vitis syciodes L. (Vitales:Vitaceae)	Plant
Voria sp. (Diptera: Tachinidae)	Parasitoid insect
Voria spp. (Diptera: Tachinidae)	Parasitoid insects
VPNHz (*Helicoverpa zea* virus)	Entomopathogenic virus
VPNSe *(Spodoptera exigua* virus)	Entomopathogenic virus
VPNSf *(Spodoptera frugiperda* virus*)*	Entomopathogenic virus
VPNSs *(Spodoptera sunia virus)*	Entomopathogenic virus
VPNTni *(Trichoplusia nii virus)*	Entomopathogenic virus
W	
Wasmania auropunctata Roger (Hymenoptera: Formicidae)	Predatory insect
Wasmannia rochai Forel (Hymenoptera: Formicidae)	Predatory insect
Winthemia pinguioides (Townsend) (Diptera: Tachinidae)	Parasitoid insect
Winthemia pinguis (Fabricius) (Diptera: Tachinidae)	Parasitoid insect
Winthemia sp. (Diptera: Tachinidae)	Parasitoid insect
Winthemia sp. nr. *pinguis* F. (Diptera: Tachinidae)	Parasitoid insect
Winthemia sp. similar to *W. pyrrhopyga* (Wied) (Diptera: Tachinidae)	Parasitoid insect
Winthemia spp. (Diptera: Tachinidae)	Parasitoid insects
X	
Xanthogaleruca luteola (Muller) (Coleoptera: Chrysomelidae)	Herbivorous insect
Xanthomonas campestris (Pammel) Dowson (Xanthomonadales: Xanthomonadaceae)	Phytopathogenic bacterium
Xanthomonas campestris pv. *Musacearum (*Yirgou & Braddury) (Xanthomonadales: Xanthomonadaceae)	Phytopathogenic bacterium
Xanthomonas citri (Hasse) Gabriel et al. (Xanthomonadales: Xanthomonadaceae))	Phytopathogenic bacterium
*Xanthomonas phaseoli (*Smith) Gabriel et al. (Xanthomonales: Xanthomonadaceae)	Phytopathogenic bacterium
Xanthomonas vesicatoria (Doidge) Dowson (Xanthomonadales: Xanthomonadaceae)	Phytopathogenic bacterium
Xanthopygus cognatus Sharp (Coleoptera: Staphylinidae)	Predatory insect
Xenoencyrtus niger Riek (Hymenoptera: Encyrtidae)	Parasitoid insect
Xenostigmus bifasciatus Ashmed (Hymenoptera: Braconidae)	Parasitoid insect
Xiphinema (Dorylaimida: Longidoridae)	Phytopathogenic nematodes
Xubida infusella (Walker) (Lepidoptera: Pyralidae)	Herbivorous insect
Xyleborus glabratus (Eichhoff) (Coleoptera: Curculionidae)	Herbivorous insect
Xylella fastidiosa sp. *multiplex* (Xanthomonadales: Xanthomonadaceae)	Phytopathogenic bacterium
Xylella fastidiosa subsp. *fastidiosa* Well. (Xanthomonadales: Xanthomonadaceae))	Phytopathogenic bacterium
Xylella fastidiosa subsp. *pauca* Schaad, Postnikova, Lacy, Fatmic & Chang ((Xanthomonadales: Xanthomonadaceae))	Phytopathogenic bacterium
Xylocopa sp. (Hymenoptera: Apidae)	Herbivorous insect
Xylocoris flavipes (Reuter) (Hemiptera: Anthocoridae)	Predatory insect
Xylosandrus compactus Eichoff (Coleoptera: Scolytidae)	Herbivorous insect
Z	
Zachrisia auricoma (Ferussac) (Mollusca: Gastropoda: Pleurodontidae)	Herbivorous snail
Zaeucoila sp. (Hymenoptera: Figitidae)	Parasitoid insect
Zagloba aenipennis (Sicard) (Coleoptera: Coccinellidae)	Predatory insect

Zaglyptus Forster (Hymenoptera: Ichneumonidae)	Parasitoid insects
Zagrammosoma multilineata Ashm (Hymenoptera: Eulophidae)	Parasitoid insect
Zagrammosoma sp. (Hymenoptera: Eulophidae)	Parasitoid insect
Zagrammosoma spp. (Hymenoptera: Eulophidae)	Parasitoid insects
Zagreus (= Exochomus) *bimaculosus* Mulsant (Coleoptera: Coccinellidae)	Predatory insect
Zea mays L. (Poales: Poaceae)	Plant
Zelomorpha sp. (Hymenoptera:Braconidae)	Parasitoid insect
Zelus argillaceum (L.) (Hemiptera: Reduviidae)	Predatory insect
Zelus armillatus (Lepeletier and Serville) (Hemiptera: Reduviidae)	Predatory insect
Zelus cf. *nugax* Stål (Hemiptera: Reduviidae)	Predatory insect
Zelus illotus Berg (Hemiptera: Reduviidae)	Predatory insect
Zelus laticornis (Herrich-Schäffer) (Hemiptera: Reduviidae)	Predatory insect
Zelus leucogrammus (Perty) (Hemiptera: Reduviidae)	Predatory insect
Zelus longipes (L.) (Hemiptera: Reduviidae)	Predatory insect
Zelus ruficeps Stål (Hemiptera: Reduviidae)	Predatory insect
Zelus sp. (Hemiptera: Reduviidae)	Predatory insect
Zelus spp. (Hemiptera: Reduviidae)	Predatory insects
Zenoria emarginata Gordon (Coleoptera: Coccinellidae)	Predatory insect
Zeta argillaceum (L.) (Hymenoptera: Vespidae)	Predatory insect
Zetesima baliandra (Meyr.) (Lepidoptera: Stenomidae)	Herbivorous insect
Zulia entreriana (Berg. (Hemiptera: Cercopidae)	Herbivorous insect
Zulia spp. (Hemiptera: Cercopidae)	Herbivorous insects
