# Supplementary material for: Benefits and Risks of Native and Exotic Biological Control Agents Used in Latin America and the Caribbean: Performance of 1099 Arthropod Natural Enemies
Source: Neotrop Entomol. 2026 Jul 28;55(1):69. doi: 10.1007/s13744-026-01412-8 (PMC13415494; doi:10.1007/s13744-026-01412-8)
Supplement: Supplementary file 2 — (PDF 568 KB) [file 13744_2026_1412_MOESM2_ESM.pdf]

**Table SI2. Benefits and risks of endemic and exotic biological control agents used in Latin America and the Caribbean: performance of 1099 arthropod natural enemies. Joop C. van Lenteren, Vanda H. P. Bueno, Wageningen University, Laboratory of Entomology, Department of Plant Sciences, 6700 AA, Wageningen, The Netherlands, joop.vanlenteren@wur.nl**<br><br>**Parasitoids mentioned in van Lenteren et al. 2020: "Biological Control In Latin America and The Caribbean: Its Rich History And Bright Future"**	
**Parasitoid species**	**Country where recorded, endemic/exotic, role in biocontrol**
Acaulona erythropyga Sabrosky (Diptera: Tachinidae)	RC, endemic, role not quantified, NC
Acaulona peruviana Tns (Diptera: Tachinidae)	RC, exotic, not established
Aceratoneuromyia (=*Synthomosphyrum indicum*) *indica* Silvestri (Hymenoptera: Eulophidae)	AR, exotic, not established, BZ, exotic, established, insuffic control, CR, DM, exotic, establ, insuff control, MX, exotic, established, reduces pest, CBC, RC, exotic, establ?
Acerophagous papayae Noyes & Schauff (Hymenoptera: Encyrtidae)	BB, JM, RC, exotic, established, controlled pest, CBC
Acerophagus coccois Smith (Hymenoptera: Encyrtidae)	BR, exotic, established, good control, CBC
Acerophagus spp. (Hymenoptera: Encyrtidae)	DO, PR, RC, exotic, established, controlled pest, CBC
Achrysochari sp. (Hymenoptera: Eulophidae)	VE, endemic, role not quantified, NC
Adelencyrtus moderatus (How.) (Hymenoptera: Encyrtidae)	BB, endemic, role not quantified, NC
Adelencyrtus odonaspidis Fullaway (Hymenoptera: Encyrtidae)	BB, endemic, role not quantified, NC
Aenasius vexans (Kerrich) (Hymenoptera: Encyrtidae)	BR, exotic, controls pest, CBC
Aganaspis daci (Weld) (Hymenoptera: Eulophidae)	CR, MX, exotic, established, controls pest, CBC
Aganaspis pelleranoi (Brethes) (Hymenoptera: Eulophidae)	SR, endemic, role not quantified, NC
Agathis diversa (Mues.) (Hymenoptera: Braconidae)	AR, exotic, established, controls pest, CBC
Agathis sp. (Hymenoptera: Braconidae)	BB, exotic, not established, CBC
Agathis stigmatera (Cresson) (Hymenoptera: Braconidae)	AR, endemic, not quantified, NC, BB, exotic, established, insuff control, CBC, RC, exotic, establ?, SR, endemic, insuff control, NC, TT, endemic, reduces pest, NC
Ageniaspis citricola (Logvinovskaya) (Hymenoptera: Encyrtidae)	AR, BB, BR, EC, FA, MX, PE, RC, UY, exotic, established, controls pest, CBC, ABC
Agrothereuthes diatraeae Myers (Hymenoptera: Ichneumonidae)	VE, endemic, not quantified, NC
Alabagrus stigma Brullé (Hymenoptera: Braconidae)	GY, exotic, established, role not quantified, CBC

Aleiodes sp. (Hymenoptera: Braconidae)	PY, endemic, not quantified, NC
Aleuroctonus vittatus (Dozier) (Hymenoptera: Eulophidae)	FA endemic, not quantified, NC
Allorhogas pyralophagus (Marsh) (Hymenoptera: Braconidae)	GY, exotic, established, insuff control, CBC
Allorhogas sp. (Hymenoptera: Braconidae)	RC, exotic, establ ?, not quantified, CBC
Allotropa citri (Muesebeck) (Hymenoptera: Platigasteridae)	CL, exotic, no further info, CBC
Allotropa sp. (Hymenoptera: Platygasteridae)	DO, endemic, reduces pest, NC, RC, endemic, not quant, NC
Amitus bennetti Viggiani & Evans (Hymenoptera: Platygasteridae)	FA, endemic, not quantified, NC
Amitus fuscipennis (MacGown & Nebeker) (Hymenoptera: Platygastridae)	CO, DO, FA, endemic, reduces pest, ABC, NC
Amitus hesperidum Silvestre (Hymenoptera: Platygastridae)	DM, EC, GT, MX, PR, RC, TT, exotic, established, good control. CBC
Amitus sp. (Hymenoptera: Platigastridae)	PA, endemic, not quantified, NC
Anagrus flaveolus Waterhouse (Hymenoptera: Mymaridae)	BZ, endemic, reduces pest, NI, JM, RC, exotic, established, insuff control, CBC, TT, VE, status unclear
Anagrus gonzalezae Triapitsyn (Hymenoptera: Mymaridae)	HN, endemic, reduces pest, ConsBC
Anagrus optabilis (Perkins) (Hymenoptera: Mymaridae)	EC, endemic, reduces pest, ABC, CBC
Anagrus urichi Pickles (Hymenoptera: Mymaridae)	TT, endemic, not quantified, NC
Anagyrus (=Apoanagyrus) californicus (Compere) (Hymenoptera: Encyrtidae)	DO, RC, exotic, established, reduced pest, CBC
*Anagyrus diversicornis (*Howard) (Hymenoptera: Encyrtidae)	BR, exotic, established, good control, CBC
Anagyrus kamali Moursi (Hymenoptera: Encyrtidae)	BB, BZ, DO, FA, GY, HT, JM, MX, PR, RC, SR, TT, VE exotic, reduces pest, CBC
Anagyrus loecki Noyes and Menezes (Hymenoptera: Encyrtidae)	BB, DO, JM, RC, exotic, established, reduced pest, CBC
Anagyrus mangicola Noyes (Hymenoptera: Encyrtidae)	FA, exotic, is going to be introduced, no results yet, CBC
Anagyrus pseudococci (Girault) (Hymenoptera: Encyrtidae)	CL, MX, PE: exotic, established, reduces pest, ABC
Anagyrus saccharicola Timb. (Hymenoptera: Encyrtidae)	BB, BO, PE, RC, exotic, established, reduces pest, CBC
Anagyrus sp. (Hymenoptera: Encyrtidae)	JM, PE, exotic, established, some control, CBC
Anagyrus spp. (Hymenoptera: Encyrtidae)	PR, RC, exotic, established, good control, CBC
Anaphes iole Girault (Hymenoptera: Mymaridae)	MX, exotic, no further information
Anaphes nitens Girault (Hymenoptera: Mymaridae)	BR, CL, UY: exotic, established, reduces pest, CBC, ABC
Anastatus sp. (Hymenoptera: Eupelmidae)	SR, endemic, reduces pest, NC
Anastatus spp. (Hymenoptera: Eupelmidae)	RC, exotic, not established
Anicetus sp. (Hymenoptera: Encyrtidae)	BB, exotic, no further info, not known if establ
Anisopteromalus calandrae (Howard) (Hymenoptera: Pteromalidae)	MX, exotic, no further info
Antrocephalus renalis Wtstn (Hymenoptera: Chalcididae)	BZ, exotic, not established

Apanteles (Rhygoplitis) aciculatus (Ashm) (Hymenoptera: Braconidae)	RC, endemic, reduces pest, NC
Apanteles angeleti Mues. (Hymenoptera: Braconidae)	BB, RC, exotic, not established
Apanteles etiellae Viereck (Hymenoptera: Braconidae)	BB, exotic, not established, TT endemic, no further info, NC
Apanteles gelechiidivoris Marsh (Hymenoptera: Braconidae)	CO, endemic, reduces pest, ABC
Apanteles sesamiae Cam. (Hymenoptera: Braconidae)	BO, RC exotic, not established
Apanteles sp. (Hymenoptera: Braconidae)	BB, BO, CO, CR, EC, FA, PA, PR, RC, exotic, no success, not established SR endemic, from no control to reduces pest, NC, ABC;
Apanteles spp. (Hymenoptera:Braconidae)	CR, PY, UY endemic, role not quantified, NC
Apanteles talidicica Wlkn (Hymenoptera: Braconidae)	RC, exotic, not established
Apanteles thurberiae Muesebeck (Hymenoptera: Braconidae)	CO, endemic, reduces pest, NC
Aphanogmus sp. (Hymenoptera: Ceraphronidae)	DO endemic, role not quantified, NC
Aphelinus abdominalis Dalman (Hymenoptera: Aphelinidae)	BR, MX, exotic, reduces pest, ABC
Aphelinus albipodus (Hayat and Fatima) (Hymenoptera: Aphelinidae)	PY, endemic, role not quantified, NC
Aphelinus asychis (Walker) (Hymenoptera: Aphelinidae)	BR, exotic, established, reduces pest, ABC, CBC
Aphelinus flavipes Forster (Hymenoptera: Aphelinidae)	BR, exotic, established, reduces pest, CBC
Aphelinus gossypii (Timberlake) (Hymenoptera: Aphelinidae)	FA, PY, endemic, no further info, NC
Aphelinus mali Haldeman (Hymenoptera: Aphelinidae)	AR, BO, BR, CL, CO, CR, EC, PE, UY, VE exotic, established, reduces pest, CBC
Aphelinus varipes Forster (Hymenoptera: Aphelinidae)	BR, exotic, established, reduces pest, CBC
Aphidius colemani Viereck (Hymenoptera: Braconidae)	AR, BR, CO, CR, DO, FA, MX, VE, exotic, established, reduces pest, ABC
Aphidius ervi Haliday (Hymenoptera: Braconidae)	AR, BR, MX exotic, established, reduces pest, ABC, CBC
Aphidius matricariae Haliday (Hymenoptera: Braconidae)	CO, MX, exotic, reduces pest, ABC
Aphidius pascuorum Marshall (Hymenoptera: Braconidae)	BR, exotic, established, reduces pest, CBC
Aphidius picipes (Ness) (Hymenoptera: Braconidae)	BR exotic, established, reduces pest, CBC
Aphidius rhopalosiphi De Stefani (Hymenoptera: Braconidae)	AR, BR exotic, established, reduces pest, ABC
Aphidius smithi Sharma & Subba Rao (Hymenoptera: Braconidae)	AR, PE exotic, established, reduces pest, CBC
Aphidius spp. (Hymenoptera: Braconidae)	PY, endemic, not quantified, NC
Aphidius uzbekistanicus Luzhetzki (Hymenoptera: Braconidae)	AR, BR, exotic, established, reduces pest, CBC
Aphytis chrysomphali Mercet (Hymenoptera: Aphelinidae)	MX, SR exotic, established, reduces pest, CBC
Aphytis diaspidis (How) (Hymenoptera: Aphelinidae)	JM, PE, exotic, established, reduces pest, CBC
Aphytis fuscipennis (How) (Hymenoptera: Aphelinidae)	CO, DO, FA,PE endemic, reduces pest, NC, ABC

Aphytis holoxanthus DeBach (Hymenoptera: Aphelinidae)	AR, MX, exotic, established, reduces pest, CBC
Aphytis lepidosaphes Compere (Hymenoptera: Aphelinidae)	AR, EC, SV, MX, PE, exotic, established, reduces pest, CBC
Aphytis lingnanensis (Howard) (Hymenoptera, Aphelinidae)	AR, MX, exotic, established, reduces pest, CBC
Aphytis maculicornis (Masi) (Hymenoptera: Aphelinidae)	AR, exotic, not established
Aphytis melinus DeBach (Hymenoptera: Aphelinidae)	AR, MX, PE, exotic, established, reduces pest, CBC
Aphytis mytilaspidis (Le Baron) (Hymenoptera: Aphelinidae)	AR, exotic, established, role not quantified, CBC
Aphytis roseni DeBach (Hymenoptera: Aphelinidae)	PE, exotic, established, good control, CBC
Aphytis sp. (Hymenoptera: Aphelinidae)	FA, exotic, established, reduces pest, CBC
Aphytis yanonensis DeBach & Rosen (Hymenoptera: Aphelinidae)	AR exotic, established, role not quantified, CBC
Apoanagyrus diversicornis (Howard) (Hymenoptera: Encyrtidae)	BR exotic, established, reduces pest, CBC
Apoanagyrus sp. (Hymenoptera: Encyrtidae)	JM, endemic, reduces pest, NC
Apoanagyrus spp. (Hymenoptera: Encyrtidae)	RC endemic?, reduces pest, NC
Aprostocetus (Hymenoptera: Eulophidae)	DO, endemic, role not quantified, NC
Aprostocetus gala (Walker) (Hymnoptera: Eulophidae)	FA, JM endemic, reduces pest, NC
Aprostocetus haitiensis (Gahan) (Hymnoptera: Eulophidae)	FA, JM, endemic, reduces pest, NC
Aprostocetus sp. (Hymenoptera: Eulophidae)	FA endemic, role unknown, NC
Archytas divisa (Walk.) Diptera: Tachinidae)	SR endemic, role unknown, NC
Archytas marmoratus (Tns.) (Diptera: Tachinidae)	BB, BO, CU, SR, VE, endemic, some control, NC
Archytas sp. (Diptera: Tachinidae)	PY, endemic, role not quantified, NC
Archytas vernalis Curran (Diptera: Tachinidae)	SR endemic, role not quantified, NC
Ardalus scutellatus (How.) (Hymenoptera: Eulophidae)	BB, RC, exotic, not established
Arrhenophagus chionaspidis Auriv. (Hymenoptera: Encyrtidae)	PE, exotic, established, reduces pest, CBC
Ascogaster (Hymenoptera: Braconidae)	CL, CR exotic, established, no control, CBC
Ascogaster quadridentata Wesmael (Hymenoptera: Braconidae)	AR, CL, UY, exotic, established, no control to reduces pest, CBC
Aspidiotiphagus citrinus (Crwf) (Hymenoptera: Aphelinidae)	PE, exotic, established, some control, CBC
Aspidiotiphagus sp. (Hymenoptera: Aphelinidae)	RC, exotic, established, role not quantified, CBC
Avetianella longoi Siscaro (Hymenoptera: Encyrtidae)	UY, exotic, established, reduces pest, CBC
Aximopsis sp. (Hymenoptera: Eurytomidae)	PY, endemic, role not quantified, NC
Baryscapus (Hymenoptera: Eulophidae)	DO endemic, role not quantified, NC
Baryscapus fennahi (Schauff) (Hymenoptera: Eulophidae)	FA endemic, role not quantified, NC
Bassus sp. (Hymenoptera: Braconidae)	CR endemic, role not quantified, NC

Bassus stigmaterus Holloway (Hymenoptera: Braconidae)	PR, exotic, established, some control, CBC
Belvosia nigrifrons Aldrich (Diptera: Tachinidae)	SV, endemic, reduces pest, NC
Billaea (Paratheresia) claripalpis Wulp (Diptera: Tachinidae)	AR, BB, BO, BR, CO, CR, CU, DM, EC, FA, JM, PA, PE, RC, TT, VE, endemic and exotic, established, no control to reduces pest, ABC
Biosteres compensans (Silvestri) (= Opius compensans Silvestri) (Hymenoptera: Braconidae)	BO, CR, exotic, reduces pest, ABC
Biosteres formasanus (Fullaway) (= *Opius formosanus* Fullaway) (Hymenoptera: Braconidae)	BO, CR exotic, reduces pest, ABC
Biosteres longicaudatus (Ashmead) (= *Opius longicaudatus*) (Hymenoptera: Braconidae)	BO, exotic, reduces pest, ABC, BZ, CR, EC, DM, RC, exotic, establish unknown, insufficient control, CBC
Biosteres novocaledonicus Fullaway (Hymenoptera: Bracondiae)	CR, exotic, establ unkown, role not quantified
Biosteres taiensis Fullaway (Hymenoptera:Bracondiae)	CR exotic, establ unkown, role not quantified
Biosteres tryoni (Cameron) (= *Opius tryoni* Cameron) (Hymenoptera: Braconidae)	CR exotic, establ unkown, role not quantified
Biosteres vandenboschi (Fullaway) (= *Opius vandenboschi* Fullaway) (Hymenoptera: Braconidae)	BO, exotic, reduces pest, ABC, CR exotic, role not quantified
Brachycoryphus nursei (Cam.) (Hymenoptera: Ichneumonidae)	RC, exotic, not established
Brachymeria (Hymenoptera: Chalcididae)	CR, DO, endemic, reduces pest, NC
Brachymeria aff. *compsilurae* (Crawford) (Hymenoptera: Chalcididae)	PY, endemic, role not quantified, NC
Brachymeria annulata (Fabricius) (Hymenoptera: Chalcididae)	PY endemic, role not quantified, NC
Brachymeria conica (Ashmead) (Hymenoptera:Chalcididae)	CR endemic, reduces pest, NC
Brachymeria incerta (Cress) (Hymenoptera: Chalcididae)	SR, VE endemic, reduces pest, NC
Brachymeria ovata (Say) (Hymenoptera: Chalcididae)	VE endemic, role not quantified, NC
Brachymeria sp. (Hymenoptera: Chalcididae)	BB, FA, JM, RC, SR endemic, reduces pest, NC
Brachymeria subconica Bouček (Hymenoptera: Chalcididae)	PY endemic, role not quantified, NC
Brachyufens osborni (Dozier) (Hymenoptera: Trichogrammatidae)	BB exotic, not established
Brachyufens sp. (Hymenoptera:Trichogrammatidae)	JM exotic, all arrived dead
Bracon cajani Muesebeck (Hymenoptera: Braconidae)	BB, exotic, not established, PR exotic, established, reduces pest CBC
Bracon chinensis (Szepl.) (Hymenoptera: Braconidae)	BO exotic, not known if established, role not quantified, CBC
Bracon chontalensis (Cameron) (Hymenoptera:Braconidae)	CR endemic, reduces pest, NC
Bracon gelechiae Ashmead (Hymenoptera: Braconidae)	BB, exotic, established, role not quantified, CBC
Bracon greeni Ashmead (Hymenoptera: Braconidae)	BB, RC, exotic, establish unknown, role not quantified, CBC
Bracon hebetor Say (Hymenoptera: Braconidae)	BO, RC endemic, role negligible, NC
Bracon kirkpatricki (Wilkinson) (Hymenoptera: Braconidae)	CO, EC, MX, exotic, establish unknown to yes, insuf control, ABC
Bracon mellitor Say (Hymenoptera: Braconidae)	PY endemic, role not quantified, NC
Bracon sp. (Hymenoptera: Braconidae)	GT, PY, endemic, reduces pest, NC

Bracon thurberiphagae (Mues.) (Hymenoptera: Braconidae)	BB, exotic, not established
Brethesiella abnormicornis (Girault) (Hymenoptera: Encyrtidae)	BB exotic, not established
Brethesiella cf. abnormicornis (Girault) (Hymenoptera: Encyrtidae)	CO, endemic, role not quantified, NC
Cales noacki Howard (Hymenoptera: Aphelinidae)	EC, PE, exotic, established, reduces pest, CBC
Campletis chlorideae Uchida (Hymenoptera: Ichneumonidae)	BO, exotic, no further info
Campoletis spp. (Hymenoptera: Ichneumonidae).	PY, UY, endemic, role not quantified, NC
Campsomeris servillei (Guérin-Menéville) (Hymenoptera: Scoliidae)	VE, endemic, role not quantified, NC
Campsomeris tricincta F. (Hymenoptera: Scoliidae)	BB, exotic, not established
Campsomeris trifasciata (F.) (Hymenoptera: Scoliidae)	BB exotic, not established
Carabunia waterstoni Subba Rao (Hymenoptera: Encyrtidae)	TT, exotic, insufficient, not established
Cardiochiles diaphaniae Marsh (Hymenoptera: Braconidae)	PR, exotic, established, reduces pest, CBC
Cardiochiles nigriceps Viereck (Hymenoptera: Braconidae)	VE, endemic, role not quantified, NC
Carinodes sp. (Hymenoptera: Ichneumonidae)	BO, endemic, role not quantified, NC
Catolaccus grandis (Burks) (Hymenoptera: Pteromalidae)	CO, exotic, insufficient, ABC, MX, endemic, reduced pest, ABC, PY occurs, origin unknown
Cephalonomia stephanoderis Betrem (Hymenoptera: Eulophidae)	BO, CO, CR, CU, DO, EC, GT, HT, JM, MX, PA, PR exotic, established, reduces pest, CBC, ABC
Ceratogramma etiennei Delvare (Hymenoptera: Trichogrammatidae)	DO, FA, exotic, established, reduces pest, CBC
Chelonus sp. (Hymenoptera: Braconidae)	DO, PY, endemic, role not quantified, NC
Chelonus insularis Cresson (Hymenoptera: Braconidae)	CU, DO, JM, VE, endemic, role not quantified, NC
Chistolia sp. (Hymenoptera: Ichneumonidae)	SR, endemic, role not quantified, NC
Chrysocharis caribea Boucek (Hymenoptera: Eulophidae)	FA endemic, role not quantified, NC
Chrysocharis sp. (Hymenoptera: Eulophidae)	BB, PA, exotic, not established
Chrysocharis vovones (Walker) (Hymenoptera: Eulophidae)	FA endemic, role not quantified, NC
Chrysonotomyia diastatae (Howard) (Hymenoptera: Eulophidae)	HN endemic, role not quantified, NC
Cirrospilus sp. (Hymenoptera: Eulophidae)	FA, endemic, role not quantified, NC
Cirrospilus Westwood (Hymenoptera: Eulophidae)	BB, endemic, role not quantified, NC
Cirrospilus quadristriatus (Subba Rao and Ramamani) (Hymenoptera: Eulophidae)	DO, exotic, established, reduces pest, CBC
Citrostichus phyllocnistoides (Narayanan) (Hymenoptera: Eulophidae)	PE, UY, exotic, established reduces pest, CBC
Cleruchoides noackae Lin & Huber (Hymenoptera: Mymaridae)	AR, BR, UY, exotic, established, reduces pest, CBC
Closterocerus purpureus (Howard) (Hymenoptera: Eulophidae)	FA, endemic, role not quantified, NC
Coccidoxenoides peregrinus (Timberlake) (Hymenoptera: Encyrtidae)	CR, PE, endemic, reduces pest, NC

Coccidoxenoides perminutus Girault (Hymenoptera:Encyrtidae)	MX, exotic, no further info
Coccobius fulvus (Compere and Annecke) (Hymenoptera: Aphelinidae)	BB, CR, exotic, established, reduces pest CBC
Coccophagoides utilis Doutt (Hymenoptera: Aphelinidae)	AR, exotic, not established
Coccophagus aleurodici Gir. (Hymenoptera: Aphelinidae)	RC, exotic, no further info
Coccophagus basalis Compere (Hymenoptera: Aphelinidae)	FA, endemic, role not quantified, NC
Coccophagus caridei (Bréthes) (Hymenoptera: Aphelinidae)	AR, CL, exotic, reduces pest, CBC
Coccophagus gurneyi (Compere) (Hymenoptera: Aphelinidae)	CL exotic, reduces pest, CBC
Coccophagus lycimnia (Walker) (Hymenoptera: Aphelinidae)	AR exotic, no further info
Coccophagus pulvinariae Compere (Hymenoptera: Aphelinidae)	FA endemic, role not quantified, NC
Coccophagus rusti Compere (Hymenoptera: Aphelinidae)	PE exotic, reduces pest, CBC
Coccophagus sp. (Hymenoptera: Aphelinidae)	VE endemic, role not quantified, NC
Comperiella bifasciata Howard (Hymenoptera: Encyrtidae)	AR, MX, exotic, established, reduces pest, CBC
Compsilura concinnata (Meigen) (Coleoptera: Coccinellidae)	BB, exotic, not established
Conura (Hymenoptera: Chalcididae)	CR, DR, endemic, reduces pest, ConsBC
Conura anullifera (Walker) (Hymenoptera: Chalcididae)	EC, endemic, reduces pest, NC
Conura destinata (Walker) (Hymenoptera: Chalcididae)	PY, endemic, role not quantified, NC
*Conura fulvovariegata (*Cameron) (Hymenoptera: Chalcididae)	PY endemic, role not quantified, NC
Conura hirtifemora Ashmead (Hymenoptera: Chalcididae)	DR, FA endemic, role not quantified, NC
Conura immaculata (Cresson) (Hymenoptera: Chalcididade)	PY endemic, role not quantified, NC
Conura petioliventris Cameron (Hymenoptera: Chalcididae)	DR, endemic, role not quantified, NC
Conura pseudofulvovariegata (Becker) (Hymenoptera: Chalcididae)	DR, endemic, role not quantified, NC
Conura pulchripes (Cameron) (Hymenoptera: Chalcididade)	PY, endemic, role not quantified, NC
Conura sp. (Hymenoptera: Chalcididae)	CR, PA, PY endemic, reduces pest, NC
Copidosoma desantisi Annecke & Mynhardt (Hymenoptera: Encyrtidae)	MX, exotic, etablished, reduces pest, CBC
Copidosoma floridanum (Ashmead) (Hymenoptera: Encyrtidae)	FA, MX, PY exotic, etablished, reduces pest, CBC
Copidosoma gelechiae Howard (Hymenoptera: Encyrtidae)	BO, endemic, reduces pest, NC
Copidosoma koehleri Blanchard (Hymenoptera: Encyrtidae)	EC endemic, reduces pest, NC
Copidosoma sp. (Hymenoptera: Encyrtidae)	BO, DR, PY, endemic, no data to reduces pest, NC, ABC
Copidosoma truncatellum (Dalman) (Hymenoptera: Encyrtidae)	PA, TT, VE, endemic, role not quantified, NC
Coptera haywardi Loia. (Hym.: Diapriidae)	MX, status unknown
Cotesia (Hymenoptera: Braconidae)	CR exotic, established, reduces pest, ABC

Cotesia flavipes Cameron (Hymenoptera: Braconidae)	AR, BB, BO, BR, CR, CO, EC, FA, GT, JM, MX, PA, PE, PY, RC, TT, UY, VE, exotic, established, reduces pest, ABC
Cotesia glomeratus L. (Hymenoptera: Braconidae)	BB, exotic, not established, no control
Cotesia marginiventris (Cresson) (Hymenoptera: Braconidae)	BO, endemic, role not quantified, NC
Cotesia (Apanteles) sp. poss. *marginiventris* (Cress.), (Hymenoptera: Braconidae)	BB endemic, role not quantified, NC
Cotesia (Apanteles) plutellae (Kurdjumov) (Hymenoptera: Braconidae)	BB, FA, GT, HN, JM, MX, NI, PA, TT, BZ, DM, RC, exotic, established, reduces pest, ABC
Cotesia sp. (Hymenoptera: Braconidae)	1, BB, CR, FA, JM, PY, endemic and exotic, role not quantified, NC
Cotesia vestalis (*plutellae*) (Haliday) (Hymenoptera: Braconidae)	MX, exotic, established, reduces pest, ABC
Cryptochaetum iceryae (Willston) (Diptera: Cryptochaetidae)	EC, exotic, not established
Dacnusa sibirica Telenga (Hymenoptera: Braconidae)	CO, MX, exotic,established, reduces pest, ABC
Dacnusa sp. (Hymenoptera: Braconidae)	FA, endemic, role not quantified, NC
Deleboeae sp. (Hymenoptera: Ichneumonidae)	BO, endemic, reduces pest, NC
Derostenus sp. (Hymenoptera: Eulophidae)	SR, endemic, reduces pest, NC
Descampsina sesamiae Mesnil (Diptera: Tachinidae)	BO, exotic, no further info, not known if established
Diachasmimorpha longicaudata (Ashmead) (Hymenoptera: Braconidae)	AR, BR, CR, EC, FA, MX, PE, SR, exotic, established, reduces pest, ABC
Diachasmimorpha tryoni (Cameron) (Hymenoptera: Braconidae)	AR, CR, MX, exotic, established, reduces pest, ABC, CBC
Diadegma eucerophaga Horstmann (Hymenoptera: Ichneumonidae)	RC, imported but failed to reproduce during rearing
Diadegma insulare (Cresson) (Hymenoptera: Ichneumonidae)	CR, DR, HN, JM, MX, NI endemic, reduces pest, NC,ABC
Diadegma molesta Tschek (Hymenoptera: Ichneumonidae)	AR, exotic, established, role not quantified, CBC
Diadegma pierisae (Rao) (Hymenoptera: Ichneumonidae)	BB, exotic, not established
Diadegma semiclausum Hellen (Hymenoptera: Ichneumonidae)	NI, PA, exotic, established, reduces pest, ABC
Diadegma sp. (Hymenoptera: Ichneumonidae)	BO, PY, endemic, role not quantified, NC
Diadegma varuna Gupta (Hymenoptera: Ichneumonidae)	RC imported but failed to reproduce during rearing
Diadromus collaris (Grav.) (Hymenoptera:Ichneumonidae)	BB, BZ, DM, HN, RC, exotic, not established
Diaeretiella rapae McIntosh (Hymenoptera: Braconidae)	CU, FA, VE, endemic, reduces pest, ABC
Diachasmimorpha longicaudata (Ashmead) (Hymenoptera: Braconidae)	EC, FA, MX, PE, SR, exotic, established, reduces pest, CBC, ABC
Diachasmimorpha tryoni (Cameron) (Hymenoptera: Braconidae)	AR, exotic, established, role not quantified, CBC
Diaulinopsis callichroma Crawford (Hymenoptera: Eulophidae)	FA, endemic, role not quantified, NC
Diglyphus begini (Ashmead) (Hymenoptera: Eulophidae)	CO, DR, FA, endemic, reduces pest, ABC
Diglyphus isaea (Walker) (Hymenoptera: Eulophidae)	BB, CO, DR, MX, PA, exotic, reduces pest, ABC
Diglyphus minoeus (Wlk.) (Hymenoptera: Eulophidae)	BB, exotic, not established

Diglyphus sp. (Hymenoptera: Eulophidae)	BB, DR, PA, exotic, not established
Diglyphus websteri Crawford (Hymenoptera: Eulophidae)	PA, endemic, role not quantified, NC
Dinarmus basalis (Rondani) (Hymenoptera: Pteromalidae)	BB, exotic, not established, CO, endemic, reduces pest, NC, ABC
*Dinarmus vagabundus (*Timb.) (Hymenoptera: Pteromalidae)	BB, exotic, not established
Diplazon laetatorius (Fabricius) (Hymenoptera: Ichneumonidae)	PY, endemic, role not quantified, NC
Dirhinus giffardii (Silvestri) (Hymenoptera: Chalcididae)	BO, CR, MX, exotic, established, reduces pest, CBC, ABC
Dissomphalus spp. (Hymenoptera: Bethylidae)	PY endemic, role not quantified, NC
Disorygma pacifica (Yoshimoto) (Hymenoptera: Eucoliidae)	DR, endemic, role not quantified, NC
Dolichogenidea sp. (Hymenoptera: Braconidae)	PY endemic, role not quantified, NC
Dolichostoma sp. (Diptera: Tachinidae)	BO endemic, role not quantified, NC
Doryctes parvus Mues. (Hymenoptera:Braconidae)	SR endemic, role not quantified, NC
Doryctobracon areolatus Szepligeti (Hymenoptera: Braconidae)	DR, exotic, established, reduces pest, ABC, MX, no info, SR endemic, reduces pest, NC
Doryctobracon cereus (Gahan) (Hymenoptera: Braconidae)	BZ, DM, RC, exotic, established, insufficient control, CBC
Doryctobracon crawfordi (Vier.) (Hymenoptera: Braconidae)	DM, MX, exotic, established, insufficient control, CBC
Doryctobracon sp. (Hymenoptera: Braconidae)	CR, endemic, role not quantified, NC
Doryctobracon trinidadensis (Gah.)(Hymenoptera: Braconidae)	DM, exotic, established, insufficient control, CBC
Drino inhertis (Wied.) (Diptera: Tachinidae)	BO, exotic, no further info, ABC, CBC
Drino sp. (Diptera: Tachinidae)	PA, endemic, role not quantified, NC
Dusona sp. (Hymenoptera:Ichneumonidae)	SR, endemic, reduces pest, NC
Ecphoropsis perdistinctus Viereck (Hymenoptera: Ichneumonidae)	BO exotic, no further info, ABC, CBC
Eiphosoma dentator (Fabricius) (Hymenoptera: Ichneumonidae)	BB, PR, TT, exotic, not established
Eiphosoma sp. (Hymenoptera: Ichneumonidae)	PY endemic, role not quantified, NC
Eiphosoma spp. (Hymenoptera: Ichneumonidae)	VE endemic, role not quantified, NC
Elachertus sp. (Hymenoptera: Eulophidae)	SR endemic, reduces pest, NC
Elasmus sp. (Hymenoptera: Eulophidae)	DR, FA, endemic, reduces pest, NC
Encarsia (= Prospaltella) divergens Silvestri (Hymenoptera: Aphelinidae)	CU, JM, exotic, established, reduces pest, ABC
Encarsia basicincta (Gahan) (Hymenoptera: Aphelinidae)	FA, endemic, role not quantified, NC
Encarsia berlesei Howard (Hymenoptera Aphelinidae)	AR, BR, CU, UY, exotic, established, no control -reduces pest, CBC
Encarsia bimaculata (Heraty and Polaszek) (Hymenoptera: Aphelinidae)	PA, endemic, role not quantifified, NC
Encarsia citrella (Howard) (Hymenoptera: Aphelinidae)	PA endemic, role not quantifified, NC

Encarsia cubensis Gahan (Hymenoptera: Aphelinidae)	FA endemic, role not quantifified, NC
Encarsia dispersa Polazeck (Hymenoptera: Aphelinidae)	FA endemic, role not quantifified, NC
Encarsia formosa Gahan (Hymenoptera: Aphelinidae)	AR, CO, CR, DR, EC, FA, MX, PE, UY, endemic, exotic, reduces pest, NC, ABC
Encarsia guadeloupae Viggiani (Hymenoptera: Aphelinidae)	CR, FA endemic, role not quantifified, NC
Encarsia hispida De Santis (Hymenoptera: Aphelinidae)	FA, PA endemic, role not quantifified, NC
Encarsia longitarsis Myartseva (Hymenoptera: Aphelinidae)	MX, endemic, reduces pest, NC
Encarsia lounsburyi (Berlèse & Paoli) (Hymenoptera: Aphelinidae)	FA endemic, role not quantifified, NC
Encarsia luteola (Howard) (Hymenoptera: Aphelinidae)	FA endemic, role not quantifified, NC
Encarsia meritoria Gahan (Hymenoptera: Aphelinidae)	FA, endemic, role not quantifified, NC, RC exotic, no further info
Encarsia nigricephala Dozier (Hymenoptera: Aphelinidae)	FA, HN, PA, endemic, reduces pest, NC
Encarsia opulenta Silvestri (Hymenoptera: Aphelinidae)	BB, CR, DM, DR, FA, GT, JM, NI, PR, RC, SV, exotic, established, good control, CBC
Encarsia pergandiella Howard (Hymenoptera: Aphelinidae)	FA, HN, PA endemic, reduces pest, NC
Encarsia perniciosi Tower (Hymenoptera: Aphelinidae)	AR, exotic, established, reduces pest, CBC
Encarsia perplexa Huang & Polaszek (Hymenoptera: Aphelinidae)	DR, RC, TT, exotic, established, good control, CBC
Encarsia porteri (Mercet (Hymenoptera: Aphelinidae)	PA, endemic, role not quantified, NC
Encarsia protransvena Viggiani (Hymenoptera: Aphelinidae)	DR endemic, reduces pest, NC
Encarsia quaintancei Howard (Hymenoptera: Aphelinidae)	PA endemic, role not quantified, NC
Encarsia smithi (Silvestri) (Hymenoptera: Aphelinidae)	CU endemic, no control NC
Encarsia sophia (Girault & Dodd) (=*E. transvena*) (Hymenoptera: Aphelinidae)	DR, FA endemic, role not quantified, NC
Encarsia sp. (Hymenoptera: Aphelinidae)	EC, exotic, established, good control, CBC, PA, RC, SR endemic, role not quantified, NC
Encarsia sp. *(parvella* group) (Hymenoptera: Aphelinidae)	DR endemic, role not quantified, NC
Encarsia sp. nr. *pergandiella* Howard (Hymenoptera: Aphelinidae)	DR, HN endemic, reduces pest, NC
Encarsia sp. nr. *variegata* How (Hymenoptera: Aphelinidae)	RC exotic, no further info
Encarsia spp. (Hymenoptera: Aphelinidae)	DR endemic, role not quantified, NC
Encarsia tabacivora (= pergandiella) Viggiani (Hymenoptera: Aphelinidae)	FA endemic, role not quantified, NC
Encarsia telemachusi Evans (Hymenoptera: Aphelinidae)	DR endemic, reduces pest, NC
Encarsiella aleurodici (Girault) (Hymenoptera: Aphelinidae)	CR endemic, reduces pest, NC
Encarsiella new sp. (Hymenoptera: Aphelinidae)	CR endemic, reduces pest, NC

Encarsiella noyesi (Hayat) (Hymenoptera: Aphelinidae)	BB, exotic, established, controls pest, CBC, CR, FA, RC exotic, TT, endemic, reduces pest, NC
Encarsiella sp. D (Hymenoptera: Aphelinidae)	RC, exotic, established, role not quantified, CBC
Encyrtus infelix (Embleton) (Hymenoptera: Encyrtidae)	CR endemic, role not quantified, NC
Encyrtus lecaniorum (Mayr) (Hymenoptera: Encyrtidae)	PE exotic, established, role not quantified, CBC
Enicospilus americanus (Christ) (Hymenoptera: Ichneumonidae)	SV endemic, insufficient control, NC
Ephedrus plagiator (Nees) (Hymenoptera: Braconidae)	AR, exotic, not established. BR exotic, established, reduces pest, CBC
Epidinocarsis lopezi (De Santis) (Hymenoptera: Encyrtidae)	AR, PY endemic, reduces pest, NC
Epiplagiops littoralis Blanchard (Diptera: Tachinidae)	AR endemic, role not quantified, NC
Eretmocerus califonicus Howard (Hymenoptera: Aphelinidae)	MX, exotic, no further info
Eretmocerus eremicus Rose & Zolnerowich (Hymenoptera: Aphelinidae)	CO, MX, exotic, established, reduces pest, ABC
Eretmocerus mundus (Mercet) (Hymenoptera: Aphelinidae)	AR, MX, exotic, established, reduces pest, ABC, CBC
Eretmocerus portoricensis (Dozier) (Hymenoptera:	FA endemic, role not quantified, NC
Eretmocerus serius (Silvestri) (Hymenoptera: Aphelinidae)	BB, CR, CU, HT, JM, PA, RC exotic, established, good control CBC
Eretmocerus sp. (Hymenoptera: Aphelinidae)	HN, exotic, established, insufficient control, ABC, CBC, DO, PA, PY endemic, role not quantified, NC
Eretmocerus tejanus Rose & Zolnerow (Hymenoptera: Aphelinidae)	FA endemic, role not quantified, NC
Eriborus sp. (Hymenoptera: Ichneumonidae)	BO, PE endemic, reduces pest, NC, ABC, CBC
Eucarcelia illota (Curran) (Diptera: Tachinidae)	BO exotic, no further info
Eucelatoria armigera (Coquillet) (Diptera: Tachinidae)	VE endemic, role not quantified, NC
Eucelatoria bryani Sabrosky (Diptera: Tachinidae)	DM exotic, not established, CBC
Euderus sp. (Hymenoptera: Eulophidae)	DR, PY endemic, role not quantified, NC
Eupelmela sp. (Hymenoptera: Eupelmidae)	SR endemic, reduces pest, NC
Eupelmus cushumani (Crawford) (Hymenoptera: Eupelmidae)	PY endemic, role not quantified, NC
Euphorocera floridensis Townsend (Diptera: Tachinidae)	VE endemic, role not quantified, NC
Euplectrus comstockii Howard (Hymenoptera: Eulophidae)	PY, SV endemic, role not quantified, NC
Euplectrus platyhypenae Howard (Hymenoptera: Eulophidae)	BB, CU, JM, endemic, no control - reduces pest, NC, BO , DM VE exotic, not established
Euplectrus sp. (Hymenoptera: Eulophidae)	BB, DR, FA, JM endemic, role not quantified, NC, PR endemic, reduces pest NC
Eupteromalis sp. (Hymenoptera:Pteromalidae)	SR endemic, role not quantified, NC
Eurytoma attiva Burks (Hymenoptera: Eurytomidae)	TT, exotic, no further info

Eurytoma sivinskii Gates & Grissell (Hymenoptera: Eurytomidae)	MX endemic, no further info
Euttetrastichus fennahi (= *Tetrastichus fennahi*) Schauff) (Hymenoptera: Eulophidae)	JM, endemic, role not quantified, NC
Evania laevigata Olivier (= *appendigaster* E. Guerin-Méneville) (Hymenoptera: Evaniidae)	PR endemic, role not quantified, NC
Exasticolus fuscicornis (Cameron) (Hymenoptera: Braconidae)	PY endemic, role not quantified, NC
Fidiobia citri (Nixon) (Hymenoptera:Platygastridae)	BB exotic, no further info, JM, endemic, reduces pest, NC
Fidiobia sp. (Hymenoptera: Platygastridae)	CO, DR endemic, reduces pest, NC, CBC
Fopius arisanus (Sonan) (Hymenoptera: Braconidae)	BR, CR, FA, MX, SR, exotic, established, reduces pest, CBC
Fopius ceratitivorus Wharton (Hymenoptera: Braconidae)	GT exotic, established, reduces pest, CBC
Fopius vandenboschi (Fullaway) (Hymenoptera: Braconidae)	CR, MX exotic, established, reduces pest, CBC
Fornicia sp. (Hymenoptera: Braconidae)	SR endemic, reduces pest, NC
Gahaniella saissetia Timberlake (Hymenoptera: Encyrtidae)	VE, endemic, role not quantified, NC
Galeopsomyia fausta La Salle & Peña (Hymenoptera: Eulophidae)	FA endemic, role not quantified, NC
Ganaspidium utilis Beardsley (Hymenoptera:Figitidae)	DR, HN endemic, reduces pest, NC
Ganaspis sp. (Hymenoptera: Figitidae)	DM, MX, exotic, no further info
Genea (=*Jaynesleskia*) *jaynesi* (Aldrich) (Diptera: Tachinidae)	AR, CO, RC exotic, not established, no success with ABC
Glypta rufiscutellaris Cresson (Hymenoptera: Ichneumonidae)	AR, exotic, established, reduces pest, CBC
Glyptapanteles muesebecki (Blanchard) (Hymenoptera: Braconidae)	PY endemic, role not quantified, NC
Glyptapanteles sp. (Hymenoptera: Braconidae)	DR endemic, reduces pest, NC
Glyptapanteles spp. (Hymenoptera: Braconidae)	BB endemic, reduces pest, NC PY
Goetheana parvipennis (Gahan) (Hymenoptera: Eulophidae)	FA, RC exotic, no further info, JM exotic, established, reduces pest, CBC, ABC
Gonatocerus sp. (Hymenoptera: Mymaridae)	PY endemic, role not quantified, NC
Gonia peruviana Townsend (Diptera:Tachinidae)	BO endemic, reduces pest, NC
Goniophthalmus halli Mesnil (Diptera: Tachinidae)	BO exotic, no further info
Goniozius sp. *punctulaticeps* group (Hymenoptera: Bethylidae)	BB exotic, not established
Goniozus legneri (Gordh) (Hymenoptera: Bethylidae)	AR, CL, endemic, reduces pest, NC, ABC
Goniozus natalensis Gordh (Hymenoptera: Bethylidae)	BO, exotic, no further ino
Gyranusoidea indica Shafee, Alam & Argarwal (Hymenoptera: Encyrtidae)	BZ, DR, JM, MX, PR, RC exotic, established, reduces pest, CBC
Gyranusoidea sp. (Hymenoptera: Encyrtidae)	FA exotic, under testing for CBC
Gyranusoidea tebygi Noyes (Hymenoptera: Encyrtidae)	FA exotic, under testing for CBC
Gyron triatomae Msn (Hymenoptera: Scelionidae)	BO, exotic, not established

Habrobracon (Hymenoptera: Braconidae)	DR, exotic, no further info
Habrobracon hebetor Say (Hymenoptera: Braconidae)	BR, MX, exotic, mass reared, reduces pest, ABC
Habrobracon spp. (Hymenoptera: Braconidae)	MX exotic, mass reared, reduces pest, ABC
Halticoptera circulus (Walker) (Hymenoptera: Peromalidae)	DR, FA, endemic, role not quantified, NC
Halticoptera sp. (Hymenoptera: Pteromalidae)	PA endemic, role not quantified, NC
Hambletonia pseudococcinna Comp. (Hymenoptera: Eulophidae)	JM, endemic, role not quantified, NC
Heterospilus annulicornis Muesebeck (Hymenoptera: Braconidae)	PY endemic, role not quantified, NC
Heterospilus coffeicola Schmiedeknecht (Hymenoptera: Braconidae)	CO, CR, exotic, established, reduces pest, CBC, ABC
Heterospilus gossypii Muesebeck (Hymenoptera: Braconidae)	PY endemic, role not quantified, NC
Heterospilus hambletoni Muesebeck (Hymenoptera: Braconidae)	PY endemic, role not quantified, NC
Heterospilus sp. (Hymenoptera: Braconidae)	SR, endemic, role not quantified, NC
Homalotylus eytelweinii (Ratzeburg) (Hymenoptera: Encyrtidae)	PY endemic, role not quantified, NC
Hoplognathoca sp. (Hymenoptera: Mutillidae)	PY endemic, role not quantified, NC
Horismenus (Hymenoptera: Eulophidae)	CR endemic, role not quantified, NC
Horismenus crassus Hansson (Hymenoptera: Eulophidae)	PY endemic, role not quantified, NC
Horismenus sp. (Hymenoptera: Eulophidae)	CR, DR, JM, PY endemic, reduces pest, NC, ConsBC,
Horismenus sp.nr. *cupreus* (Ashm.) (Hymenoptera: Eulophidae)	JM endemic, role not quantified, NC
Horismenus spp. (Hymenoptera: Eulophidae)	FA endemic, role not quantified, NC
Hyalomyia chilensis Macq (Diptera: Tachinidae)	RC exotic, not established
Hypomicrogaster hypsipylae De Santis (Hymenoptera:Braconidae)	CR endemic, reduces pest, NC
Ibalia leucospoides (Hochenwarth) (Hymenoptera: Ibaliidae)	AR, BR, CL, UY, exotic, established, reduces pest, CBC, ABC
Incamya chilensis Aldrich (Diptera: Tachinidae)	EC endemic, reduces pest, NC, ABC
Iphiaulax kimballi Kirkland (Hymenoptera: Braconidae)	BO, exotic, no further info
Iphiaulax grenadensis (Ashm.) (Hymenoptera:Braconidae)	SR, exotic, no further info
Ipobracon grenadensis Ashmead (Hymenoptera:Braconidae)	BB, RC,exotic, not established, TT endemic, reduces pest, NC
Ipobracon puberuloides Myers (Hymenoptera: Braconidae)	BB exotic, not established
Isosomodes sp. (Hymenoptera: Eurytomidae)	VE endemic, role not quantified, NC
Itoplectus narangae Ashm. (Hymenoptera: Ichneumonidae)	BO exotic, no further info
Larra americana Saussure (Hymenoptera: Crabronidae)	VE endemic, role not quantified, NC
Larra bicolor Fabricius (Hymenoptera: Crabronidae)	BO endemic, reduces pest, NC
Larra sp. (Hymenoptera: Crabonidae)	BO endemic, reduces pest, NC

Larra transandina Williams (Hymenoptera: Crabronidae)	BO endemic, reduces pest, NC
Lecanobius (Hymenoptera: Eupelmidae)	CL exotic, no futher info
Lecanobius utilis Compere (Hymenoptera: Eupelmidae)	PE exotic, established, reduces pest, CBC
Leptocybe invasa Fisher & La Salle (Hymenoptera: Eulophidae)	BR exotic, established, reduces pest, CBC
Leptomastidae sp. (Hymenoptera: Encyrtidae)	PR endemic, role not quantified, NC
Leptomastidea abnormis (Girault) (Hymenoptera: Encyrtidae)	CL, CR exotic, no further info, PE endemic, reduces pest, NC
Leptomastix dactylopii (Howard) (Hymenoptera: Encyrtidae)	CL, MX exotic, no further info
Leptomastix epona (Noyes) (Hymenoptera: Encyrtidae)	CL exotic, established, reduces pest, CBC
Leptopilina boulardi Förster (Hymenoptera: Figitidae)	MX endemic, role not quantified, NC
Leskiopalpus diadema Wied. (Diptera: Tachinidae)	SR endemic, reduces pest, NC
Lespesia archippivora (Riley) (Diptera: Tachinidae)	HN endemic, reduces pest, NC
Lespesia n.sp. (Diptera: Tachinidae)	SV endemic, role not quantified, NC
Lespesia sp. (Diptera: Tachinidae)	PA endemic, role not quantified, NC
Lipolexis oregmae (Gahan) (Hymenoptera: Aphidiidae)	JM endemic, reduces pest, NC, ABC
Litomastix sp. (Hymenoptera: Encyrtidae)	BB endemic, reduces pest, NC
Litomastix sp. nr. *truncatella* (Dalm.) (Hymenoptera: Encyrtidae)	BB exotic, established, reduces pest, CBC
Lixadmontia franki Wood and Cave (Diptera: Tachinidae)	HN endemic, reduces pest, NC
Lixophaga diatraeae (Townsend) (Diptera: Tachinidae)	AR, BB, BO, CO, CR, CU, DM, DR, EC, FA, HT, JM, MX, PA, PR, RC, TT, exotic, established, from poor to complete control, CBC, ABC + endemic, reduces pest, NC, ABC
Lydella minense (Town.) (Diptera: Tachinidae)	AR, BO, BR, CO, CR, CU, DM, DR, EC, FA, GY, JM, RC, SR, VE exotic, established, from poor to complete control, CBC, ABC + endemic, reduces pest, NC, ABC
Lydinolydella metalica Townsend (Diptera: Tachinidae)	AR exotic, not established
Lysiphlebus testaceipes (Cresson) (Hymenoptera: Braconidae)	AR, BR, CR, CU, FA, JM, MX, PY, TT, VE exotic, established, reduces pest, ABC, CBC + endemic, reduces pest, NC, ABC
Macrocenctrus prolificus (Hymenoptera: Braconidae)	BO, MX exotic, not clear if established, no further info, CBC
*Macrocentrus ancylivorus (*Rohwer) (Hymenoptera: Braconidae)	AR, BR,UY exotic, not established – established, no control – reduces pest, CBC
Macrocentrus delicatus (Cresson) (Hymenoptera: Braconidae)	AR, exotic, established, role not quantified, CBC
Macrocentrus sp. (Hymenoptera: Braconidae)	BB exotic, not established
Macromalon orientale Kerrich (Hymenoptera: Ichneumonidae)	BB exotic, not established
Mastrus ridens Horstmann (Hymenoptera: Ichneumonidae)	AR endemic, reduces pest, NC

Mastrus ridibundus (Gravenhorst) (Hymenoptera: Ichneumonidae)	AR exotic, established, reduces pest, CBC
Megaphragma sp. (Hymenoptera:Trichogrammatidae)	FA endemic, role not quantified, NC
Megarhyssa nortoni (Cresson) (Hymenoptera: Ichneumonidae)	AR, BR, CL exotic, established, reduces pest, CBC
Megaselia sp. (Diptera: Phoridae)	SR endemic, reduces pest, NC
Metaphycus (Hymenoptera: Encyrtidae)	RC endemic, role not quantified, NC
Metaphycus cereales sp. nov. Myartseva & Ruiz (Hymenoptera:Encyrtidae)	MX endemic, role not quantified, NC
Metaphycus flavus (Howard) (Hymenoptera: Encyrtidae)	CL exotic, established, reduces pest, CBC
Metaphycus helvolus (Compere) (Hymenoptera: Encyrtidae)	AR, CL, CR, PE, exotic, established, reduces pest, CBC
Metaphycus lounsburyi (Howard) (Hymenoptera: Encyrtidae)	AR, PE exotic, established, reduces pest, CBC
Metaphycus stanleyi Compere (Hymenoptera: Encyrtidae)	CL exotic, established, reduces pest, CBC
Meteorus laphygmae Vier. (Hymenoptera:Braconidae)	PY, SR, VE endemic, role not quantified, NC
Meteorus sp. (Hymenoptera:Braconidae)	BO endemic, reduces pest, NC
Microcharops sp. (Hymenoptera: Ichneumonidae)	BO, PY endemic, reduces pest, NC
Microplitis plutellae Muesbeck (Hymenoptera: Braconidae)	NI, PA exotic, established, reduces pest, ABC
Microplitis sp. (Hymenoptera: Braconidae)	BO exotic, established, reduces pest, ABC
Microseromasia sphenophori Vill (Diptera: Tachinidae)	BO, exotic, no further info
Miobiopsis diadema (Wiedemann) (Diptera:Tachinidae)	TT, endemic, reduces pest, NC
Mirax insularis Mues. (Hymenoptera:Braconidae)	DM, FA, endemic, reduces pest, NC, PR exotic, established, reduces pest, CBC
Muscidifurax raptor (Girault & Sanders) (Hymenoptera: Pteromalidae)	EC exotic, no further info MX endemic, reduces pest, NC, VE exotic, no further info
Muscidifurax raptorellus Kogan & Legner (Hymenoptera: Pteromalidae)	BR, endemic, reduces pest, NC, ABA, MX, PE exotic, established, reduces pest ABC
Muscidifurax spp. (Hymenoptera: Pteromalidae)	BB, exotic, role not quantified, ABC, MX exotic, established, reduces pest, ABC
Muscidifurax uniraptor Kogan & Legner (Hymenoptera: Pteromalidae)	BR, endemic, reduces pest, NC, ABC, RC exotic, not established
Muscidifurax zaraptor Kogan & Legner (Hymenoptera: Pteromalidae)	MX, PE, exotic, no further info
Myiopharus doryphorae (Riley) (Diptera: Tachinidae)	JM endemic, role not quantified, NC
Myzinum ephippium F. (= *M. xanthonotus* (Rohw.) (Hymenoptera: Tiphiidae)	BB exotic, not established
Myzinum haemorrhoidalis F. (Hymenoptera: Tiphiidae)	BB exotic, not established
Nasonia spp. (Hymenoptera: Pteromalidae)	MX exotic, no further info
Nasonia vitripennis (Walker) (Hymenoptera: Pteromalidae)	BR, endemic, role not quantified, NC, MX exotic, no further info

Nemorilla sp. (Diptera: Tachinidae)	BB, PY endemic, insufficient control, NC
Neocatolaccus longiventris (Gahan) (Hymenoptera: Pteromalidae)	PY endemic, role not quantified, NC
Neochrysocharis sp. (Hymenoptera: Eulophidae)	DR endemic, role not quantified, NC
Neodusmetia sangwani (Subba Rao) (Hymenoptera: Encyrtidae)	BR, MX exotic, established, CBC
Odontosema anastrephae Borgmeier (Hymenoptera: Figitidae)	MX exotic, no further info
Oenonogastra microrhopalae Ashmead (Hymenoptera: Braconidae)	PA endemic, role not quantified, NC
Oenonogastra sp. (Hymenoptera: Braconidae)	PA endemic, role not quantified, NC
Oligosita giraulti Crawford (Hymenoptera: Trichogrammatidae)	TT endemic, role not quantified, NC
Ooencyrtus (Hymenoptera: Encyrtidae)	CR endemic, reduces pest, NC
Ooencyrtus sp. (Hymenoptera: Encyrtidae)	CO, RC, endemic, reduces pest, ABC, SR endemic, role not quantified
Ooencyrtus submetallicus Howard (Hymenoptera: Encyrtidae)	BO, DR, RC, TT, endemic, role not quantified, NC
Ooencyrtus trinidadensis Crawford (Hymenoptera: Encyrtidae)	RC, VE endemic, role not quantified, NC
Oomyzus sokolowskii (Kurdj.) (Hymenoptera: Eulophidae)	FA, JM endemic, reduces pest, NC
Oomyzus sp. (Hymenoptera: Eulophidae)	PY endemic, role not quantified, NC
Ooyncyrtus sp. (Hymenoptera: Encyrtidae)	JM endemic, role not quantified, NC
Ophion spp.(Hymenoptera: Ichneumonidae)	PY, UR, endemic, role not quantified, NC
Opius crawfordi Viereck (Hmenoptera: Braconidae)	AR exotic, not established
Opius oophilus Fullaway (Hymenoptera: Braconidae)	BO, AR, CR, exotic, not established, but reduced pest in ABC
Opius anastrephae Vier (Hymenoptera: Braconidae)	DM endemic, insufficient control
Opius bellus Gahan (Hymenoptera: Braconidae)	DM, exotic, established, insuff control CBC, SR endemic, role not quantified, NC
Opius cereus (Gah) (Hymenoptera: Braconidae)	SR endemic, insufficient control, NC
Opius concolor Szépligeti (Hymenoptera: Braconidae)	EC exotic, no further info, CBC
Opius concolor var. *Siculus Mon.* (Hymenoptera: Braconidae)	BO exotic, no further info, CBC
Opius dimidiatus Ashmead (Hymenoptera: Braconidae)	PA endemic, role not quantified, NC
Opius dissitus Muesebeck (Hymenoptera:Braconidae)	HN endemic, role not quantified, NC
Opius forticornis Cameron (Hymenoptera: Braconidae)	DR endemic, role not quantified, NC
Opius hirsutus Tobias (Hymenoptera: Braconidae)	MX exotic, no further info
Opius zoophilous Fullaway (= *Biosteres arisanus* (Sonan) (Hymenoptera: Braconidae)	BO, CR exotic, established, reduces pest, ABC
Opius sp. (Hymenoptera: Braconidae)	BB, BZ, FA, SR, exotic, no further ingo
Opius spp. (Hymenoptera: Braconidae)	DR, endemic, role not quantified, NC, RC exotic, not established

Orgilus lepidus (Muesebeck) (Hymenoptera: Braconidae)	PE, exotic, no further info
Orgilus obscurator (Nees) (Hymenoptera: Braconidae)	CL, exotic, established, good control, CBC
Ormia depleta (Wiedemann) (Diptera: Tachinidae)	BO endemic, reduces pest, NC
Ormyrus orientalis Walker (Hymenoptera: Ormyridae)	DR exotic, died during transport
Pachycrepoideus sp. (Hymenoptera: Pteromalidae)	CO, exotic, established, reduces pest, ABC
Pachycrepoideus vindemiae Rondani (Hymenoptera: Pteromalidae)	AR, BB, BZ, BO, BR, CO, CR, DM, JM, MX, PE, RC, TT, exotic, established, no control - reduces pest, ABC, CBC
Pachyneuron albutium Walker (Hymenoptera: Pteromalidae)	PY endemic, role not quantified, NC
Pachyneuron spp. (Hymenoptera: Pteromalidae)	DR, PY endemic, role not quantified, NC
Pachyneuron aphidis (Bouché) (Hymenoptera: Pteromalidae)	FA endemic, role not quantified, NC
Palpozenillia diatraea Townsend (Diptera: Tachinidae)	BO, endemic, reduces pest, NC, ABC, RC exotic, not established
Palpozenillia palpalis Aldrich (Diptera: Tachinidae)	AR, BO, GY, exotic, no further info,
Pammaecerus leptotrichopus (B.-B.) (Diptera:Tachinidae)	SR endemic, reduces pest, NC
Parahormius pallidipes Ashm. (Hymenoptera: Braconidae)	DR endemic, role not quantified, NC
Parania tricolor (Szépligeti) (Hymenoptera: Ichneumonidae)	PY endemic, role not quantified, NC
Patasson nitens (Girault) (Hymenoptera: Mymaridae)	AR exotic, established, reduces pest, CBC
Pauridia peregrina (Timberlake) (Hymenoptera: Encyrtidae)	CL exotic, no further info
Pediobius cajanus Taveras & Hansson (Hymenoptera: Eulophidae)	DR endemic, reduces pest, NC
Pediobius furvus (Gah.) (Hymenoptera: Eulophidae)	BO, exotic, no further info RC exotic, not established
Perisierola nigrifemur Ashmead (Hymenoptera: Bethylidae)	BB, endemic, reduces pest, NC, RC endemic, insuff control, NC
Peristenus relictus (= *stygicus*) Loan (Hymenoptera: Braconidae)	MX, exotic, established, reduces pest, CBC
Phanerotoma bennetti Mues (Hymenoptera: Braconidae)	BB, exotic, not established
Phanerotoma sp. (Hymenoptera: Braconidae)	BB, BZ exotic, not established
Phymastichus coffea LaSalle (Hymenoptera: Eulophidae)	CO, CR, CU, EC, HT, JM, MX, PA exotic, established, reduces pest, ABC, CBC
Phytomyptera sp. (Diptera: Tachinidae)	BO, endemic, reduces pest, NC
Pimpla sp. (Hymenoptera: Ichneumonidae)	PY endemic, role not quantified, NC
Plagioprospherysa trinitatis Thomps (Diptera: Tachinidae)	BB endemic, reduces pest, NC
Plagiotrypes sp. (Hymenoptera: Ichneumonidae)	PY endemic, role not quantified, NC
Platystasius citri Nixon (Hymenoptera: Platygastridae)	JM endemic, role not quantified, NC
Praon gallicum Stary (Hymenoptera: Braconidae)	AR, exotic, no further info BR, exotic, established, reduces pest, CBC
Praon volucre (Haliday) (Hymenoptera: Braconidae)	BR exotic, established, reduces pest, CBC

Pristomerus (Hymenoptera: Ichneumonidae)	CR endemic, role not quantified, NC
Prochiloneurus sp. (Hymenoptera: Encyrtidae)	PY endemic, role not quantified, NC
Prorops nasuta Waterston (Hymenoptera: Bethylidae)	BR, JM, exotic, no success CO, CR, EC, HT, MX, PA, exotic, established, reduces pest, ABC, CBC
Prospaltella berlesi How (Hymenoptera: Aphelinidae)	BO, PE, exotic, established, reduces pest, CBC
Prospaltella opulenta Silvestri *(=Encarsia opulenta)* (Hymenoptera: Aphelinidae)	SV,VE exotic, established, reduces pest, CBC
Protolaccus bacchadis Burks (Hymenoptera: Pteromalidae)	SR endemic, role not quantified, NC
Pseudapanteles dignus Muesebeck (Hymenoptera: Braconidae)	AR , DR, endemic, reduces pest, NC
Pseudapanteles sp. (Hymenoptera: Braconidae)	FA endemic, role not quantified, NC
Pseudaphycus angelicus (Howard) (Hymenoptera: Encyrtidae)	PR, RC, exotic, established, reduced pest, CBC
Pseudaphycus flavidulus (Bréthes) (Hymenoptera: Encyrtidae)	CL, exotic, no further info
Pseudaphycus utilis Timberlake (Hymenoptera: Encyrtidae)	RC exotic, not established
Pseudleptomastrix mexicana Noyes e Schauff (Hymenoptera: Encyrtidae)	JM, RC, exotic, established, controlled pest, CBC
Pseudogonatopus saccharivorae Richards (Hymenoptera: Dryinidae)	JM, TT, exotic, not established
Pseudophycus perdignus (Compere) (Hymenoptera: Encyrtidae)	CL exotic, no further info
Psyllaephagus bliteus Riek (Hymenoptera: Encyrtidae)	MX, PY, UY exotic, established, reduces pest, CBC
Psyllaephagus pilosus Noyes (Hymenoptera: Encryrtidae)	BR, CL, PE, UY exotic, established, reduces pest, CBC
Psyllaephagus yaseeni Noyes (Hymenoptera: Encyrtidae)	TT exotic, no further info
Psyttalia concolor (Szepligati) (Hymenopteraa: Braconidae)	CR, exotic, established, reduces pest, ABC
Psyttalia (Opius) incisi (Silvestri) (Hymenoptera: Braconidae)	BO, CR, exotic, established, reduces pest, ABC
Pteromalus puparum (Linné) (Hymenoptera: Pteromalidae)	BB, FA, RC exotic, not established
Rhaconotus rosiliensis Lal. (Hymenoptera: Braconidae)	BO exotic, no further info
Rhaconotus sp. (Hymenoptera: Braconidae)	PY endemic, role not quantified, NC
Rhyssa persuasoria (L.) (Hymenoptera: Icchneumonidae)	AR, BR exotic, established, reduces pest, CBC
Rogas aligarhensis (Quadri) (Hymenoptera: Braconidae)	BB, exotic, no further info
Rogas gossypii Muesebeck (Hymenoptera: Braconidae)	VE endemic, role not quantified, NC
Rogas spp. (Hymenoptera: Braconidae)	UY endemic, role not quantified, NC
Rogas vaughani Muesebeck (Hymenoptera: Braconidae)	NI endemic, role not quantified, NC
Sarcodexia sternodontis Townsend (Diptera: Sarcophagidae)	VE endemic, role not quantified, NC
Sarcophaga acridiorum Weyenberg (Diptera: Sarcophagidae)	PY endemic, role not quantified, NC
Sarcophaga caridei Brèthes (Diptera: Sarcophagidae)	PY, VE endemic, role not quantified, NC

Sarcophaga sp. (Diptera: Sarcophagidae)	RC endemic, role not quantified, NC
Scelio aegyptiacus Priesner (Hymenoptera: Scelionidae)	BB exotic, not established
Scelio famelicus (Say) (Hymenoptera: Scelionidae)	VE endemic, role not quantified, NC
Scelio sp. nr. *serdangensis* (Timb.) (Hymenoptera: Scelionidae)	BB exotic, not established
Scutellista (Hymenoptera: Pteromalidae)	CL exotic, established, role not quantified, CBC
Scutellista cyanea Motschulsky (Hymenoptera: Pteromalidae)	AR, BO, PE, exotic, established, reduces pest, CBC
Selitrichodes neseri Kelly & La Salle (Hymenoptera: Eulophidae)	BR exotic, established, reduces pest, CBC
Smicra (= Conura) punctata (Fabricius) (Hymenoptera: Chalcididae)	PR endemic, reduces pest, NC
Smicra emarginata (Fabricius) (Hymenoptera: Chalcididae)	PR endemic, reduces pest, NC
Smicra flavopicta Cresson (Hymenoptera: Chalcididae)	PR endemic, reduces pest, NC
Smicra ignea (Cresson) (Hymenoptera: Chalcididae)	PR endemic, reduces pest, NC
Spalangia cameroni (Perkins) (Hymenoptera: Pteromalidae)	AR, BO, BR, CO, MX, exotic, established, reduces pest, ABC
Spalangia endius (Walker) (Hymenoptera: Pteromalidae)	AR, BO, BR, MX, PE, VE exotic, established, reduces pest, ABC
Spalangia gemina Boucek (Hymenoptera: Pteromalidae)	AR, BR exotic, established, reduces pest, ABC
Spalangia nigra Latreille (Hymenoptera: Pteromalidae)	BB exotic, no further info
Spalangia nigroaenea Curtis (Hymenoptera: Pteromalidae)	MX exotic, no further info
Spalangia simplex Perkins (Hymenoptera: Pteromalidae)	MX exotic, no further info
Spalangia sp. (Hymenoptera: Pteromalidae)	CO, MX exotic, established, reduces pest, ABC
Spalangia spp. (Hymenoptera: Peromalidae)	CR, EC, MX exotic, established, reduces pest, ABC
Sphegigaster sp. (Hymenoptera: Pteromalidae)	BB exotic, no further info
Spilocalcis sp. (Hymenoptera: Chalcididae)	JM, SR endemic, reduces pest, NC
Spilochalcis dux (Walker) (Hymenoptera: Chalcididae)	VE endemic, role not quantified, NC
Spilochalcis fulvomaculata (Cameron) (Hymenoptera: Chalcididae)	VE endemic, role not quantified, NC
Spilochalcis hirtifemora (Ashmead.) (Hymenoptera: Chalcididae)	BB, RC endemic, insufficient control, NC
Spilochalcis torvina (Cress.) (Hymenoptera: Chalcididae)	RC endemic, insufficient control, NC
Stantonia sp. (Hymenoptera: Braconidae)	PA endemic, reduces pest, NC
Stenocranophilus quadratus Pierce (Strepsiptera: Halictophagidae)	JM endemic, reduces pest, NC
Strabotes rupelae nov. spec Zwart (Hymenoptera: Ichneumonidae)	SR endemic, role not quantified
Synopeas sp. (Hymenoptera: Platygasteridae)	BB, TT, exotic, not established
Syrphophagus aphidivorus (Mayr) (Hymenoptera: Encyrtidae)	FA, PY endemic, role not quantified, NC
Syrphophagus nigricornis (De Santis) (Hymenoptera: Encyrtidae)	PY endemic, role not quantified, NC

Syrphophagus sp. (Hymenoptera: Encyrtidae)	PY endemic, role not quantified, NC
Tachinaephagus zealandicus Ashmead (Hymenoptera: Encyrtidae)	BR endemic, role not quantified, NC
Tamarixia leucaenae Boucek (Hymenoptera: Eulophidae)	TT exotic, no further info
Tamarixia radiata (Waterston) (Hymenoptera: Eulophidae)	AR, BB, BR, BZ, CO, CR, DR, FA,JM, MX, NI, PY, PR, UY, exotic, established, reduces pest, CBC
Tamarixia triozae (Burks) (Hymenoptera: Eulophidae)	MX exotic, no further info
Telenomus (Hymenoptera: Platigastridae)	CR endemic, reduces pest, NC
Telenomus alecto Crawford (Hymenoptera: Platigastridae)	BB, endemic, insuff control, NC, BO endemic, reduces pest, ABC, RC exotic, not established,RC, VE, endemic, role not quantified, NC
Telenomus alsophilae Viereck (Hymenoptera: Platigastridae)	CO, PE exotic, established, reduces pest, ABC
Telenomus basalis (Wollaston) (Hymenoptera: Platigastridae)	AR exotic, established, no further info
Telenomus connectans Ashm (Hymenoptera: Platigastridae)	SR endemic, good control, NC
Telenomus fariai Costa Lima (Hymenoptera: Platigastridae)	BO endemic, insuff control, NC
Telenomus nawaii Ash. (Hymenoptera: Platigastridae)	BO exotic, no further info
Telenomus nigrocoxalis Ashm (Hymenoptera: Platigastridae)	SR endemic, reduces pest, NC
Telenomus podisi Ashmead (Hymenoptera: Platigastridae)	BO, BR, PA, PY, UY endemic, reduces pest, NC, ABC
Telenomus remus Nixon (Hymenoptera: Platigastridae)	BB, BO, CO, DM, FA, HN, MX, PE, PY, RC, SV, TT, VE exotic, established, reduces pest, ABC, CBC
Telenomus rowani (Gahan) (Hymenoptera: Platigastridae)	PA endemic, reduces pest, NC
Telenomus sp. (Hymenoptera: Platigastridae)	BO, CO, CU, DR, EC, FA, GY, PE, PY, SR, endemic, exotic, established, reduces pest, NC, ABC, CBC
Telenomus sp. near *alecto* (Hymenoptera: Platigastridae)	AR exotic, no further info
Telenomus sp. prob. *dilophonotae* Cam. (Hymenoptera: Platigastridae)	SR endemic, good control, NC
Temelucha spp. (Hymenoptera: Ichneumonidae)	DR endemic, role not quantified, NC
Tetracnemus pretiosus (Timberlake) (Hymenoptera: Encyrtidae)	CL exotic, no further info
Tetramesa romana (Walker) (Hymenoptera: Eurytomidae)	MX exotic, established, reduces pest, ABC
Tetrastichus (= Oomyzus) sokolowski Kurd. (Hymenoptera:Eulophidae)	BB, FA, RC, TT exotic, established, reduces pest, CBC, ABC
Tetrastichus gala Walker (= *Tetrastichus marylandensis)* (Hymenoptera:Eulophidae)	DM, endemic, role not quantified, NC JM, RC exotic, not further inro
Tetrastichus gallerucae (Fonscolmber) (Hymenoptera: Eulophidae)	AR exotic, no further info
Tetrastichus giffardianus Silvestri (Hymenoptera: Eulophidae)	BR, UY exotic, established, insuff control, CBC
Tetrastichus haitiensis Gah. (Hymenoptera: Eulophidae)	BB, exotic, not established DR, endemic, reduces pest ABC,CBC, JM, PR endemic, reduces pest, NC

Tetrastichus howardi (Olliff) (Hymenoptera: Eulophidae)	CU, PE endemic, reduces pest, NC, ABC
Tetrastichus sp. (Hymenoptera: Eulophidae)	BZ, endemic, some control, NC JM SR, SV endemic, reduces pest, NC
Tetrastichus sp. nr. *vaquitarum* Wolc. (Hymenoptera: Eulophidae)	BZ, endemic, some control, NC JM exotic, not established
Tetrastichus spirabilis Waterston (Hymenoptera: Eulophidae)	BZ exotic, not established
Theronia lineata (Fabricius) (Hymenoptera: Ichneumonidae)	PY endemic, role not quantified, NC
Theronia sp. (Hymenoptera: Ichneumonidae)	SR endemic, role not quantified, NC
Thersilochus argentinensis (Blanchard) (Hymenoptera: Ichneumonidae)	AR endemic, role not quantified, NC
Thersilochus parkeri (Blanch.) (Hymenoptera: Ichneumonidae)	AR endemic, role not quantified, NC
Thripastichus gentilei (Del Guercio) (Hymenoptera: Eulophidae)	FA endemic, role not quantified, NC
Tiphia parallela Smith (Hymenoptera: Scoliidae)	BB, RC endemic, reduces pest, ConsBC, NC
Tiphia sp. (Hymenoptera: Scoliidae)	PR exotic, no further ino
Triaspis sp. (Hymenoptera: Braconidae)	AR, PY endemic, role not quantified, NC
Trichogramma (Hymenoptera: Trichogrammatidae)	BB, CL, CR, CU, EC, DR endemic, exotic, not established – established, no control to reduces pest, NC,ABC
Trichogramma (*semifumatum*) *pretiosum* Riley (Hymenoptera: Trichogrammatidae)	BO, exotic, not established
Trichogramma atopovirilia Oatman and Platner (Hymenoptera: Trichogrammatidae)	BR, CO, PE, VE endemic, reduces pest, NC, ABC
Trichogramma australicum (Girult.) (Hymenoptera: Trichogrammatidae)	AR, exotic, not established, CO endemic, reduces pest, ABC
Trichogramma beckeri (Nagarkatti) (Hymenoptera: Trichogrammatidae)	CR endemic, reduces pest, NC
Trichogramma bennetti Nagaraja & Nagarkatti (Hymenoptera: Trichogrammatidae)	VE endemic, role not quantified
Trichogramma brasiliensis (Ashm.) (Hymenoptera: Trichogrammatidae)	PE, exotic, established, reduces pest, ABC,RC endemic, role not quantified, NC
Trichogramma brassicae Bezdenko (Hymenoptera: Trichogrammatidae)	MX, PE, exotic, no further info
Trichogramma bruni Nagaraja (Hymenoptera: Trichogrammatidae)	VE endemic, role not quantified, NC
Trichogramma cacoeciae Marchal (Hymenoptera: Trichogrammatidae)	AR, exotic, not established CL endemic, no further info, NC, PE exotic, no further info
Trichogramma chilonis Ishii (Hymenoptera: Trichogrammatidae)	BB, FA endemic, role not quantified, NC
Trichogramma dendrolimi Matsumura (Hymenoptera: Trichogrammatidae)	PE exotic, no further info
Trichogramma diazi Velásquez and Terán (Hymenoptera: Trichogrammatidae)	VE endemic, role not quantified, NC
Trichogramma euproctidis (Girault) (Hymenoptera: Trichogrammatidae)	AR exotic, established, reduces pest, CBC
Trichogramma evanescens Westwood (Hymenoptera: Trichogrammatidae)	BO, MX exotic, no further info
Trichogramma exiguum Pinto & Platner (Hymenoptera: Trichogrammatidae)	BB, RC, endemic, insuffic control, NC, ABC, CO, CU, MX, PE, UY, VE endemic, reduces pest, ABC,

*Trichogramma fasciatum (*Perkins) (= *minutum* auct.) (Hymenoptera:Trichogrammatidae)	BB endemic, reduces pest, ABC
Trichogramma fasciatum (Perkins) (Hymenoptera: Trichogrammatidae)	BB, CR, endemic, reduces pest, ABC , NC ,BO endemic, no further info
Trichogramma fuentesi Torre (Hymenoptera: Trichogrammatidae)	BB, CU, endemic, reduces pest, ABC, PE, exotic, reduces pest, ABC, VE endemic, no further info
Trichogramma galloi Zucchi (Hymenoptera: Trichogrammatidae)	BO, BR, PE, UY, VE endemic, reduces pest, ABC
Trichogramma lasallei Pinto (Hymenoptera: Trichogrammatidae)	VE endemic, no further info
Trichogramma lopezandinensis Sarmiento (Hymenoptera: Trichogrammatidae)	PE exotic, no further info
Trichogramma marandobai Brun, Moraes e Soares (Hymenoptera: Trichogrammatidae)	PE exotic, no further info
Trichogramma minutum Riley (Hymenoptera: Trichogrammatidae)	BB, CO, EC, MX, NI, PE, PR, RC, VE endemic, exotic, established, reduces pest NC, ABC
Trichogramma near *pretiosum* (Hymenoptera: Trichogrammatidae)	CR endemic, reduces pest, NC
*Trichogramma nerudai (*Pintureau & Gerding) (Hymenoptera: Trichogrammatidae)	AR, CL, endemic, reduces pest, NC PE exotic, reduces pest, CBC
Trichogramma nubilale Ertie & Davis (Hymenoptera: Trichogrammatidae)	FA endemic, role not quantified, NC
Trichogramma obscurum Pinto (Hymenoptera: Trichogrammatidae)	VE endemic, role not quantified, NC
Trichogramma perkinsi Girault (Hymenoptera: Trichogrammatidae)	AR, exotic, not established CO exotic, role not quantified, ABC
Trichogramma pintoi Voegelé (Hymenoptera: Trichogrammatidae)	CU, MX, PE, VE exotic, established, reduces pest, ABC
Trichogramma platneri Nagarkatti (Hymenoptera: Trichogrammatidae)	MX, exotic, no further info
Trichogramma pretiosum Riley (Hymenoptera: Trichogrammatidae)	BO, BR, CO, CR, CU, EC, DR, FA, GT, MX, NI, PA, PE, PY, UY, VE endemic, exotic, reduces pest, ABC, NC
Trichogramma rojasi Nagaraja & Nagarkatti (Hymenoptera: Trichogrammatidae)	CU, endemic, reduces pest, ABC
Trichogramma semifumatum (Perkins) (Hymenoptera: Trichogrammatidae)	CR, EC, SV. endemic, reduces pest, NC, ABC
Trichogramma sp. (Hymenoptera: Trichogrammatidae)	BB, CL, CO, EC , GY, JM, PA, PE, RC, VE, endemic, exotic, established, no control – reduces pest, NC, ABC
Trichogramma sp1 (Hymenoptera: Trichogrammatidae)	VE endemic, role not quantified, NC
Trichogramma sp2 (Hymenoptera: Trichogrammatidae)	VE endemic, role not quantified, NC
Trichogramma spp. (Hymenoptera: Trichogrammatidae)	1, BB, BO, CL,CO, CR, CU, DR, EC, MX, PY, RC, SV, VE endemic, exotic, established, no control – reduces pest, NC, ABC
Trichogramma terani Velásquez and Terán (Hymenoptera: Trichogrammatidae)	VE, endemic, role not quantified, NC
Trichogrammatoidea armigera (Nagaraja) (Hymenoptera: Trichogrammatidae)	AR, exotic, no further info, DM exotic, not established
Trichogrammatoidea bactrae Nagaraja (Hymenoptera: Trichogrammatidae)	AR, exotic, not established insufficient control ABC, BB, exotic, not established, MX, exotic, tried in ABC, no data, PE exotic, reduces pest, ABC
Trichogrammatoidea cryptophlebiae Nagaraja (Hymenoptera: Trichogrammatidae)	CR, endemic, reduces pest, but too expensive to use in ABC

Trichogrammatoidea robusta Nagaraja *(*as *T. nana* Zhnt.*)* (Hymenoptera: Trichogrammatidae)	BZ, exotic, not established
Trichogrammatoidea sp. (Hymenoptera: Trichogrammatidae)	BO, endemic, reduces pest, NC
Trichopoda giacomelli (Blanchard) (Diptera: Tachinidae)	AR endemic, role not quantified, NC
Trichopoda pennipes (F.) (Diptera: Tachinidae)	RC exotic, not established
Trichopoda pilipes F. (Diptera: Tachinidae)	RC exotic, not established
Trichopoda sp. (Diptera: Tachinidae)	VE, endemic, role not quantified, NC
Trichopria drosophilae (Hymenoptera: Diapriidae)	MX, endemic, role not quantified, NC
Trichospilus diatraeae (C. and M.) (Hymenoptera: Eulophidae)	JM, endemic, role not quantified, NC
Trichospilus pupivora (= *pupivorus*) Ferriere (Hymenoptera: Eulophidae)	BB, exotic, established, role not quantified, CBC
Trioxys pallidus (Haliday) (Hymenoptera: Braconidae)	CL, exotic, established, good control, CBC
Trissolcus basalis (Wollaston) (Hymenoptera: Platygastridae)	AR, BO, BR, PA, PY, RC, TT, UY, endemic and exotic, reduces pest, ABC
Trissolcus brochymenae Ashmead (Hymenoptera: Platygastridae)	BO, UY endemic, role not quantified, NC
Trissolcus leviventris (Cameron)(=*Dissolcus paraguayensis* Brethes)(Hymenoptera: Platygastridae)	PY endemic, role not quantified, NC
Trissolcus mitsukurii (Ashm.) (Hymenoptera: Platigastridae)	RC exotic, not established
Trissolcus sp. (Hymenoptera: Platygastridae)	PY endemic, role not quantified, NC
Trissolcus spp. (Hymenoptera: Platygastridae)	BO, endemic, reduces pest, ABC
Trissolcus teretis Johnson (Hymenoptera: Platygastridae)	UY endemic, role not quantified, NC
Trissolcus urichi Crawford (Hymenoptera: Platygastridae)	BO, UY endemic, role not quantified, NC
*Trybliographa dac*i Weld (Hymenoptera: Cynipidae)	CR, EC endemic, role not quantified, NC
Urosigalphus eulechriopis Cushman (Hymenoptera: Braconidae)	PY endemic, role not quantified, NC
Utetes anastrephae (Viereck) (Hymenoptera: Braconidae)	DR, MX endemic, role not quantified, NC
Venturia ovivenans nov. spec Zwart (Hymenoptera: Ichneumonidae)	SR endemic, role not quantified, NC
Venturia sp. (Hymenoptera: Ichneumonidae)	BO endemic, role not quantified, NC
Voria sp. (Diptera: Tachinidae)	PY endemic, role not quantified, NC
Voria spp. (Diptera: Tachinidae)	UY endemic, role not quantified, NC
Winthemia pinguioides (Townsend) (Diptera: Tachinidae)	VE, endemic, role not quantified, NC
Winthemia pinguis (Fabricius) (Diptera: Tachinidae)	SR, endemic, reduces pest, NC
Winthemia sp. (Diptera: Tachinidae)	BB, JM, RC, PY endemic, reduces pest, NC
Winthemia sp. nr. *pinguis* F. (Diptera: Tachinidae)	BB endemic, role not quantified, NC
Winthemia sp. similar to *W. pyrrhopyga* (Wied) (Diptera: Tachinidae)	BB endemic, role not quantified, NC

Winthemia spp. (Diptera: Tachinidae)	PY endemic, role not quantified, NC
Xenoencyrtus niger Riek (Hymenoptera: Encyrtidae)	RC, exotic, not established
Xenostigmus bifasciatus Ashmed (Hymenoptera: Braconidae)	BR, exotic, reduces pest, CBC
Yahuartachina sp.n. (Diptera: Tachinidae)	SR endemic, role not quantified, NC
Zaeucoila sp. (Hymenoptera: Figitidae)	FA, endemic, role not quantified, NC
Zaglyptus sp. Forster (Hymenoptera: Ichneumonidae)	DR, endemic, role not quantified, NC
Zagrammosoma multilineata Ashm (Hymenoptera: Eulophidae)	PR, endemic, role not quantified, NC
Zagrammosoma sp. (Hymenoptera: Eulophidae)	DR, endemic, role not quantified, NC
Zagrammosoma spp. (Hymenoptera: Eulophidae)	GT, endemic, reduces pest, NC
Zelomorpha sp. (Hymenoptera:Braconidae)	SR, endemic, role not quantified, NC

Country abbreviations: AR = Argentina, BB = Barbados, Belize = BZ, Bolivia = BO, Brazil = BR, Chile = CL, Colombia = CO, Costa Rica = CR, Cuba = CU, Dominica = DM, Dominican Republic = DO, Ecuador = EC, El Salvador = SV, French Guiana, Guadeloupe and Martinique = FA, Guatemala = GT, Guyana = GY, Haiti = HT, Honduras = HN, Jamaica = JM, Mexico = MX, Nicaragua = NI, Panama = PA, Paraguay = PY, Peru = PE, Puerto Rico = PR, Remaining Caribbean Islands = RC, Suriname = SR, Trinidad and Tobago = TT, Uruguay = UY, Venezuela = VE
