# Supplementary material for: Benefits and Risks of Native and Exotic Biological Control Agents Used in Latin America and the Caribbean: Performance of 1099 Arthropod Natural Enemies
Source: Neotrop Entomol. 2026 Jul 28;55(1):69. doi: 10.1007/s13744-026-01412-8 (PMC13415494; doi:10.1007/s13744-026-01412-8)
Supplement: Supplementary file 3 — (PDF 469 KB) [file 13744_2026_1412_MOESM3_ESM.pdf]

**Table SI3. Benefits and risks of endemic and exotic biological control agents used in Latin America and the Caribbean: performance of 1099 arthropod natural enemies. Joop C. van Lenteren, Vanda H. P. Bueno, Wageningen University, Laboratory of Entomology, Department of Plant Sciences, 6700 AA, Wageningen, The Netherlands, joop.vanlenteren@wur.nl**<br><br>**Predators mentioned in van Lenteren et al. 2020: "Biological Control In Latin America and The Caribbean: Its Rich History And Bright Future"**	
**Predator species**	**Country where recorded, endemic/exotic, role in biocontrol**
Acanthops falcata Stål (Mantodea:Acanthopidae)	VE endemic, generalist predator, role not quantified, NC
Acontiothespis brevipennis (Saussure) (Mantodea: Acontistidae)	PY endemic, generalist predator, role not quantified, NC
Acontiothespis concinna (Perty) (Mantodea: Acontistidae)	PY endemic, generalist predator, role not quantified, NC
Acontiothespis multicolor (Saussure) (Mantodea: Acontistidae)	VE endemic, generalist predator, role not quantified, NC
Adalia bipunctata L. (Coleoptera: Coccinellidae)	32
Allograpta (Diptera: Syrphidae)	FA endemic, role not quantified, NC
Allograpta (*Fazia*) CR-2 aff. *hians* (Enderlein) (Diptera: Syrphidae)	CO endemic, role not quantified, NC
Allograpta exotica (Wiedemann) (Diptera: Syrphidae)	PY, VE endemic, role not quantified, NC
Amazona vittata Boddaert (Psittaciformes: Psittacidae)	PR, endemic, parrot,
*Amblydromalus limonicus (*Garman & McGregor) (Acari: Phytoseiidae)	MX, endemic, good control of thrips, ABC, NC
Amblyseius (Acari: Phytoseiidae)	BB, DO, exotic, good control of red palm mite, established, CBC
Amblyseius chungas Denmark & Muma (Acari: Phytoseiidae)	PE, endemic, good control of mites, ABC, NC
Amblyseius herbicolus (Chant) (Acari: Phytoseiidae)	CO, endemic, good control of mites, ABC, NC
Amblyseius largoensis (Muma) (Acari: Phytoseiidae)	CU, DO, GY, JM, MX, PE, RC, endemic, good control of mites, ABC, NC
Amblyseius obtusus (Koch) (= *Amblyseiopsis musae* Garman) (Acari: Phytoseiidae)	DO, endemic, role not quantified, NC
Amblyseius sp. (Acari: Phytoseiidae)	BB, EC, DO, exotic, good control of pest, ABC and CBC
Amblyseius swirskii Athias-Henriot (Acari: Phytoseiidae)	EC, HN, UY, exotic, good control of pest, ABC
Amblyseius tamatavensis Blommers (Acari: Phytoseiidae)	BR, DO, endemic, role not yet quantified, ABC
Ameiva atrigularis (L.) (Squamata: Teiidae)	TT, endemic, lizard, insect predator, killed by mongoose, role not quantified, NC

Ameiva spp. (Squamata: Teiidae)	BB, RC, endemic, lizard (killed by mongoose), role not quantified, NC
Anolis (Squamata: Dactyloidea)	PR, RC, endemic, lizard, role not quantified, NC and ConsBC
Anovia circumclusa (Gorham) (Coleoptera: Coccinellidae)	HT, endemic, reduces pest, CBC, NC
Anovia punica Gordon (Coleoptera: Coccinellidae)	CO, HT, endemic, reduces pest, CBC, NC
Aphidoletes aphidimyza (Rondani) (Diptera: Cecidomyiidae)	CO, MX,VE, exotic, good kill of pest, ABC
Apiomerus apicalis Burmeister (Hemiptera: Reduviidae)	PY, endemic, role not quantified, NC
Apiomerus lanipes (F.) (Hemiptera: Reduviidae)	PY, endemic, role not quantified, NC
Apiomerus sp. (Hemiptera: Reduviidae)	PY, endemic, role not quantified, NC
Argiope trifasciata (Forskal) (Araneae: Araneidae)	JM, endemic, generalist predator, role not quantified, NC
Arilus carinatus (Forster *)* (Hemiptera: Reduviidae)	PY, endemic, role not quantified, NC
Arilus sp. aff. cristatus (L.) (Hemiptera: Reduviidae)	PY, endemic, role not quantified, NC
Asarkina ericetorum (Fabricius) (Diptera: Syrphidae)	TT, endemic, role not quantified, NC
Aspisoma sp. (Coleoptera: Lampyridae)	PY, endemic, role not quantified, NC
Atheta coriaria Kraatz (Coleoptera: Staphylinidae)	MX, exotic, reduces pest, ABC
Atopozelus opsimus Elkins (Hemiptera: Reduviidae)	PY, endemic, role not quantified, NC
Atrachelus cinereus crassicornis (Burmeister) (Hemiptera: Reduviidae)	PY, endemic, role not quantified, NC
Azya luteipes Mulsant (Coleoptera: Coccinellidae)	PY, endemic, role not quantified, NC
Azya orbigera Mulsant (Coleoptera: Coccinellidae)	AR, CO, RC, TT, VE, endemic, role not quantified, NC
Azya trinitatis Marshall (Coleoptera: Coccinellidae)	SR, endemic, good control, NC
Azya sp. (Coleoptera: Coccinellidae)	RC, endemic+exotic, role not quantified, NC
Baccha clavata (Fabricius)	DR, endemic, role not quantified, NC
Baccha sp. (Diptera: Syrphidae)	FA, SR, endemic, role not quantified, NC
Blaesoxipha aculeata Aldrich (Diptera: Sarcophagidae)	AR, exotic, not established
Blaesoxipha atlanis Aldrich (Diptera: Sarcophagidae)	AR, exotic, not established
Blaesoxipha australis Blanchard (Diptera: Sarcophagidae)	AR, exotic, not established
Blaesoxipha caridei (Brétes) (Diptera: Sarcophagidae)	AR, exotic, established, role not quantified, CBC
Blaesoxipha filipjevi Rhod (Diptera: Sarcophagidae)	BB, exotic, not established
Blaesoxipha hunteri (Hough) (Diptera: Sarcophagidae)	AR, exotic, not established
Blaesoxipha neuquenensis Blanchard (Diptera: Sarcophagidae)	AR, exotic, established, role not quantified, CBC

Blaesoxipha opifera Coquillett (Diptera: Sarcophagidae)	AR, exotic, not established
Blaesoxipha reversa Aldrich (Diptera: Sarcophagidae)	AR, exotic, not established
Brachicantha sp. (Coleoptera; Coccinellidae)	BO, endemic, reduces pest, NC
Brachygastra lecheguana (Latreille) (Hymenoptera: Vespidae)	PY, endemic, role not quantified, NC
Buenoa scimitra Bare (Hemiptera: Notonectidae)	MX, endemic, reduces pest, ABC, NC
Bufo marinus L. (Anura: Bufonidae)	BB, CR,FA, GY, RC, exotic, established, reduced pest, but also neg effects by eating beneficial insects, CBC
Bufo spp. (Anura: Bufonidae)	BO, endemic, established, role not quantified, CBC, negat eff
Calosoma alternans Fabricius (Coleoptera: Carabidae)	VE, endemic, role not quantified, NC
Calosoma granulatum Perty (Coleoptera: Carabidae)	PY, endemic, role not quantified, NC
Calosoma sp. (Coleoptera: Carabidae)	BO, PY, endemic, role not quantified, NC
Camponotus sexguttatus (Fabricius) (Hymenoptera: Formicidae)	FA, endemic, role not quantified, NC
*Campyloneuropsis infumatus (*Carvalho) (Hemiptera: Miridae)	BR, endemic, reduces pest, NC
Castolus plagiaticollis Stål.(Hemiptera:Reduviidae)	TT, exotic, ?established, role not quantified, CBC/ABC
Castrida alternans granulatum (Perty) (Coleoptera: Carabidae)	PY, endemic, role not quantified, NC
Catana clauseni Chapin (Coleoptera: Coccinellidae)	CU, JM, RC, exotic, not established
Ceraeochrysa cf. *claveri* (Navás) (Neuroptera: Chrysopidae)	CO, endemic, role not quantified, NC
Ceraeochrysa cincta (Schneider) (Neuroptera: Chrysopidae)	MX, endemic, lab rearing PE, endemic good control of pest, ABC, NC
Ceraeochrysa claveri (Navás) (Neuroptera: Chrysopidae)	MX, endemic, good control of pest, ABC, NC
Ceraeochrysa cubana (Hagen) (Neuroptera: Chrysopidae)	FA, endemic, role not quantified, NC
Ceraeochrysa sp. (Neuroptera: Chrysopidae)	CO, endemic, reduces pest, ABC , CR endemic, not quantif, NC
Ceraeochrysa valida (Banks) (Neuroptera: Chrysopidae)	CR, MX , endemic, good control, ABC
Cheilomenes sexmaculata (Fabricius) (Coleoptera: Coccinellidae)	CO, endemic, role not quantified, NC
Chilocorus bipustulatus L. (Coleoptera: Coccinellidae)	RC, exotic, ?established
Chilocorus bivulnerus (Mulsant) (Coleoptera: Coccinellidae)	UY, exotic, established, good pest control, CBC
Chilocorus cacti L. (Coleoptera: Coccinellidae)	CU, DO, DM, FA, JM, RC, VE, endemic, exotic, good control, ABC, CBC, NC
Chilocorus cf. *cacti* (L.) (Coleoptera: Coccinellidae)	CO, endemic, role not quantified, NC
Chilocorus nigritus (F.) (Coleoptera: Coccinellidae)	FA, endemic, role not quantified, NC
Chilocorus stigma (Say) (Coleoptera: Coccinellidae)	UY, exotic, died during shipment

Chnoodes sp. (Coleoptera: Coccinellidae)	RC, exotic, not established, TT endemic, role not quantified, NC
Chnoodes spp. (Coleoptera: Coccinellidae)	JM, exotic, ?established, role not quantified, CBC
Chrysocerca (Neuroptera: Chrysopidae)	FA, endemic, role not quantified, NC
Chrysopa (Neuroptera: Chrysopidae)	FA, endemic, role not quantified, NC
Chrysopa sp. (Neuroptera: Chrysopidae)	CO, CR, DO, PA, RC, SV, endemic, control of pest, NC
Chrysopa sp. (Neuroptera: Chrysopidae)	GT, exotic, control of pest, ABC
Chrysopa sp. near Silvana Navas (Neuroptera: Chrysopidae)	SR, endemic, role not quantified, NC
Chrysopa spp. (Neuroptera: Chrysopidae)	CU endemic, reduces pest, ABC, NC, DO, SR, endemic, role not quantified, NC
Chrysoperla (Neuroptera: Chrysopdiae)	CL, ?exotic, reduces pest, ABC
Chrysoperla asoralis (Banks) (Neuroptera: Chrysopidae)	PE, endemic, reduces pest, ABC
Chrysoperla carnea (Stephens) (Neuroptera: Chrysopidae)	CO, CR, Nicaragua, PE, VE, exotic reduces pest, ABC, MX, endemic, reduces pest, ABC, NC
Chrysoperla comanche (Banks) (Neuroptera: Chrysopidae)	MX, endemic, reduces pest, ABC
Chrysoperla externa (Hagen) (Neuroptera: Chrysopidae)	AR, BB, FA, MX, NI, PE, VE, exotic, role not quantified or reduces pest, ABC, NC
Chrysoperla rufilabris (Burmeister) (Neuroptera: Chrysopidae)	CO, MX, endemic, reduces pest, ABC, NC
Chrysoperla sp. (Neuroptera: Chrysopidae)	BB, CO, DO, PE, PY, endemic, role not quantified, NC
Chrysoperla spp. (Neuroptera: Chrysopidae)	CL, CO, MX, endemc, reduces pest, ABC, NC
Chrysopodes (Neuroptera: Chrysopidae)	FA, endemic, role not quantified, NC
Cicindela sp. (Coleoptera: Carabidae)	PY, endemic, role not quantified, NC
Cladis nitidula F. (Coleoptera: Coccinellidae)	FA, RC, exotic, role not quantified, vars islands, exotic, reduced pest, CBC
Coccidophilus cariba Gordon (Coleoptera: Coccinellidae)	RC, exotic,?established, role not quantified, ABC
Coccidophilus citricola Brethes (Coleoptera: Coccinellidae)	CL, PE, RC, exotic, role not quantified, CBC
Coccidophilus sp. (Coleoptera: Coccinellidae)	SR, endemic, role not quantified, CBC, NC
Coccinella septempunctata (L.) (Coleoptera: Coccinellidae)	AR, FA, RC, exotic, established, role not quantified to controls pest, ABC
Coccinellina sp. (Coleoptera: Coccinellidae)	PY, endemic, role not quantified, NC
Coelophora inaequalis F. (Coleoptera: Coccinellidae)	FA, endemic, role not quantified, NC
Coenosia attenuata Stein (Diptera: Muscidae)	EC endemic, good control, ABC, NC
Coleomegilla cubensis Casey (Coleoptera: Coccinellidae)	CU, DO, endemic, reduces pest, ABC, NC

Coleomegilla maculata De Geer (Coleoptera: Coccinellidae)	EC, FA, JM, MX, PA, PY, SR, TT, VE, endemic, good control ABC, NC
Coleomegilla quadrifasciata (Schoenherr) (Coleoptera: Coccinellidae)	PY, endemic, role not quantified, NC
Condylostylus graenicheri (Van Duzee) (Diptera: Dolichopodidae)	PY, endemic, role not quantified, NC
Condylostylus similis (Aldrich) (Diptera: Dolichopodidae)	PY, endemic, role not quantified, NC
Condylostylus sp. (Diptera: Dolichopodidae)	DO, PY, endemic, role not quantified, NC
Conomyrma sp. (Hymenoptera: Formicidae)	DO, endemic, role not quantified, NC
Copestylum isabellina (Williston) (Diptera: Syrphidae)	VE, endemic, role not quantified, NC
Copestylum musicanum (Curran) (Diptera: Syrphidae)	VE, endemic, role not quantified, NC
Copestylum punctiferum (Bigot) (Diptera: Syrphidae)	VE, endemic, role not quantified, NC
Copestylum rurale Curran (Diptera: Syrphidae)	VE, endemic, role not quantified, NC
Copestylum sica (Curran) (Diptera: Syrphidae)	VE, endemic, role not quantified, NC
Coranus spiniscutus Reuter (Hemiptera: Reduviidae)	BB, exotic, not established,
Cosmoclopius sp. (Hemiptera: Reduviidae)	PY, endemic, role not quantified, NC
Cosmoclopius sp. aff. *annulosus* Stål (Hemiptera: Reduviidae)	PY, endemic, role not quantified, NC
Crematogaster brevispinosa Mayr (Hymenoptera: Formicidae)	JM, endemic, did not control the pest, ConsBC, NC
Crematogaster spp. (Hymenoptera: Formicidae)	CO, CR, endemic, reduces pest, ConsBC, NC
Crematogaster torosa (Mayr) (Hymenoptera: Formicidae)	CR, endemic, reduces pest, NC
Crotophaga ani L. (Cuculiformes: Cuculidae)	SR, TT, endemic, good control of pest, NC birds
Crotophaga major Gmelin (Cuculiformes: Cuculidae)	SR, , endemic, good control of pest, NC birds
Cryptognatha affinis (Coleoptera: Coccinellidae)	TT, RC, exotic, ?established, role not quantified, CNC
Cryptognatha auriculata Muls. (Coleoptera: Coccinellidae)	SR, VE, endemic, good control, NC
Cryptognatha flaviceps (Crotch) (Coleoptera: Coccinellidae)	RC, exotic, ?established, role not quantified, CBC
Cryptognatha nodiceps Marshall (Coleoptera: Coccinellidae)	BB, DO, JM, RC, TT, exotic, established/not everywhere, reduces pest, CBC
Cryptognatha simillima Sic. (Coleoptera: Coccinellidae)	BB, RC, exotic, ?established, role not quantified, CBC
Cryptolaemus affinis Crotch (Coleoptera: Coccinellidae)	RC, exotic, ?established, role not quantified, CBC
Cryptolaemus montrouzieri Mulsant (Coleoptera: Coccinellidae)	BB, BR, BZ, CL, CR, CU, DO, FA, GY, MX, PE, PR, PY, RC, SR, TT, VE, exotic, established, reduces/controls pest, CBC
Crysso sp. (Araneae: Araneidae)	CR, endemic, role not quantified, NC
Curinus colombianus Chapin (Coleoptera: Coccinellidae)	CO, endemic, role not quantified, NC

Cybocephalus nipponicus Endrody-Younga (Coleoptera: Cybocephalidae)	BB, DO, EC, MX, RC, exotic, established, good control, CBC, ABC
Cybocephalus sp. (Coleoptera: Cybocephalidae)	TT, exotic, reared and shipped to other locations
Cycloneda conjugata (Mulsant) (Coleoptera: Coccinellidae)	PY, endemic, role not quantified, NC
Cycloneda oculata (Thumberg) (Coleoptera: Coccinellidae)	UY, exotic, not established
Cycloneda sanguinea (L.) (Coleoptera: Coccinellidae)	BB, BO, CO, CR, DO, EC, FA, JM, MX, PA, PY, SR, VE, endemic, good control, NC, ABC-ConsBC,
Cycloneda sanguinea limbifer (Casey) (Coleoptera: Coccinellidae)	CU, endemic, good control, ABC, NC
Cyrtorhinus fulvus Knight (Hemiptera: Miridae)	JM, endemic, role not quantified, NC
Dactylosternum abdominale (Fabricius) (Coleoptera: Hydrophilidae)	CU, exotic, no control JM exotic good control ConsBC, RC exotic, role not quantified
Dactylosternum hydrophiloides (MacLeay) (Coleoptera: Hydrophilidae)	CU, JM, RC, exotic, established, good control, ConsBC
Dactylosternum sp. (Coleoptera: Hydrophylidae)	DM, endemic, role not quantified, NC, RC, exotic, ?established, role not quantified
Dactylosternum subdepressum Lap. (Coleoptera: Hydophilidae)	RC, exotic, ?established, role not quantified
Delphastus (Coleoptera: Coccinellidae)	SR, endemic, role not quantified, NC
Delphastus argentinicus Nunenmacher (Coleoptera: Coccinellidae)	PY, endemic, role not quantified, NC
Delphastus catalinae Horn (Coleoptera: Coccinellidae)	CO, JM endemic, good control, ABC, NC
Delphastus pallidus Le Conte (Coleoptera: Coccinellidae)	FA, endemic, role not quantified, NC
Delphastus pusillus (LeConte) (Coleoptera: Coccinellidae)	DO, endemic, good control, ABC, NC, FA, MX exotic, controls pest, ABC
Delphastus quinculus Gordon (Coleoptera: Coccinellidae)	CO, endemic, role not quantified, NC
Delphastus sp. (Coleoptera: Coccinellidae)	CR, endemic, reduces pest, NC, SV endemic, insufficient, ABC
Diomus seminulus (Mulsant) (Coleoptera: Coccinellidae)	CO, endemic, role not quantified, NC
Diomus sp. (Coleoptera: Coccinellidae)	JM, exotic, ?established, RC, endemic, role not quantified, NC
Diomus sp. aff. *Tantillus* (Coleoptera: Coccinellidae)	PY, endemic, role not quantified, NC
Doru lineare (Eschsholtz) (Dermaptera: Forficulidae)	PY, endemic, role not quantified, NC
Doru luteipes (Scudder) (Dermaptera: Forficulidae)	PY, endemic, role not quantified, NC
Doru sp. (Dermaptera: Forficulidae)	CR, endemic, reduces pest, NC, PY , endemic, role not quantified, NC
Engytatus modestus (Distant) (Hemiptera: Miridae)	DO, endemic, reduces pest, NC, also mentions risk but not data
Engytatus varians (Distant) (Hemiptera: Miridae)	BR, endemic, reduces pest, ABC, NC
Eriopis chilensis (Hofmann) (Coleoptera: Coccinellidae)	CL, endemic, reduces pest, ABC, NC

Eriopis (Naemia) connexa (Germar) (Coleoptera: Coccinellidae)	AR, endemic, reduces pest, ConsBC, NC, PY, endemic, role not quantified, NC
Eriopis sp. (Coleoptera: Coccinellidae)	BO, endemic, reduces pest, NC
Euseius (=*Amblyseius*) *victoriensis* Womersly (Acari: Phytoseiidae)	MX, PE, exotic, good control, ABC
Euseius concordis (Chant) (Acari: Phytoseiidae)	PE, exotic, good control, ABC
Euseius sp. (Acari: Phytoseiidae)	PE, exotic, good control, ABC
Euseius scutalis AUTHOR, (Acari: Phytoseiidae)	PE, exotic, good control, ABC
Euseius stipulatus (Athias-Henriot) (Acari: Phytoseiidae)	MX, PE, exotic, good control, ABC, CBC
Euseius (=Amblyseius) victoriensis Womersly (Acari: Phytoseiidae)	MX, PE, exotic, good control, ABC, CBC
Exochonus bisbinotatus Gorham (Coloeoptera: Coccinellidae)	RC, exotic, not established
Exochomus marginipennis (LeConte) (Coleoptera: Coccinellidae)	MX, exotic, reduces pest, ABC/CBC
Exochomus sp. (Coleoptera: Coccinellidae)	TT, shipped from TT to other locations, no further info
Exoplectra dubia Crotch (Coleoptera: Coccinellidade)	RC, exotic, not established
Feltiella acarisuga (Vallot) (Diptera: Cecidomyiidae)	MX, exotic, good control of pest, ABC
Forficula auricularia L. (Dermaptera: Forficulidae)	PY, endemic, role not quantified, NC
Franklinothrips vespiformis (Crawford) (Thysanoptera: Aeolothripidae)	BB, DO, FA, PY, endemic, reduces pest, ABC, NC
Fundiseius cesi (Muma) (Acari: Phytoseiidae)	JM, exotic, role not quantified, ABC
Galendromus (=Typhlodromus) occidentalis (Nesbitt) (Acari: Phytoseiidae)	CO, MX, exotic, reduces pest, ABC
Galendromus helveolus (Chant) (Acari: Phytoseiidae)	MX, exotic, reduces pest, ABC
Gambusia affinis Baird & Girard (Cyprinodontiformes: Poeciliidae)	CO, MX, predatory fish, endemic, good control, ABC, NC
Gasteracantha cancriformis L. (Araneae: Araneidae)	CR, endemic, reduces pest, NC
Geocoris callosullus Berg (Hemiptera: Geocoridae)	PE, endemic, reared in PE, but no further info, ABC, NC
Geocoris punctipes (Say) (Hemiptera: Geocoridae)	MX, endemic, reduces pest, ABC, NC
Geocoris sp. (Hemiptera: Geocoridae)	MX, endemic, reduces pest, ABC, NC, PY, endemic, role not quant, NC
Geocoris ventralis (Fieber) (Hemiptera: Geocoridae)	PY endemic, role not quant, NC
Graptocleptes bicolor (Burmeister) (Hemiptera: Reduviidae)	PY endemic, role not quant, NC
Gymnopolybia sp. (Hymenoptera: Vespidae)	PY endemic, role not quant, NC
Habronathus sp. (Arachnida: Araneae)	JM endemic, reduces pest, NC
Harmonia (= *Leis*) sp. (Coleoptera: Coccinellidae)	CU, exotic, insufficient control, CBC

Harmonia axyridis (Pallas) (Coleoptera: Coccinellidae)	AR, CL, CO, MX, PY, VE, exotic, established, reduces pest, ABC, negative effects mentioned in CL
Harmonia sp. (Coleoptera: Coccinellidae)	32, exotic, established,
Hemerobius tolimensis Banks (Neuroptera: Hemerobiidae)	BO, endemic, role not quantified, NC
Herpestes auropunctatus Hodgson (Carnivora: Herpestidae)	BB, DO, JM, PR, RC, TT, exotic, established, negative side effects
Hippodamia convergens Guérin-Méneville (Coleoptera: Coccinellidae)	BO, CO, CU, DO, EC, MX, NI, PA, PE, PY, VE, endemic, reduces pest, NC/CBC/ABC/ConsBC
Hippodamia quinquesignata Kirby (Coleoptera: Coccinellidae)	BR, exotic, role not quantified, ABC
Hippodamia sp. (Coleoptera: Coccinellidae)	BO, endemic, reduces pest, NC
Hiranetis sp. (Hemiptera: Reduviidae)	PY, endemic, role not quantified, NC
Hister bruchi Lewis (Coleoptera: Histeridae)	AR, exotic, ?established, role not quantified
Hololepta (=*Leionota*) *quadridentata* (Olivier) (Coleoptera:Histeridae)	BB, DM, RC, exotic, not established
Hyperaspis distinguenda (Muls.) (Coleoptera: Coccinellidae)	BB, exotic, ? established, no control
Hyperaspis donzeli (Muls.) (Coleoptera: Coccinellidae)	BB, exotic, ? established, no control RC, exotic, not established
Hyperaspis festiva Muls (Coleoptera: Coccinellidae)	PY, endemic, role not quantified, NC, SR endemic, role not quantified, NC
Hyperaspis jucunda (Muls.) (Coleoptera: Coccinellidae)	BB, exotic, ? established, no control, RC, exotic, not established, TT
Hyperaspis notata Mulsant (Coleoptera: Coccinellidae)	PY, endemic, role not quantified, NC
Hyperaspis onerata (Mulsant) (Coleoptera: Coccinellidae)	PE, endemic, mass reared but no info were used, ABC, NC
Hyperaspis sp. (Coleoptera: Coccinellidae)	BB, , JM, PY endemic, role not quantified, NC, RC, exotic, ?established, no control, PY endemic, role not quantified, NC
Hyperaspis spp. (Coleoptera: Coccinellidae)	BO, endemic, reduces pest, NC
Hyperaspis trilineata Mulsant (Coleoptera: Coccinellidae)	RC, exotic, established, reduces pest, CBC, VE, endemic, reduces pest, NC
Hypoaspis sp. (Acari: Laelapidae)	PA, endemic, role not quantified
Ignelater luminosus (Illiger) (Coleoptera: Elateridae)	BB, endemic, established, insufficient control, ABC, NC
Iphiseiodes zuluagai Denmark & Muma (Acari: Phytoseiidae)	CO, endemic, reduces pest, ABC, NC
Iphiseius (=Amblyseius) degenerans (Berlese) (Acari: Phytoseiidae)	AR, CO, MX, exotic orig Belgium, good control, ABC
Labidura riparia (Pallas) (Dermaptera: Labiduridae)	BO, endemic, reduces pest, NC
Lasioseius (Acari: Blattisociidae)	DO, endemic, role not quantified, NC
Lasioseius chaudhrii (Wu & Wang) (Acari: Blattisociidae)	DO endemic, role not quantified, NC

Lasioseius dominicensis n. sp Abo-Shnaf, Sanchez & De Moraes (Acari: Blattisociidae)	DO endemic, role not quantified, NC
Lasioseius oryzae n. sp. Abo-Shnaf, Sanchez & De Moraes (Acari: Blattisociidae)	DO endemic, role not quantified, NC
Lasioseius prorsoperitrematus n. sp. Abo-Shnaf, Sanchez & De Moraes (Acari: Blattisociidae)	DO endemic, role not quantified, NC
Lasioseius sanchezensis n. sp. Abo-Shnaf, Sanchez & De Moraes (Acari: Blattisociidae)	DO endemic, role not quantified, NC
Lebia sp. (Coleoptera: Carabidae)	PY endemic, role not quantified, NC
Lejops mexicanus (Macquart) (Diptera: Syrphidae)	VE endemic, role not quantified, NC
Lestodiplosis sp. (Diptera: Cecidomyiidae)	BB, endemic, good control, NC
Leucochrysa floridana (Banks) (Neuroptera: Chrysopidae)	FA endemic, role not quantified, NC
Leucopis bella Loew (Diptera: Chamaemyiidae)	RC, exotic, good control, CBC
Leucopodella sp. (Diptera: Syrphidae)	CO endemic, role not quantified, NC
Lindorus lophanthae Casey (Coleoptera: Coccinellidae)	AR, PE, UY, exotic, established, good control, CBC
Lioscymnus diversipes Champ. (Coleoptera: Coccinellidae)	RC, exotic, not established
Lotis neglecta Muls. (Coleoptera: Coccinellidae)	RC exotic, not established
Lotis nigerimma Csy (Coleoptera: Coccinellidae)	RC exotic, not established
Lycosa spp. (Araneae: Lycosidae)	JM, endemic, reduces pest, NC
Macrolophus basicornis (Stal) (Hemiptera: Miridae)	BR, endemic, reduces pest, ABC, NC
Macrolophus nr praeclarus Distant (Hemiptera: Miridae)	FA, endemic, role not quantified, NC
Macrolophus praeclarus (Distant) (Hemiptera: Miridae)	DO, endemic, reduces pest, NC, may cause plant damage, no data
Macrocyclops (Copepoda: Cyclopidae)	RC, endemic, role not quantified, NC
Megacephala carolina (Linnaeus) (Coleoptera: Carabidae)	BO endemic, reduces pest, NC
Megacephala chilensis (Laporte de Castelnau) (Coleoptera: Carabidae)	BO endemic, reduces pest, NC
Megacephala sp. (Coleoptera: Carabidae)	PY endemic, role not quantified, NC
Melaleucopis simmondsi Sabrosky (Diptera: Chamaemyiidae)	RC endemic, role not quantified, NC
Menochilus sexmaculatus (F.) (Coleoptera: Coccinellidae)	VE endemic, role not quantified, NC
Mesocyclops (Copepoda: Cyclopidae)	RC endemic, good control, ABC, NC
Mesocyclops longisetus (Thiébaund) (Copepoda: Cyclopidae)	CO, endemic, good control, ABC, NC
Mesocyclops sp. (Copepoda: Cyclopidae)	RC, endemic, good control, ABC, NC
Mesograpta basilaris Wiedemann (Diptera: Syrphidae)	VE, endemic, role not quantified, NC
Mesograpta sp. (Diptera: Syrphidae)	SR endemic, role not quantified, NC

Metacanthus tenellus Stal (Hemiptera: Berytidae)	PE, endemic, mass reared, no info about control, ABC, NC
Micranchenus lineola (F) (Hemiptera: Reduviidae)	SR, endemic, role not quantified, NC
Montandiola sp. (Hemiptera: Anthocoridae)	PA endemic, role not quantified, NC
Montina confusa (Stal) (Hemiptera: Reduviidae)	BR, endemic, reduces pest, ABC, NC
Nabis capsiformis (Germar) (Hemiptera: Nabidae)	FA, PY endemic, role not quantified, NC
Nabis sordidus Reuter (Hemiptera: Nabidae)	TT, endemic, reduces pest, ABC, NC
Nabis sp. (Hemiptera: Nabidae)	PY endemic, role not quantified, NC
Nausigaster meridionalis Townsend (Diptera: Syrphidae)	VE, endemic, role not quantified, NC
Neoseiulus (= Amblyseius) californicus (McGregor) (Acari: Phytoseiidae)	AR, BR,CO, EC, JM, MX, PE, endemic, established, good control, ABC
Neoseiulus (Acari: Phytoseiidae)	DO, endemic, role not quantified, NC
Neoseiulus (Amblyseius) barkeri Hughes (Acari: Phytoseiidae)	CO, MX, endemic, reduces pest, ABC
Neoseiulus (Amblyseius) cucumeris (Oudemans) (Acari: Phytoseiidae)	CO, MX, HN, endemic, ireduces pest, ABC, NC
Neoseiulus anonymus (Chant & Baker) (Acari: Phytoseiidae)	CO, endemic, reduces pest, ABC, NC
Neoseiulus baraki Athias-Henriot (Acari: Phytoseiidae)	PA, endemic, role not quantified, NC
Neoseiulus barabensis Athias-Henriot (Acari: Phytoseiidae)	PA endemic, role not quantified, NC
Neoseiulus longispinosus (Evans) (Acari: Phytoseiidae)	CU, HN, endemic, reduces pest, ABC
Nephaspis (Coleoptera: Coccinellidae)	RC, exotic, established? role not determined
Nephaspis amnicola Wingo (Coleoptera: Coccinellidae)	BB, RC, exotic, established, role not quantified, CBC
Nephaspis bicolor Gordon (Coleoptera: Coccinellidae)	TT, mass reared and shipped from TT
Nephaspis nigra Gordon (Coleoptera: Coccinellidae)	BB, RC exotic, established, role not quantified, CBC
Nephaspis sp. (Coleoptera: Coccinellidae)	CR, endemic, reduces pest, NC
Nephus regularis Sicard (Coleoptera: Coccinellidae)	RC exotic, ?established, no control
Nephus sp. (Coleoptera: Coccinellidae)	BB, RC, exotic, ?established, no control, PY endemic, role not quantified, NC
Nesidiocoris (= *Cyrtopeltis*) *tenuis* (Reuter) (Hemiptera: Miridae)	DO, FA endemic, role not quantified, NC
Nodita sp. (Neuroptera: Chrysopidae)	SR endemic, role not quantified, NC
Notiobia laevis bolivianus (Van Emdem) (Coleoptera: Carabidae)	BO, endemic, role not quantified, NC
Notiobia schnusei Emden (Coleoptera: Carabidae)	BO endemic, role not quantified, NC
Notocyrtus dorsalis (Gray) (Hemiptera: Reduviidae)	PY endemic, role not quantified, NC
Nylanderia fulva (Mayr) (Hymenoptera: Formicidae)	FA endemic, role not quantified, NC

Ocyptamus (Diptera: Syrphidae)	FA endemic, role not quantified, NC
Ocyptamus clava (Fabricius) (Diptera: Syrphidae)	VE endemic, role not quantified, NC
Ocyptamus dimidiatus (Fabricius) (Diptera: Syrphidae)	VE endemic, role not quantified, NC
Ocyptamus gastrostactus (Wiedemann) (Diptera: Syrphidae)	VE endemic, role not quantified, NC
Ocyptamus stenogaster (Williston) (Diptera: Syrphidae)	VE endemic, role not quantified, NC
Oeneis nigrans Muls (Coleoptera: Coccinellidae)	SR endemic, role not quantified, NC
Oligota minuta Cameron (Coleoptera: Staphylinidae)	CO, RC, SR endemic, reduces pest, NC
Oligota sp. (Coleoptera: Staphylinidae)	FA endemic, role not quantified, NC
Olla v-nigrum (Mulsant) (Coleoptera: Coccinellidae)	CO, CR, MX, PY, VE, endemic, good control, ABC, NC
Orius (Hemiptera: Anthocoridae)	NI, endemic, role not quantified, NC
Orius euryale Herring (Hemiptera: Anthocoridae)	NI, endemic, role not quantified, NC
Orius insidiosus (Say) (Hemiptera: Anthocoridae)	AR, BB, BR,CO, DO, FA, HN, MX, NI, PA, PE, PY, UY, TT, VE endemic, good control, ABC, NC
Orius laevigatus (Fieber) (Hemiptera: Anthocoridae)	CO, DO, JM, MX, exotic, ?established, good control, ABC
Orius pumilio (Champion) (Hemiptera: Anthocoridae)	FA, endemic, role not quantified, NC
Orius sp. (Hemiptera: Anthocoridae)	CO, CU, MX, PY, endemic, reduces pest, NC/ABC/ConsBC
Orius spp. (Hemiptera: Anthocoridae)	NI, endemic, role not quantified, ABC, NC
Orius tristicolor (White) (Hemiptera: Anthocoridae)	CO, MX, VE, endemic, reduces pest, ABC, NC
Ornidia major Curran (Diptera: Syrphidae)	VE endemic, role not quantified, NC
Ornidia obesa F. (Diptera: Syrphidae)	VE endemic, role not quantified, NC
Orthoderella ornata Giglio-Tos (Mantodea: Mantidae)	PY endemic, role not quantified, NC
Pachylister chinensis Quensel (Coleoptera: Histeridae)	BB, RC, exotic, not established, no control, CBC
Paederus sp. (Coleoptera: Staphylinidae)	PY, VE endemic, role not quantified, NC
Palpada mexicana (Macquart) (Diptera: Syrphidae)	VE endemic, role not quantified, NC
Palpada pusila (Macquart) (Diptera: Syrphidae)	VE endemic, role not quantified, NC
Palpada ruficeps (Macquart) (Diptera: Syrphidae)	VE endemic, role not quantified, NC
Palpada solennis (Walter) (Diptera: Syrphidae)	VE endemic, role not quantified, NC
Pentilia castanea Muls (Coleoptera:Coccinellidae)	DO, exotic, not established, CBC, SR, endemic?, good control, CBC
Pentilia egena Muls (Coleoptera: Coccinellidae)	RC, exotic, not established, CBC
Pentilia insidiosa Mulsant (Coleoptera: Coccinellidae)	BB, RC exotic, established? No data about control effect, CBC

Pentilia spp. (Coleoptera: Coccinellidae)	RC exotic, established? No data about control effect, CBC
Pheidole fallax Mayr (Hymenoptera: Formicidae)	FA endemic, role not quantified, NC
Pheidole megacephala (F.) (Hymenoptera: Formicidae)	CU, PR, endemic, good control, ConsBC, NC
Pheidole radoszkowskii (Forel) (Hymenoptera: Formicidae)	CR, endemic, reduce pest, NC
Phelister rufinotus Marseul (Coleoptera: Histeridae)	PY endemic, role not quantified, NC
Pheropsophus aequinoctialis L. (Coleoptera: Carabidae)	BO, endemic, reduces pest, ABC
Philonthus quadraticeps Boheman (Coleoptera: Staphylinidae)	AR, exotic, not established, CBC
Phlugis teres DeGeer (Orthoptera: Tettigoniidae)	VE endemic, role not quantified, NC
Phymata fasciata (Gray) (Hemiptera: Reduviidae)	PY endemic, role not quantified, NC
Phymata sp. aff. *fortificata* (Herrich-Schäffer) (Hemiptera: Reduviidae)	PY endemic, role not quantified, NC
Phytoseiulus longipes Evans (Acari: Phytoseiidae)	BR, endemic, good control, ABC, NC
Phytoseiulus macropilis Banks (Acari: Phytoseiidae)	BR, endemic, good control, ABC, NC
Phytoseiulus persimilis Athias-Henriot (Acari: Phytoseiidae)	CO, DO, EC, MX, PE, endemic, good control with imported stains, ABC, NC
Phytoseiulus sp. (Acari: Phytoseiidae)	EC, exotic, good control, ABC
Phytoseius dominicensis Ferragut & Moraes sp. nov. (Acari: Phytoseiidae)	DO, endemic, role not quantified, NC
Pitangus sulphuratus sulphuratus (L.) (Passeriformes: Tyrannidae)	SR endemic, role not quantified, NC
Plaesius javanus Erichson (Coleoptera: Histeridae)	CU, DM, JM, RC, exotic, not established, JM, MX, exotic, established, reduces pest, CBC
Plesiometa argyra (Walckenaer) (Araneae: Tetragnathidae)	CR, endemic, role not quantified, NC
Podisus connexivus Bergroth (Hemiptera: Pentatomidae)	EC, endemic, reduces pest, ABC
Podisus maculiventris (Say) (Hemiptera: Pentatomidae)	MX, exotic, used in ABC, reduces pest, no info about establish
Podisus nigrispinus (Dallas) (Hemiptera: Pentatomidae)	BR, PE, endemic, reduces pest, ABC
Podisus sp. (Hemiptera: Pentatomidae)	PY, endemic, role not quantified, NC
Poecilia reticulata Rosen & Bailey (Cyprinodontiformes: Poeciliidae)	CO, endemic, predatory fish, reduces pest, ABC, South America: Venezuela, Barbados, Trinidad, northern Brazil and the Guyanas.; may have negative effect on native fish species
Polistes canadensis (Linné) (Hymenoptera: Vespidae)	PY, endemic, role not quantified, NC
Polistes canadensis infuscatus Lep. (Hymenoptera: Vespidae)	SR, endemic, reduces pest, NC
Polistes cavapyta Saussure (Hymenoptera: Vespidae)	PY endemic, role not quantified, NC

Polistes cinctus cinctus Lepeletier (Hymenoptera: Vespidae)	RC endemic, reduces pest, ConsBC, NC
Polistes cinctus barbadensis Richards (Hymenoptera: Vespidae)	RC endemic, role not quantified, NC
Polistes crinitus (Felton) (Hymenoptera: Vespidae)	DO endemic, role not quantified, NC
Polistes infuscatus ecuadorius Richards (Hymenoptera: Vespidae)	EC, endemic, reduces pest, ConsBC, NC
Polistes panamensis Holmgren (Hymenoptera: Vespidae)	PA endemic, role not quantified, NC
Polistes sp. (Hymenoptera: Vespidae)	BO, PR, PY endemic, role not quantified, NC
Polistes spp. (Hymenoptera: Vespidae)	FA, RC, endemic, role not quantified, NC
Polistes versicolor (Olivier) (Hymenoptera: Vespidae)	PY, VE, endemic, role not quantified, NC
Polistes versicolor vulgaris Beq. (Hymenoptera: Vespidae)	SR, endemic, reduces pest, NC
Polybia chrysothorax (Web.) (Hymenoptera: Vespidae)	SR, endemic, reduces pest, NC
Polybia ignobilis (Haliday) (= *Polybia atra* Saussure) (Hymenoptera: Vespidae)	PY, endemic, role not quantified, NC
Polybia liliacea (F.) (Hymenoptera: Vespidae)	SR endemic, reduces pest, NC
Polybia nigra Saussure (Hymenoptera: Vespidae)	VE endemic, role not quantified, NC
Polybia occidentalis (Olivier) (Hymenoptera: Vespidae)	PY, endemic, role not quantified, NC
Polybia paulista Ihering (Hymenoptera: Vespidae)	PY, endemic, role not quantified, NC
Polybia rejecta (F.) (Hymenoptera: Vespidae)	SR endemic, reduces pest, NC
Polybia scutellaris (White) (Hymenoptera: Vespidae)	PY, endemic, role not quantified, NC
Polybia sericea (Oliv.) (Hymenoptera: Vespidae)	PY, endemic, role not quantified, NC, SR , endemic, reduces pest, NC
Polybia sp. (Hymenoptera: Vespidae)	CO, PY, endemic, role not quantified, NC
Polybia striata (F.) (Hymenoptera: Vespidae)	SR , endemic, reduces pest, NC
Prodilis (Neoporia) sp. (Coleoptera: Coccinellidae)	JM endemic, role not quantified, NC
Prodilis sp. (Coleoptera: Coccinellidae)	BB, endemic, role not quantified, NC
Proprioseiopsis sandersi (Chant) (Acari: Phytoseiidae)	DO, endemic, role not quantified, NC
Proprioseiopsis sp. (Acari: Phytoseiidae)	PA endemic, role not quantified, NC
Pseudoazya (Azya) trinitatis (Marshall) (Coleoptera: Coccinellidae)	JM, exotic, established, reduces pest, ABC, CBC, RC, exotic, not established
Pseudoazya trinitatis Gordon (Coleoptera: Coccinellidae)	BB, exotic, established, role not quantified, CBC
Pseudodorus clavatus (F.*)* (Diptera: Syrphidae)	DO, FA, PY, VE, endemic, role not quantified, NC
Pseudoparasitus sp. (Acari: Laelapidae)	PA endemic, role not quantified, NC
Psyllobora divisa (F.) (Coleoptera: Coccinellidae)	SR, endemic, role not quantified, NC

Pterostichus sp. (Coleoptera: Carabidae)	BO, endemic, role not quantified, NC
Pullus gilae (Casey) (Coleoptera: Coccinellidae)	PY endemic, role not quantified, NC
Pullus loewii Mulsant (Coleoptera: Coccinellidae)	PY endemic, role not quantified, NC
Pullus spp. (Coleoptera: Coccinellidae)	PY endemic, role not quantified, NC
Pyemotes ventricosus (Newport) (Acarina: Pyemotidae)	BB, MX, endemic, reduces pest, ABC, NC
Pyrophorus luminosus Illiger (Coleoptera: Elateridae)	BB, exotic, established, no control, PR, endemic, reduced pest, NC
Quichuana picadoi Knab (Diptera: Syrphidae)	VE endemic, role not quantified, NC
Repipta sp. (Hemiptera: Reduviidae)	PY, endemic, role not quantified, NC
Rhinella marina L. (Anura: Bufonidae)	DO, PR exotic, established, good control, CBC, but negative eff.
Rhingia nigra Mcquart (Diptera: Syrphidae)	VE, endemic, role not quantified, NC
Rhinoleucophenga sp. (Diptera: Drosophilidae)	RC, exotic, not established
Rhyzobius lophanthae (Blaisd) (Coleoptera: Coccinellidae)	UY, exotic, established, good control, ABC
Rhyzobius purchellus Montrozier (Coleoptera: Coccinellidae)	PE, RC, exotic, ?established, role unknown, CBC
Rhyzobius ventralis (Erichson) (Coleoptera: Coccinellidae)	CL, exotic, ?established, role unknown, CBC
Rodolia (Novius) cardinalis Mulsant (Coleoptera: Coccinellidae)	AR, BB, BO, CO, CU, DO, EC, FA, JM, PE, PR, RC, UY, VE, exotic, good control, CBC
Rostrhamus sociabilis (Vieillot) (Accipitriformes: Accipitridae)	SR, endemic, good control, NC, bird
Saimiri sciureus (L.) (Primatas: Cebidae)	SR, endemic, reduces pest, NC, monkey
Salpingogaster nigra Shiner (Diptera: Syrphidae)	CR, GY, MX, SR, TT, VE, endemic, reduces pest, NC, ABC
Scymnus coccivora Aiyyar (Coleoptera:Coccinellidae)	TT, exotic, reduced pest, CBC, RC exotic, role unknown
Scymnus rubicundus Erichson (Coleoptera: Coccinellidae)	CO, endemic, role not quantified, NC
Scymnus smithianus Silvestri (Coleoptera: Coccinellidae)	CU, JM exotic, ?established, no control results
Scymnus sp. (Coleoptera: Coccinellidae)	BB, CR, endemic, role not quantified, NC, DO endemic, reduces pest, NC, JM, PA, PE, PY, SR, endemic, reduces pest, NC, RC, exotic, no results
Scymnus spp. (Coleoptera: Coccinellidae)	JM, endemic, reduced pest, NC, VE, endemic, role not quantified, NC
Sinea sp.(Hemiptera: Reduviidae)	MX, endemic, reduces pest, ABC, PY, endemic, role not quantified, NC
Sirentha carinata (Fabricius) (Hemiptera: Reduviidae)	BO, endemic, reduces pest, CBC/NC
Sphiximorpha barbipes (Loew) (Diptera: Syrphidae)	VE, endemic, role not quantified, NC

Stethorus caribus Gordon & Chapin (Coleoptera: Coccinellidae)	DO, endemic, role not quantified, NC
Stethorus punctillum (Weise) (Coleoptera: Coccinellidae)	MX, exotic, no further info
Stethorus salutaris Kapur (Coleoptera: Coccinellidae)	JM, exotic, not established
Stethorus sp. (Coleoptera: Coccinelidae)	CO, DO, PE, endemic, reduces pest, NC, ABC
Stratiolaelaps scimitus (Hypoaspis miles) Womersley (Acari: Laelapidae)	BR, CO endemic, good control, ABC,
Sympherobius barberi (Banks) (Neuroptera: Hemerobiidae)	PE, endemic, reduces pest, CBC/ABC
Sympherobius sp. (Neuroptera: Hemerobiidae)	PY, endemic, role not quantified, NC
Syneura cocciphila (Coquillett) (Diptera: Phoridae)	AR, exotic, ?established, CO endemic, role not quantified, NC
Synoeca cyanea (Fabricius) (Hymenoptera: Vespidae)	PY, endemic, role not quantified, NC
Syrphus (Diptera: Syrphidae)	FA, endemic, role not quantified, NC
Systoechus vulgaris Loew (Diptera: Bombyliidae)	AR, exotic, not established,
Telsimia nitida Chapin (Coleoptera: Coccinellidae)	RC, exotic, not established
Telsimia sp. (Coleoptera: Coccinellidae)	BB, SV, exotic, not established
Termatophylidea opaca Carvalho (Hemiptera: Miridae)	SR, endemic, role not quantified, NC
Thalassa montesumae Mulsant (Coleoptera: Coccinellidae)	HN, endemic, reduces pest, ABC/NC
Theridula gonygaster (Simon) (Araneae: Theridiidae)	JM, endemic, reduces pest, NC
Toxomenus floralis (F.) (Diptera: Syrphidae)	PY, VE, endemic, role not quantified, NC
Toxomerus (Diptera: Syrphidae)	FA, VE, endemic, role not quantified, NC
Toxomerus sp. cf. *watsoni* (Curran) (Diptera: Syrphidae)	PY, endemic, role not quantified, NC
Toxorhynchites brevipalpis (Theobald) (Diptera: Culicidae)	RC, endemic, good control, ABC, NC
Tupiocoris cucurbitaceus (Spinola 1852) (Hemiptera: Miridae)	CL, endemic, good control, ABC, NC
Tupiocoris notatus (Distant) (Hemiptera: Miridae)	DO, endemic, reduces pest, NC, mentions risk but not measured
Typhlodromina (Acari: Phytoseiidae)	DO, endemic, reduces pest, NC
Typhlodromips (= *Amblyseius*) *swirskii* (Athias-Henriot) (Acari: Phytoseiidae)	DO, EC, HO, MX, UY, exotic, good control of pest, ABC
Typhlodromus citri Garman & McG (Acari: Phytoseiidae)	JM, exotic, not established
Typhlodromus occidentalis (= *Galandromus*) Nesbitt (Acari: Phytoseiidae)	JM, exotic, not established
Typhloseiopsis adventitius Ferragut & Moraes sp. Nov (Acari: Phytoseiidae)	DO, endemic, role not quantified, NC
Tytthus mundulus (Breddin) (Hemiptera: Miridae)	EC, JM, exotic, reduces pest, CBC
Tytthus parviceps Reuter (Hemiptera: Miridae)	EC, endemic, reduces pest, ABC, NC

Xanthopygus cognatus Sharp (Coleoptera: Staphylinidae)	SV, endemic, reduces pest, NC
Xylocoris flavipes (Reuter) (Hemiptera: Anthocoridae)	MX, exotic, role not quantified
Zagloba aenipennis (Sicard) (Coleoptera: Coccinellidae)	BB, exotic, ?established, CBC
Zagreus (= Exochomus) *bimaculosus* Mulsant (Coleoptera: Coccinellidae)	FA, endemic, role not quantified, NC
Zelus argillaceum (L.) (Hemiptera: Reduviidae)	PY, endemic, role not quantified, NC
Zelus armillatus (Lepeletier and Serville) (Hemiptera: Reduviidae)	PY, endemic, role not quantified, NC
Zelus cf. *nugax* Stål (Hemiptera: Reduviidae)	CO, endemic, role not quantified, NC
Zelus illotus Berg (Hemiptera: Reduviidae)	PY, endemic, role not quantified, NC
Zelus janus Stal (Hemiptera: Reduviidae)	MX, endemic, reduced pest, ABC, NC
Zelus laticornis (Herrich-Schäffer) (Hemiptera: Reduviidae)	PY, endemic, role not quantified, NC
Zelus leucogrammus (Perty) (Hemiptera: Reduviidae)	PY, endemic, role not quantified, NC
Zelus longipes (L.) (Hemiptera: Reduviidae)	FA, PY, endemic, role not quantified, NC
Zelus ruficeps Stål (Hemiptera: Reduviidae)	PY, endemic, role not quantified, NC
Zelus sp. (Hemiptera: Reduviidae)	DR, PY, endemic, role not quantified, NC
Zelus spp. (Hemiptera: Reduviidae)	PY, endemic, role not quantified, NC
Zenoria emarginata Gordon (Coleoptera: Coccinellidae)	RC, exotic, not established
Zeta argillaceum (L.) (Hymenoptera: Vespidae)	PY, endemic, role not quantified, NC

Country abbreviations: AR = Argentina, BB = Barbados, Belize = BZ, Bolivia = BO, Brazil = BR, Chile = CL, Colombia = CO, Costa Rica = CR, Cuba = CU, Dominica = DM, Dominican Republic = DO, Ecuador = EC, El Salvador = SV, French Guiana, Guadeloupe and Martinique = FA, Guatemala = GT, Guyana = GY, Haiti = HT, Honduras = HN, Jamaica = JM, Mexico = MX, Nicaragua = NI, Panama = PA, Paraguay = PY, Peru = PE, Puerto Rico = PR, Remaining Caribbean Islands = RC, Suriname = SR, Trinidad and Tobago = TT, Uruguay = UY, Venezuela = VE
