# Supplementary material for: Benefits and Risks of Native and Exotic Biological Control Agents Used in Latin America and the Caribbean: Performance of 1099 Arthropod Natural Enemies
Source: Neotrop Entomol. 2026 Jul 28;55(1):69. doi: 10.1007/s13744-026-01412-8 (PMC13415494; doi:10.1007/s13744-026-01412-8)
Supplement: Supplementary file 4 — (PDF 154 KB) [file 13744_2026_1412_MOESM4_ESM.pdf]

Tables SI4 and 5. Benefits and risks of endemic and exotic biological control agents used in Latin America and the Caribbean: performance of 1099 arthropod natural enemies.

Joop C. van Lenteren, Vanda H. P. Bueno, Wageningen University, Laboratory of Entomology, Department of Plant Sciences, 6700 AA, Wageningen, The Netherlands, joop.vanlenteren@wur.nl

Table SI4. Number of species of organisms mentioned in van Lenteren et al. (2020), number of native and exotic parasitoids and predators used in biological control in Latin America and the Caribbean with and without a pest-reducing effect, and number of natural enemy species with negative side effects (ABC=augmentative biological control, CBC=classical biological control, ConsBC=conservation biological control, NBC=natural biological control)

**Species total**	
Total number of species of plants and animals mentioned	2413
Total number of natural enemies	1099
Total number of invertebrate natural enemies	1087
Total number of parasitoids	673
Total number of invertebrate predators	414
**Parasitoids total**	673*
Native parasitoids	382**
With pest-reducing effect	153***
In NBC	153
In ConsBC	4
In ABC	45
Without pest-reducing effect	229
Native parasitoids with negative non-target effect	0
Exotic parasitoids	338**
Exotic parasitoids established	191
With pest-reducing effect	168***
In CBC	117
In ABC	67
Without pest-reducing effect	23
Exotic parasitoids not established or unknown	147
Exotic parasitoid with negative non-target effect	0
**Predators total**	426*
Native predators all (including vertebrates)	317
Native vertebrate predators	10
With pest-reducing effect	10***
In NBC	10
In ConsBC	2
Native invertebrate predators	307
With pest-reducing effect	307***
In NBC	307
In ConsBC	9
In ABC	65
Native invertebrate predator with documented negative non-target effect	0

Native invertebrate predator with possible negative non-target effect	1****
**Exotic predators** all (including vertebrates)	116
Exotic vertebrate predators	2
Established	2
With pest-reducing effect	2
Exotic vertebrate predators with documented negative non-target effect	2
Exotic invertebrate predators	114
Exotic invertebrate predator species established	55
With pest-reducing effect	43***
In CBC with documented effect	29
In ABC with documented effect	31
Without pest-reducing effect	12
Exotic invertebrate predators not established or unknown	59
Exotic invertebrate predator with negative non-target effect	1*****

* the total number of species of parasitoids/predators is lower than number of exotic + number of native species because some species are exotic in one and native in another country
** the total number of native or exotic species of parasitoids /predators is lower than the sum of the subcategories, because some species play a role in different types of BC
*** the total number of species with a pest-reducing effect is lower than the sum of the subcategories, because some species play a role in different types of BC
**** possible negative side effects of zoophytophagous heteropteran predators are mentioned based on such effects being perceived in other world regions, but negative effects of these predators have not been reported from Latin America and the Caribbean
***** negative non-target effects have been reported for the predator *Harmonia axyridis* by several Latin American and Caribbean countries

Table SI5. BIOCAT2010.3 data on classical biological control of insect pests using exotic insect biological control agents in Latin America and the Caribbean (Cock 2019).

Number of natural enemy species introduced	387
Number of natural enemy species established	128
Number of natural enemy species with pest-reducing effect	57
Number of pest species targeted	118
Number of pest species controlled	40
Number of natural enemies with negative non-target effect	0
